# **Mental health symptoms during the COVID-19 pandemic in developing countries**: **A systematic review and meta-analysis**

**DOI:** 10.7189/jogh.12.05011

**Published:** 2022-05-23

**Authors:** Jiyao Chen, Stephen X Zhang, Allen Yin, Jaime A Yáñez

**Affiliations:** 1 College of Business, Oregon State University, Corvallis, Oregon, USA; 2 Adelaide Business School, University of Adelaide, Adelaide, Australia; 3 School of Humanities, Southeast University, Nanjing, China; 4 Vicerrectorado de Investigación, Universidad Norbert Wiener, Lima, Peru; 5 Gerencia Corporativa de Asuntos Científicos y Regulatorios, Teoma Global, Lima, Peru

## Abstract

**Background:**

This systematic review aims to 1) summarize the prevalence of anxiety, depression, distress, insomnia, and PTSD in the adult population during the first year of the COVID pandemic in developing countries and 2) uncover and highlight the uneven distribution of research on mental health in all developing countries across regions.

**Methods:**

Several literature databases were systemically searched for meta-analyses published by September 22, 2021, on the prevalence rates of mental health symptoms in developing countries worldwide. We meta-analysed the raw data of the individual empirical results from the previous meta-analysis papers in developing countries in different regions.

**Results:**

The prevalence rates of mental health symptoms were summarized based on 341 empirical studies with a total of 1 704 072 participants from 40 out of 167 developing countries in Africa, Asia (East, Southeast, South, and West), Europe, and Latin America. Comparatively, Africa (39%) and West Asia (35%) had the worse overall mental health symptoms, followed by Latin America (32%). The prevalence rates of overall mental health symptoms of medical students (38%), general adult students (30%), and frontline health care workers (HCWs) (27%) were higher than those of general HCWs (25%) and general populations (23%). Among five mental health symptoms, distress (29%) and depression (27%) were the most prevalent. Interestingly, people in the least developing countries suffered less than those in emergent and other developing countries. The various instruments employed lead to result heterogeneity, demonstrating the importance of using the well-established instruments with the standard cut-off points (eg, GAD-7, GAD-2, and DASS-21 for anxiety, PHQ-9 and DASS-21 for depression, and ISI for insomnia).

**Conclusions:**

The research effort on mental health in developing countries during COVID-19 has been highly uneven in the scope of countries and mental health outcomes. This meta-analysis, the largest on this topic to date, shows that the mental health symptoms are highly prevalent yet differ across regions. The accumulated systematic evidence from this study can help enable the prioritization of mental health assistance efforts to allocate attention and resources across countries and regions.

The COVID-19 pandemic has truly been a global pandemic, particularly impactful in developing countries due to the lack of resources and initiatives for tackling mental health issues in the already overburdened and fragile health care systems [1,2]. For instance, the developing country of Bangladesh has a psychiatrist-population ratio of 0.13 per 100 000, far below the recommended range of 3.8 to 15.8 per 100 000 [3]. The overburdened and fragile health care systems and slower or non-existent access to vaccination impose psychological stress on ordinary citizens, who suffer under restrictions and lockdowns for multifaceted reasons including, but not limited to, family separations, reduced social contact, less unemployment, work stress, increased loneliness, the overabundance of (mis)information on social media, and various other related factors [4-6]. Global assessments of mental health symptoms under the COVID-19 pandemic across developing countries remain a challenging yet vital endeavour [7].

However, developing countries lack large-scale monitoring of the prevalence of mental health symptoms at the national level, as implemented in developed countries such as the USA and the UK. Instead, developing countries can only rely on individual primary studies from independent researchers (eg, [8], and as a result, there is an uneven geographical distribution of such studies under the COVID-19 pandemic [9]. For instance, a review revealed only 12 out of the 48 countries in Africa have been researched on mental health during COVID-19 [10]. To map the status of mental health in developing countries worldwide, this study aims to summarize the prevalence of anxiety, depression, distress, insomnia, and PTSD in their adult populations under the COVID pandemic. The second aim of this study is to uncover and highlight the uneven distribution of research on mental health across all developing countries across regions.

## METHODS

To achieve such aims, we systematically searched and leveraged the existing meta-analyses on the topic to identify individual studies on the prevalence rates of mental health symptoms in developing countries worldwide. We aimed to reveal the inadequacy, if not the absence of, studies in certain countries, to highlight the need to initiate relevant research on the neglected countries. We also conducted a meta-regression to assess the prevalence rates of anxiety, depression, and insomnia in developing countries across regions, based on the two categories of developing countries in terms of the level of development (least developing countries vs emergent and other developing countries). We wanted to reveal heterogeneities to enable evidence-based health care assistance and resource allocation across developing countries, which continue to struggle under the COVID-19 pandemic.

This systematic review and meta-analysis were conducted in accordance with the Preferred Reporting Items for Systematic Reviews and Meta-Analyses (PRISMA) statement 2019 and registered in the International Prospective Register of Systematic Reviews (PROSPERO: CRD42020220592).

### Data sources and search strategy

This study is built upon the existing meta-analyses; we contacted their authors to ask for their coding data for aggregation, since relevant meta-analyses already exist at the scale of individual countries or regions. We searched PubMed, Embase, PsycINFO, Web of Science, medRxiv, and Google Scholar in English for meta-analyses on mental health symptoms of the key adult populations during COVID-19 from January 1, 2020 to September 22, 2021. For example, the following Boolean operators on three sets of keywords were used in Web of Science:

- (ALL= ((2019-nCoV OR 2019nCoV OR COVID-19 OR SARS-CoV-2 OR (Wuhan AND coronavirus)) AND (“depressi*” OR “anxi*” OR “insomnia” OR “sleep” OR “distress” OR “PTSD” OR “post-traumatic stress disorder” OR “mental health” OR “psychiatric”))) AND (TS= “meta-analysis”).

The search targeted meta-analyses that focused on the prevalence of anxiety, depression, distress, insomnia, and PTSD in specific regions or countries during COVID-19. Meta-analyses that did not specify regions or countries were excluded. When multiple meta-analyses existed on the same region, the most comprehensive analysis was chosen. We contacted the authors of these meta-analyses to request their original coding data. Figure S1 in the **Online Supplementary Document** details the flowchart of the overall search process.

### Selection criteria

To be included in this review, the evidence must have studied the prevalence of at least one mental symptom outcome (eg, anxiety, depression, distress, insomnia, and PTSD) of adult populations such as frontline health care workers (HCW), general HCWs, general adult population, medical students, and general adult students in any developing countries based on the definition of [11] during the COVID-19 pandemic, published in English.

We excluded empirical studies using the following criteria:

1. Population: children, adolescents, or specific niche adult populations such as COVID-19 patients, inpatients, or other patients, adults under quarantine, or pregnant/postpartum women in developing countries
2. Methodological approaches: non-primary studies such as reviews, qualitative or case studies, interventional studies, interviews, or news reports
3. Measurements: non-validated mental health instruments (i.e., self-made questionnaires) or instruments without a validated cut-off score to calculate a prevalence rate (i.e., STAI, SCL-90 for anxiety and depression).

### Selection process and data extraction

The coding data from the original meta-analyses were reviewed and recoded based on a single pre-developed coding protocol to ensure the consistency and comparability of the results [10]. If the authors of the existing meta-analyses did not share the coding data, we identified their original empirical studies, independently extracted the relevant data into a coding book based on the same coding protocol [10] and cross-checked their coding. We assessed the eligibility of each study by reading its full text to remove redundant empirical studies and code relevant information such as the authors and year of the study, title, publication status, sample locations, date of data collection, sample size, response rate, population, age (mean, SD, min, and max), gender proportion, instruments, cut-off scores used, the prevalence/mean/SD of the mental health outcome, and other notes or comments. In cases where the two coders disagreed, a lead researcher checked the study independently to determine its coding. The lead researcher integrated all the coding information and reviewed the key information such as mental outcomes, instruments, outcome levels, prevalence, population, sample, and regions.

To consistently analyse the data, we verified the independence of mental health symptoms and samples. For instance, if a study used more than one instrument to measure a mental health outcome, we reported the results based on the most popular instrument. We used the three typical cut-off levels of mental health symptoms (above mild, above moderate, and severe) as standards for reporting the prevalence above mild, moderate, and severe levels. If an empirical study reported the prevalence rates differently from the three-level norm with cut-off points, such as at overall level, we converted the prevalence rates into above mild, above moderate, or severe based on the typical cut-off points of the instruments used.

### Assessment of bias risk

The Mixed Methods Appraisal Tool (MMAT) was used as a quality assessment tool [12-14]. Two reviewers independently assessed scores (ranging from 0 [low] to 7 [high]) for the studies using the tool dictionary and guidelines, crosschecked their coding, and resolved disagreements. Studies were categorized as high, medium, or low quality based on the score of >6, 5-6, or <5, respectively.

### Statistical analysis

A random-effects model was used (the *metaprop* package in version 16.1 of Stata) to compute the pooled estimates of outcome prevalence between populations by assuming that these studies were randomly selected from their targeted populations [15].

Given the high degree of heterogeneity of the true differences in the effect sizes [16], we ran a meta-regression to regress the prevalence upon outcomes (five types of mental health symptoms), severity of outcome (above mild/above moderate/severe), five major population groups (frontline HCWs, general HCWs, general population, adult students, medical students), and on continents or regions, sample size, research design, and study quality. Given the size of Asia, which contains 60% of the world population, we used the sub-continental regions of Asia (Central, East, Southeast, West, and South). The other continents were not subdivided due to being smaller both in terms of populations as well as the number of conducted studies, so that the regions do not contain too few samples.

The meta-analytical results of our study enable the prediction of prevalence rates while accounting for multiple factors at the same time, thus offering a superior model over prior meta-analyses, which accounted for predictors separately [17,18]. Hence, based on the results of meta-regression, we predicted the prevalence rates of anxiety, depression, and insomnia symptoms at mild above, moderate above, and severe for frontline HCWs, general HWCs, general population, and general students in the seven regions. Due to small sample size, we did not predict the prevalence rates on distress and PTSD or medical students. The statistical significance is taken at the 95% confidence interval level.

The DOI plot and the Luis Furuya-Kanamori index [19] were constructed to assess publication bias [20,21]. We used event ratio as the primary effect measure for the pooled estimates.

## RESULTS

### Study screening

The search generated smaller elementary meta-analyses on mental health symptoms during COVID-19 [9,10,18,22-29], and we were able to obtain the original coding results from seven of them. The aggregation resulted in a total of 461 studies, 341 of which were unique studies that fit the criteria for this meta-analysis of developing countries (Figure S1 in the **Online Supplementary Document**).

### Study characteristics

**Table 1** summarizes the study characteristics of the 341 empirical studies with a total of 1 704 072 participants in 404 samples. Out of the 404 samples, 73 (18.07%) were from frontline HCWs, 126 (31.19%) from general HCWs, 145 (35.85%) from the general population, 43 (10.84%) from adult students, and 17 (4.21%) from medical students. Table S1 in the **Online Supplementary Document** reported the study characteristics of each of the 341 studies. Among the 404 samples, about one-third investigated the general population (35.85%) or general HCWs (31.19%), almost one-fifth studied frontline HCWs (18.07%), one-tenth focused on general adult students (10.64%), and only 4.21% studied medical students.

**Table 1:** Characteristics of the studies on mental health in developing countries during the COVID-19 pandemic

Characteristics	Total number of studies/samples*	Percentage (%)	Level of analysis
**Overall**	341/404	100.00	
Population	404	100.00	Sample
Frontline HCWs	73	18.07	
General HCWs	126	31.19	
General population	145	35.85	
Adult students	43	10.64	
Medical students	17	4.21	
**Outcome †:**	1433	100.00	Prevalence
Anxiety	650	45.36	
Depression	551	38.45	
Distress	38	2.65	
Insomnia	158	11.03	
PTSD	36	2.51	
**Severity:**	1433	100.00	Prevalence
Mild above	557	38.87	
Moderate above	542	37.82	
Overall	21	1.47	
Severe	313	21.84	
**Region:**	343^†^	100.00	Study
Africa	29	8.50	
**Asia:**			
East Asia	147	43.11	
South Asia	48	14.08	
Southeast Asia	20	5.87	
West Asia	18	5.28	
Europe	18	5.28	
Latin America	61	17.89	
Oceania	0	0.00	
**Design:**	341	100.00	Study
Cross-sectional	329	96.48	
Cohort	12	3.52	
Publication status:	341	100.00	Study
Preprint	15	4.40	
Published	326	95.60	
**Quality:**	341	100	Study
High	48	14.80	
Medium	235	68.91	
Low	58	17.10	
	Medium (Mean)	Range	
Number of participants	535 (4218)	257-1252	Sample
Female portion	65.55% (64.57%)	12.10%-76.10%	Study
Response rate	83.30% (75.04%)	63%-95.12%	Study

PTSD – Posttraumatic stress disorder

*A study may include multiple independent samples. An independent sample in a study may report anxiety, depression, and insomnia at the levels of mild above, moderate above, and severe. Hence, the total number of prevalence rates is larger than the total number of independent samples.

†Two studies included studies from different regions.

More than 80% of the studies covered anxiety and depression symptoms (45.36% and 38.87%, respectively). Just over one-tenth investigated insomnia symptoms (11.03%); few studies investigated PTSD (2.65%) and distress (2.51%). The studies reported the prevalence rates using cut-offs at the “mild above” (38.87%), “moderate above” (37.82%), and “severe above” (21.84%) level of symptom severity.

Almost all studies, 329 in total, employed cross-sectional surveys, with only 12 cohort studies (3.52%). The MMAT indicated 48 (14.08%) studies were of good quality (score >6/7), 235 (68.91%) studies were of medium quality (score = 5-6), and 58 (17.01%) studies were of low quality (score <5). The median number of individuals per sample was 535 (range = 19-746 217).

**Figure 1:**
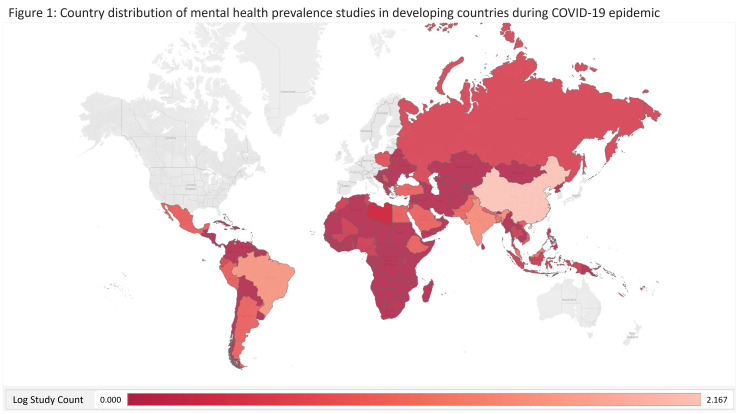
Country distribution of mental health prevalence studies in developing countries during COVID-19 pandemic.

We next break down the studies by country and region. **Table 2** and **Figure 1** show that, in total, there were 50 countries from four continents with at least one mental health prevalence study under COVID-19: Africa (11 countries studied out of 56 developing countries), Asia (17 countries studied out of 49 developing countries), Europe (nine countries studied out of 15 developing countries), and Latin America (13 countries studied out of 37 developing countries). There have been no studies in the continent of Oceania (zero countries out of 10 developing countries). Within Asia, there have been studies in East Asia, Southeast Asia, South Asia, and West Asia, but not in Central Asia (five developing countries). However, few studies collected samples from multiple countries or regions. For example, one study [30] collected data from both Africa and Latin America, while another [31] collected data from five Southeast and South Asia countries. Overall, our analysis contains seven regions: Africa, East Asia, Europe, Latin America, South Asia, Southeast Asia, and West Asia.

**Table 2:** Country distribution of mental health prevalence studies in developing countries during COVID-19 pandemic

Continent*	Region	Country‡	n of study	Percentage
**Africa**		11 countries studied out of 56 developing countries	29	8.45
	Northern Africa		13	3.78
		Egypt	6	1.75
		Libya	3	0.87
		Morocco	2	0.58
		Tunisia	2	0.58
	Sub-Saharan Africa		16	4.67
		Cameroon	2	0.58
		Democratic Republic of the Congo	1	0.29
		Ethiopia	7	2.04
		Mali	2	0.58
		Nigeria	2	0.58
		South Africa	1	0.29
		Togo	2	0.58
**Asia**		17 countries studied out of 49 developing countries	236	68.22
	Central Asia	0 countries studied out of 5 developing countries	0	0.00
	East Asia	1 country studied out of 6 developing countries		
		China	147	42.86
	Southeast Asia	5 countries studied out of 9 developing countries	20	5.83
		Indonesia	2	0.58
		Malaysia	10	2.92
		Philippines	1	0.29
		Thailand	2	0.58
		Vietnam	5	1.46
	South Asia	5 countries studied out of 9 developing countries	49	14.29
		Bangladesh	10	2.92
		India	24	7.00
		Nepal	6	1.75
		Pakistan	8	2.33
		Sri Lanka	1	0.29
	West Asia	6 countries studied out of 18 developing countries	20	5.83
		Saudi Arabia	1	0.29
		Jordan	2	0.58
		Kuwait	1	0.29
		Oman	1	0.29
		Saudi Arabia	6	1.75
		Turkey	7	2.04
**Europe**	Eastern Europe	9 countries studied out of 15 developing countries	18	5.25
		Albania	2	0.58
		Bosnia and Herzegovina	1	0.29
		Bulgaria	1	0.29
		Kosovo	1	0.29
		Poland	4	1.17
		Romania	1	0.29
		Russia	3	0.87
		Serbia	4	1.17
		Ukraine	1	0.29
**Latin America (including the Caribbean)**		13 countries studied out of 37 developing countries	62	18.08
	South America		53	15.46
		Argentina	7	2.04
		Bolivia	1	0.29
		Brazil	32	9.33
		Chile	1	0.29
		Colombia	1	0.29
		Ecuador	3	0.87
		Paraguay	1	0.29
		Peru	6	1.75
	Caribbean		2	0.58
		Haiti	1	0.29
		Trinidad and Tobago	1	0.29
	Central America		7	2.04
		Mexico	6	1.75
		Panama	1	0.29
		Did not report country name	1	0.29
**Oceania**		0 countries studied out of 10 developing countries	0	0.00

*Two continents, Antarctica and North America, are not listed as they do not have developing countries.

†The developing countries are defined based on the IMF definition.

The 341 papers employed a wide arrange of instruments to assess mental health (**Table 3**). The popular measures are GAD (50.18%), DASS (18.64%), and SAS (11.83%) for anxiety, PHQ (49.38%) and DASS (21.99%) for depression, K6 (29.93%); for distress, ISI (68.75%) and PSQI (20.31%) for insomnia, and PCL (54.55%), and IES (36.36%) for PTSD.

**Table 3:** Popular instruments measuring mental health symptoms and their cut-off points

			Primary cut-off			Cut-off variants		
**Outcome**	**Instrument**	**Outcome level**	**Lower bound**	**Upper bound**	**Reference**	**Lower bound**	**Upper bound**	**Reference**
**Anxiety**	GAD-7	Mild	5	9	[32]	4, 7	10	[33,34]
		Moderate	10	14		11	16	
		Severe	15	21		17	22	
	SAS	Mild	50	59	[35]	45	N/A	[36]
		Moderate	60	69		N/A	59	
		Severe	70	N/A		60	74	
	DASS-21	Mild	7	9	[37]	8	9	[38,39]
		Moderate	10	14		7	N/A	
		Severe	15	42		15	19	
	BAI	Mild	8	15	[40]	22	35	[41]
		Moderate	16	25		36	63	
		Severe	26	63				
	HADS	Mild	8	10	[42]	7	10	[43]
		Moderate	11	14		10	21	
		Severe	15	21				
**Depression**	PHQ-9	Mild	5	9	[44]	6	8	[45]
		Moderate	10	14		9	14	
		Severe	15	27		15	27	
	DASS-21	Mild	10	12	[46]	1, 9, 10	13	[47-49]
		Moderate	13	20		14	20	
		Severe	21	42		11	N/A	
	HADS	Mild	8	10	[42]	7	10	[43]
		Moderate	11	14		10	21	
		Severe	15	21				
	CES-D-10	Mild	9	13	[50]	10	N/A	[33,51]
		Moderate	14	N/A		11	16	
**Insomnia**	ISI	Mild	8	14	[52]	9		[53]
		Moderate	15	21		10	N/A	
		Severe	22	28				
	AIS	Mild above	6	N/A	[54]	5	N/A	[55]
	PSQI	Overall	10	N/A	[56]	8	N/A	[57]
**PTSD**	IES-R	Mild	20	N/A	[58]	9, 18, 24	N/A	[59-61]

PHQ – Generalized Anxiety Disorder scale, DASS – Depression Anxiety Stress Scale, SAS – Self-rating Anxiety Scale, HADS – Hospital Anxiety Depression Scale, BAI – Beck Anxiety Inventory, HAMA – Hamilton Anxiety Rating Scale, PHQ – Patient Health Questionnaire depression scale, CES – Center for Epidemiologic Studies Depression Scale, ISI – Insomnia Severity Index, PSQI – Pittsburgh Sleep Quality Index, AIS –Athens Insomnia Scale, PSQI – Pittsburgh Sleep Quality Index, IES – Impact of Event Scale – Revised, PTSD – Posttraumatic stress disorder

### Major issues from findings of the key study characteristics

Our analysis reveals several widespread issues in mental health research during COVID-19, such as a wide array of used instruments, inconsistent reporting of prevalence rates, inconsistent use and reporting of cut-off points, varied cut-off values for determining the overall prevalence as well as the severity, and other issues on reporting standards and terminologies. **Table 3** summarizes popular instruments used for measuring the five mental health symptoms with their primary cut-off point and different variants. Table S2 in the **Online Supplementary Document** summarizes the full list of instruments used by the individual studies included by this meta-analysis. All these issues may contribute to the heterogeneity and confusion in accumulating evidence.

**A myriad of instruments:** The individual studies on mental health research during COVID-19 employed a wide variety of instruments with varying degrees of popularity and validity, making it challenging to compare or accumulate evidence.

**Admixed outcome severity level:** The individual studies reported the prevalence rates at a range of symptom severity. First, the studies use different terminologies when reporting the overall prevalence rates. The overall prevalence rate could indicate the percentage with moderate symptoms or above, or mild symptoms or above (eg, [62]). Even worse, many studies did not specify if the overall prevalence rate used cut-off at the level of above mild or above moderate. Second, some studies use other terminologies, such as “extremely severe” [63], “very severe” [64], or “very high” [65], “moderate-severe” [66], “moderate to severe” [67,68], “moderately severe” [69], and “poor” (40), making it even more challenging to categorize symptoms. We manually recoded all the studies that indicated their cut-off scores.

**Clarity on the cut-off points used to determine severity:** Some studies employed non-standard or unusual cut-off scores [70], at times without referencing validation studies that supported the use of those special cut-off scores (eg, [50,71]). Some studies did not report the cut-off score used or did not provide any references [72,73], making the comparison and accumulation difficult. All cut-off variants of the 5 mental health symptoms are listed in **Table 3**.

### Pooled prevalence rates of mental health symptoms

**Table 4** reports the pooled prevalence rates of mental health symptoms by subgroups of population, outcome, severity, and region. The meta-analyses generally found mental health symptoms to be highly prevalent yet different across regions. Comparatively, Africa had the worst overall mental health symptoms (39%), followed by West Asia (36%) and Latin America (35%). Among different populations, medical students had the worst overall mental health symptoms (38%), followed by general students (30%) and frontline HCWs (27%). Adults suffered most from distress symptoms (29%), followed by depression (27%) and anxiety (25%). Overall, a whopping 43% of adults in developing countries suffered from mild above mental health symptoms, 21% suffered moderate above, and 8% severe mental health symptoms.

**Table 4:** The pooled prevalence rates of mental health disorders by subgroups of population, outcome, severity, and region

First-level subgroup	Second-level subgroup	Sample size (n)*	Prevalence (%)	95% CI
**Aggregated**			26%	25, 27
**Region**	**Africa**	15 391	39%	35, 44
	**Asia:**			
	East Asia	1 402 610	18%	16, 19
	South Asia	44 184	32%	28, 35
	Southeast Asia	20 914	20%	16, 24
	**West Asia**	13 289	36%	30, 42
	**Europe**	15 023	32%	26, 38
	**Latin America**	196 411	35%	32, 37
**Population**	Frontline HCWs	71 539	27%	24, 29
	General HCWs	123 698	25%	23, 27
	General population	697 481	23%	21, 25
	Adult students	796 214	30%	27, 32
	Medical students	18 890	38%	32, 44
**Outcome†**	Anxiety	1 257 838	25%	24, 27
	Depression	321 495	27%	26, 29
	Distress	76 074	29%	21, 37
	Insomnia	41 440	24%	21, 27
	PTSD	10 975	20%	13, 27
**Severity†**	Above mild	1 213 070	43%	41, 44
	Above moderate	385 010	21%	20, 23
	Above severe	158 396	8%	7, 9
	**Overall**	61 346		

CI – confidence interval, PTSD – posttraumatic stress disorder, HCW – health care worker

*The total independent samples are larger than the number of studies because some studies included multiple samples.

†The total sample sizes are larger than the total sample of the 404 independent samples because one sample can assess multiple mental health outcomes.

The results of subgroup analyses of popular instruments of mental health symptoms show the various instruments lead to different results (**Table 5**). While the prevalence rates of anxiety measured by GAD (27%) and DASS (29%) are relatively close, they are significantly different from those measured by SAS (7%), HADS (39%), and BAI (17%). The prevalence rates of depression differ significantly among studies with different measurements, specifically, PHQ (30%), DASS (26%), HADS (33%), SDS (12%), and CES (36%). The prevalence rates of distress measured by DASS (30%) and CPDI (27%) are significantly higher than those measured by K6 (18%). The prevalence rates of insomnia measured by ISI are 22%, which was significantly different from those measured by PSQI (29%) and AIS (44%). While the prevalence rates of PTSD are 15% when measured by PCL by 36% measured by IES. At least partially due to the popularity and standardized usage, the anxiety symptoms measured by GAD-7, GAD-2, and DASS-21 appear more comparable than those measured using other measurements. Similarly, the depression symptoms measured by PHQ-9 or DASS-21 and the insomnia symptoms by ISI appear to be more comparable.

**Table 5:** Instruments measuring mental health symptoms and the results of subgroup analyses on instruments*

Instrument	Frequency	Percentage	Prevalence rate (%)†
**Anxiety**	279		
GAD (GAD-7/GAD-2))	140	50.18%	27 (26, 29)
DASS–21	52	18.64%	29 (26, 33)
SAS	33	11.83%	7 (6, 9)
HADS	23	8.24%	39 (30, 48)
BAI	6	2.15%	17 (5, 34)
HAMA	4	1.43%	28 (14, 44)
**Depression**	241		
PHQ (PHQ-9/PHQ-2)	119	49.38%	30 (28, 32)
DASS-21	53	21.99%	26 (22, 30)
HADS	21	8.71%	33 (24, 42)
SDS	18	7.47%	12 (08, 16)
CES (CES-D-20/9/10)	12	4.98%	36 (26, 47)
**Distress**	27		
K–6	8	29.63%	18 (12, 26)
DASS	4	14.81%	30 (19, 43)
CPDI	4	14.81%	27 (9, 91)
**Insomnia**	64	100%	
ISI	44	68.75%	22 (19, 25)
PSQI	13	20.31%	29 (21, 38)
AIS	4	6.25%	44 (31, 58)
**PTSD**	22		
PCL	12	54.55%	15 (6, 25)
IES	8	36.36%	36 (22, 51)

PHQ – Generalized Anxiety Disorder scale, DASS – Depression Anxiety Stress Scale, SAS – Self-rating Anxiety Scale, HADS – Hospital Anxiety Depression Scale, BAI – Beck Anxiety Inventory, HAMA – Hamilton Anxiety Rating Scale, PHQ – Patient Health Questionnaire depression scale, SDS – Self-rating Depression Scale, CES – Center for Epidemiologic Studies Depression Scale, K-6 – the six items Kessler mental distress scale, CPDI – COVID-19 Peritraumatic Distress Index), ISI – Insomnia Severity Index, PSQI – Pittsburgh Sleep Quality Index, AIS –Athens Insomnia Scale, PCL – Posttraumatic Stress Disorder Checklist – Civilian Version, IES – Impact of Event Scale

*Only instruments used in at least four studies were included.

†Values are expressed with a 95% confidence interval

### Meta-regression on the prevalence of mental health symptoms

To better explain the heterogeneity of the prevalence of mental health symptoms, **Table 6** reports the results of a meta-regression analysis. The meta-analytical model explained over 51% of the variance of mental health symptoms among these studies (*R-squared* = 51.8%, *tau^2^* = 0.15). The prevalence rates of depression (*P* = 0.012) are significantly higher than those of anxiety (reference). The prevalence of severe mental health symptoms is significantly lower than that of moderate mental illness (reference) (*P* < 0.001), which is in turn significantly lower than that of mild mental illness (*P* < 0.001). The prevalence rates of general HCWs and the general population are significantly lower (*P* < 0.001) than those of frontline HCWs (reference) (*P* < 0.001).

**Table 6:** Meta-regression results on mental health symptoms during COVID-19

Variables	Coefficient*	SE	*P* value
**Outcome**			
Anxiety (reference):			
Depression	0.06 (0.01, 0.10)	0.02	0.014
Distress	0.09 (-0.04, 0.23)	0.07	0.17
Insomnia	-0.02 (-0.09, 0.05)	0.04	0.57
Posttraumatic stress disorder	0.03 (-0.10, 0.17)	0.07	0.64
**Outcome level**			
Mild above	0.46 (0.41, 0.51)	0.02	<0.001
Moderate above (reference)			
Severe	-0.42 (-0.48, -0.37)	0.03	<0.001
**Population:**			
General HCWs	-0.12 (-0.18, -0.06)	0.03	<0.001
General population	-0.11 (-0.17, 0.05)	0.03	<0.001
Frontline HCWs (reference)			
General students	0.01 (-0.07, 0.10)	0.04	0.80
Medical students	0.09 (-0.01, 0.20)	0.05	0.086
**Region:**			
Africa	0.16 (0.07, 0.25)	0.05	0.001
East Asia	-0.38 (-0.45, -0.30)	0.04	<0.001
South Asia (reference)			
Southeast Asia	-0.31 (-0.42, -0.19)	0.06	<0.001
West Asia	0.09 (-0.01, 0.19)	0.05	0.083
Europe	-0.06 (-0.17, 0.06)	0.06	0.34
Latin America	0.08 (-0.01, 0.17)	0.05	0.070
Sample size/1000000	-0.82 (-1.51, -0.13)	0.00	0.018
Quality	0.03 (0.00, 0.05)	0.01	0.064
Published vs Preprint (reference)	-0.02 (-0.12, 0.08)	0.05	0.72
Least developing vs Emerging developing (reference)	-0.12 (-0.22, -0.03)	0.05	0.012
Cross-sectional vs Cohort (reference)	0.05 (-0.05, 0.15)	0.05	0.31
Constant	1.02 (0.79, 1.25)	0.12	<0.001
R^2^	0.51		
Wald X^2^ (16)	1488		<0.001

HCWs – Healthcare workers; SE – standardized error

*Values are expressed with a 95% confidence interval.

The prevalence of mental health symptoms of African adults was significantly higher than in South Asia (reference) (*P* = 0.001), which in turn was significantly higher than in East Asiam(*P* < 0.001) and Southeast Asia (*P* < 0.001) yet not significantly different from West Asia, Europe, and Latin America (*P* > 0.05). The prevalence rates reported by studies with larger sample size are significantly lower than those of studies with smaller sample size (*P* = 0.018). The prevalence rates of mental health symptoms in emergent and other developing countries were significantly higher than those in the least developing countries (reference) (*P* = 0.012). Analyses of studies with a higher quality rating (*P* = 0.06), publication status (*P* = 0.72), and research design (*P* = 0.31) did not predict significant prevalence rates.

**Figure 2:**
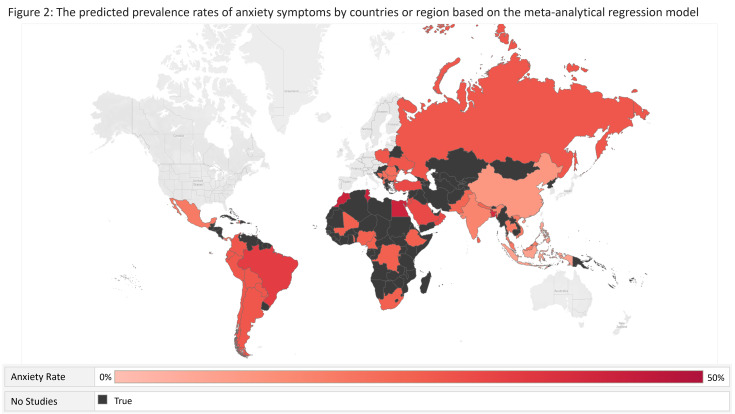
The predicted prevalence rates of depression symptoms by countries or regions, based on the meta-analytical regression model.

**Figure 3:**
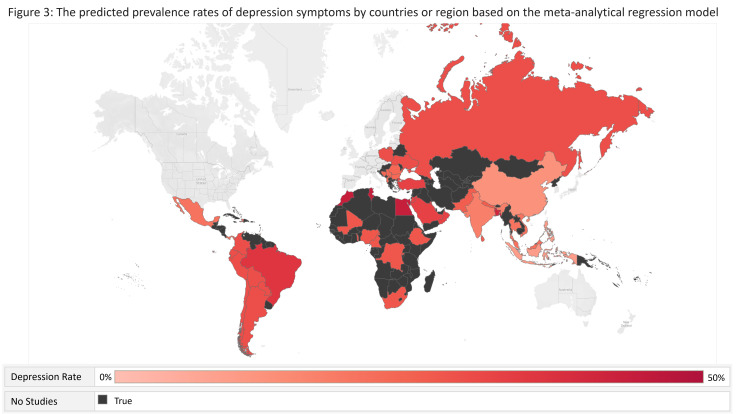
The predicted prevalence rates of anxiety symptoms by countries or regions, based on the meta-analytical regression model.

**Table 7** shows the predicted prevalence rates of mental health symptoms by populations, outcomes, severity, and regions by the meta-analytical regression model. **Figure 2** and **Figure 3** show the predicted prevalence rates of depression and anxiety symptoms in different countries or regions, respectively.

**Table 7:** The predicted prevalence rates of mental health disorders by populations, outcomes, severity, and region based on the meta-analytical regression model*

	Prevalence rate (%)†						
	**Africa**	**East Asia**	**Europe**	**Latin America**	**South Asia**	**Southeast Asia**	**West Asia**
**Frontline HCWs:**							
Sample	K = 4, n = 1259	K = 47, n = 66 208	K = 5, n = 1717	K = 2, n = 1477	K = 8, n = 2349	K = 5, n = 1081	K = 2, n = 819
Aggregated anxiety	43 (39, 47)	19 (16, 21)	32 (28, 37)	39 (35, 43)	35 (31, 39)	21 (18, 26)	39 (35, 44)
Mild above anxiety	61 (57, 65)	35 (32, 38)	51 (45, 56)	58 (53, 62)	53 (49, 58)	38 (33, 43)	58 (53, 63)
Moderate above anxiety	38 (34, 43)	15 (13, 18)	28 (24, 33)	35 (31, 39)	31 (27, 35)	18 (14, 22)	35 (31, 40)
Severe anxiety	20 (16, 23)	4 (2, 5)	12 (9, 16)	17 (14, 20)	14 (11, 17)	5 (3, 8)	17 (13, 21)
Aggregated depression	46 (41, 50)	21 (19, 23)	35 (30, 40)	42 (38, 46)	38 (34, 46)	24 (20, 28)	42 (38, 47)
Mild above depression	64 (60, 68)	38 (35, 41)	53 (48, 59)	60 (56, 64)	56 (52, 60)	41 (36, 46)	61 (56, 65)
Moderate above depression	41 (37, 46)	17 (15, 20)	31 (26, 36)	38 (34, 42)	34 (30, 38)	20 (16, 24)	38 (33, 43)
Severe depression	22 (18, 26)	5 (3, 6)	14 (10, 18)	19 (15, 22)	16 (12, 19)	6 (4, 9)	19 (15, 23)
Aggregated insomnia	42 (37, 47)	18 (15, 21)	32 (26, 37)	38 (34, 43)	34 (30, 39)	21 (16, 26)	38 (33, 44)
Mild above insomnia	60 (55, 65)	34 (30, 38)	50 (44, 56)	57 (52, 61)	52 (47, 58)	37 (32, 43)	57 (51, 62)
Moderate above insomnia	37 (33, 42)	15 (12, 17)	27 (22, 33)	34 (29, 39)	30 (25, 35)	17 (13, 22)	34 (29, 40)
Severe insomnia	19 (15, 23)	3 (2, 5)	11 (8, 15)	16 (12, 20)	13 (10, 17)	5 (2, 8)	16 (12, 21)
**General HCWs**							
Sample	K = 12, n = 3928	K = 49, n = 77 532	K = 6, n = 3445	K = 19, n = 12 821	K = 20, n = 9393	K = 9, n = 2747	K = 11, n = 6066
Aggregated anxiety	37 (33, 41)	14 (12, 16)	27 (23, 31)	33 (30, 36)	29 (26, 33)	17 (13, 20)	34 (30, 38)
Mild above anxiety	55 (51, 59)	29 (27, 32)	45 (40, 50)	52 (48, 55)	47 (43, 51)	33 (28, 37)	52 (48, 56)
Moderate above anxiety	33 (29, 37)	11 (9, 13)	23 (19, 27)	29 (26, 33)	26 (22, 29)	14 (10, 17)	30 (26, 34)
Severe anxiety	15 (12, 18)	2 (1, 3)	8 (6, 11)	12 (10, 15)	10 (7, 12)	3 (1, 5)	13 (10, 16)
Aggregated depression	4 (36, 43)	16 (14, 18)	29 (25, 34)	36 (33, 39)	32 (28, 36)	19 (15, 23)	36 (32, 40)
Mild above depression	58 (54, 62)	32 (29, 34)	47 (42, 52)	54 (51, 58)	50 (46, 54)	35 (31, 40)	55 (50, 59)
Moderate above depression	35 (31, 39)	13 (11, 15)	25 (21, 30)	32 (28, 35)	28 (24, 32)	15 (12, 19)	32 (28, 36)
Severe depression	17 (14, 20)	2 (2, 4)	10 (7, 13)	14 (12, 17)	12 (9, 14)	4 (2, 6)	15 (11, 18)
Aggregated insomnia	36 (32, 40)	13 (11, 16)	26 (21, 31)	32 (29, 36)	29 (24, 33)	16 (12, 20)	33 (28, 37)
Mild above insomnia	54 (50, 59)	28 (25, 32)	44 (38, 49)	51 (46, 55)	46 (42, 51)	32 (27, 37)	51 (46, 56)
Moderate above insomnia	32 (27, 36)	11 (8, 13)	22 (18, 27)	28 (24, 32)	25 (21, 29)	13 (9, 17)	29 (24, 33)
Severe insomnia	14 (11, 18)	1 (1, 3)	8 (5, 11)	12 (9, 15)	9 (6, 12)	2 (1, 4)	12 (9, 16)
**General population:**							
Sample	K = 15, n = 7245	K = 70, n = 484 401	K = 7, n = 6249	K = 35, n = 170 137	K = 9, n = 14 950	K = 8, n = 14 348	K = 1, n = 1798
Aggregated anxiety	37 (34, 41)	15 (13, 16)	28 (23, 32)	34 (31, 37)	30 (26, 34)	17 (14, 21)	34 (30, 39)
Mild above anxiety	56 (52, 60)	30 (27, 32)	45 (40, 50)	52 (49, 56)	48 (44, 52)	33 (29, 38)	53 (48, 57)
Moderate above anxiety	33 (29, 37)	12 (10, 13)	24 (20, 28)	30 (27, 33)	26 (23, 30)	14 (11, 18)	30 (26, 34)
Severe anxiety	15 (12, 19)	2 (1, 3)	9 (6, 12)	13 (11, 15)	10 (8, 13)	3 (1, 5)	13 (10, 16)
Aggregated depression	4 (36, 44)	17 (15, 19)	3 (26, 35)	37 (34, 40)	33 (29, 36)	9 (16, 23)	37 (33, 41)
Mild above depression	59 (55, 63)	32 (30, 35)	48 (43, 53)	55 (52, 58)	51 (47, 55)	36 (31, 41)	55 (51, 60)
Moderate above depression	36 (32, 40)	13 (12, 15)	26 (22, 31)	32 (29, 36)	29 (25, 32)	16 (13, 20)	33 (28, 37)
Severe depression	17 (14, 21)	3 (2, 4)	10 (7, 14)	15 (12, 17)	12 (9, 15)	4 (2, 6)	15 (12, 19)
Aggregated insomnia	37 (32, 41)	14 (12, 16)	27 (22, 32)	33 (29, 37)	29 (25, 34)	16 (12, 21)	33 (28, 38)
Mild above insomnia	55 (50, 60)	29 (26, 32)	44 (39, 50)	51 (47, 56)	47 (42, 52)	32 (27, 38)	52 (46, 57)
Moderate above insomnia	32 (28, 37)	11 (9, 13)	23 (18, 28)	29 (25, 33)	25 (21, 30)	13 (10, 17)	29 (24, 34)
Severe insomnia	15 (11, 18)	2 (1, 3)	8 (5, 12)	12 (9, 15)	10 (7, 13)	3 (1, 5)	12 (9, 16)
**General students:**							
Sample	K = 0, n = 0	K = 15, n = 769 771	K = 4, n = 3612	K = 9, n = 12 523	K = 7, n = 4598	K = 3, n = 2738	K = 5, n = 2637
Aggregated anxiety		19 (16, 22)	33 (28, 38)	4 (36, 44)	36 (31, 40)	22 (18, 26)	4 (35, 45)
Mild above anxiety		35 (32, 39)	51 (46, 57)	58 (54, 62)	54 (49, 59)	39 (34, 44)	58 (53, 63)
Moderate above anxiety		16 (13, 19)	29 (24, 34)	35 (31, 40)	31 (27, 36)	18 (14, 23)	36 (31, 41)
Severe anxiety		4 (2, 6)	12 (9, 16)	17 (14, 21)	14 (11, 18)	5 (3, 8)	17 (14, 21)
Aggregated depression		21 (18, 25)	36 (30, 41)	42 (38, 47)	38 (34, 43)	24 (20, 29)	43 (38, 48)
Mild above depression		38 (34, 42)	54 (48, 60)	61 (56, 65)	57 (52, 62)	42 (36, 47)	61 (56, 66)
Moderate above depression		18 (15, 21)	31 (26, 37)	38 (34, 43)	34 (29, 39)	21 (16, 25)	38 (33, 43)
Severe depression		5 (3, 7)	14 (10, 18)	19 (16, 23)	16 (12, 20)	7 (4, 10)	19 (15, 24)
Aggregated insomnia		18 (15, 22)	32 (26, 38)	39 (34, 44)	35 (29, 40)	21 (16, 26)	39 (33, 45)
Mild above insomnia		34 (30, 39)	50 (44, 57)	57 (52, 62)	53 (47, 59)	38 (32, 44)	57 (52, 63)
Moderate above insomnia		15 (12, 19)	28 (22, 34)	34 (29, 40)	31 (25, 36)	18 (13, 23)	35 (29, 40)
Severe insomnia		3 (2, 6)	12 (8, 16)	16 (12, 21)	13 (10, 18)	5 (2, 8)	17 (12, 21)

K – number of samples, n – number of participants, HCW – health care worker

*Due to the small number of samples on distress, posttraumatic stress disorder (PTSD), and medical students, it is not meaningful to report the prevalence rates by different regions.

†All values are expressed with a 95% confidence interval.

### Sensitivity analysis

Our meta-analytical model considered several factors, such as publication status (insignificant), sample size (significant), and article quality score (insignificant). Furthermore, excluding each study one by one from the meta-analytical model did not significantly alter the findings. A visual inspection of the sensitivity plot, however, revealed that there is significant asymmetry. Figure S2 in the **Online Supplementary Document** reports the DOI plot in combination with the Luis-Kanamori (LFK) index, which has higher sensitivity and power than a funnel plot [74,75]. LFK index scores of ±1, between ±1 and ±2, or ±2 indicate “no asymmetry”, “minor asymmetry”, and “major asymmetry” respectively, and hence the LFK index of 5.61 represents major asymmetry. Therefore, the presence of publication bias is likely for mental health prevalence studies under COVID-19.

## DISCUSSION

By systematically reviewing studies on the prevalence rates of mental health symptoms in developing countries worldwide, we were able to identify and reveal the uneven number of studies on mental health symptoms across countries to highlight the need to initiate relevant studies on the neglected countries. The meta-regression results provide better evidence on the prevalence rates of anxiety, depression, and insomnia in developing countries across regions and based on the level of development.

### Uneven research efforts across regions

This study found that the research effort on mental health during COVID-19 has been highly uneven across countries, regions, and mental health outcomes. The studies are far from evenly distributed across countries: there have been 147 studies on China (42.9%), 32 on Brazil (9.3%), 24 on India (7.0%), 10 on Bangladesh (2.9%) and Malaysia (2.9%), yet only 40 out of the 167 developing countries have been studied, leaving 127 developing countries without a single study. Granted, China, India, and Brazil are huge countries, and China was the first to experience the COVID-19 crisis, so a high number of studies in those countries is commended. Still, there have been no studies in 45 out of 56 countries in Africa (80.4%), 32 out of 49 countries in Asia (65.3%), 24 out of 37 countries in Latin America (64.9%), and 6 out of 15 countries in Europe (40%). The highly uneven distribution of research efforts calls for research attention on the unstudied countries. Given the lack of studies in many countries at the country level, the evidence from our meta-analysis may help by giving at least the mental health prevalence from nearby countries or regions, to enable health care organizations who need data for evidence-based decision making.

### Meta-regression findings

Thanks to a large number of samples in developing countries overall, we were able to conduct meta-regression to account for multiple predictors at the same time to enable better prediction of the prevalence of each mental health symptom. The accumulated evidence shows that several predictors are significantly associated with prevalence rates of mental symptoms during COVID-19, including the severity and type of mental symptoms, population, region, sample size, and study characteristics.

The severity of mental symptoms, largely unaccounted for in prior meta-analyses, was found to contribute greatly to the heterogeneity of prevalence rates; hence, future research on mental health needs to break down and pay special attention to the severity and specify its level. The significant differences revealed by this study call for more meta-analyses on varying levels of severity to provide evidence for practitioners relevant to their concerns.

Among the mental health symptoms examined, distress and depression generally had the highest prevalence rates. Our findings suggest that practitioners need to pay more attention to distress and depression of various populations under the COVID-19 pandemic [76-78].

While not significantly higher than frontline HCWs, general adult students and medical students suffered more than general HCWs and the general population. More than a two-third of studies investigated general HCWs and the general population to generate more meta-analytical evidence, which suggests that policymakers and health care organizations need to further prioritize frontline HCWs and students in this ongoing pandemic. Medical (including nursing) students [79] are worthy of special attention.

### A mental health research agenda during COVID-19

Our systemic review and meta-analysis uncovers several widespread problems in the individual papers that impede evidence accumulation. We offer a few concrete suggestions for focusing research and reporting future mental health studies for authors, editors, and reviewers (**Table 8**), to improve the quality of mental health studies and to facilitate evidence accumulation in future meta-analyses. To make results consistent and comparable, we strongly suggest researchers to use standardized scales with well-established cut-off points (see **Table 3** for the popular instruments to measure mental health symptoms and their cut-off points and **Table 5** for the results of subgroup analyses on instruments).

**Table 8:** A list of recommendations for conducting and reporting future mental health research studies

Outcome and instrument	1) Study health outcomes that have higher prevalence rates, eg, distress
2) Use the well-established instruments with the standard cut-off points listed in **Table 3**.
**Severity of the symptoms**	3) Report more levels of severity of symptoms and the cut-off points used
	4) Specify the meaning of overall prevalence, whether above mild or above moderate
	5) Specify the cut-off values used with the reasons/references
**Characteristics of the samples**	6) Report the sampling dates
	7) Report the age/gender of the participants
	8) Report participant rate
**Population**	9) Separate and focus on frontline HCWs from general HCWs
	10) Separate and focus on general adult students and medical students
**Study design**	11) More future research using cohort designs

### Study limitations and future research

This meta-analysis has a few limitations. First, there may be some bias because all studies were English. Second, 96.48% of studies included in this meta-analysis were cross-sectional surveys, and we call for more cohort studies to examine the effect over time [80]. Third, the validity of our findings rests upon the quality and reporting of the original studies. As discussed before, individual mental health studies varied in their usage of instruments, cut-off scores, the use of cut-off scores to define mental symptoms, and the reporting standards. For example, the overall prevalence refers to “above the cut-off of mild” in some studies, yet “above the cut-off of moderate” in others. Worse, many studies report the overall prevalence without specifying which/how cut-off scores are used. While we focused on the severity, the cut-off points, and the ways in which individual studies used this information, various approaches contribute to additional noise and variance in the analysis. It is also possible that the diagnostic systems might need to be adjusted across contexts, but such adjustments need to be carefully validated and reported. Fourth, we are limited in examining linear effect, and future research may examine nonlinear effect, as past research has shown age and distance to epicentre may have nonlinear effect on mental health [8,81,82]. Lastly, various classification schemes and terminologies exist on “developing countries” exist, such as low- and middle-income countries (LMIC), newly industrialized countries, emerging markets, third world countries, etc. and future research may use our data to analyse based on other classification schemes.

## CONCLUSION

Since the COVID-19 pandemic started in November 2019, hundreds of studies have documented the mental health of major populations by the key mental outcomes and varying levels of severity across the world. This systematic review and meta-analysis synthesized the evidence on the prevalence rates of mental health symptoms in developing countries under the COVID-19 pandemic. We hope this meta-analysis reveals and synthesizes not only the accumulative evidence on mental health research but also reveals key directions for this important research stream.

## Additional material


Online Supplementary Document

## References

[1] De Sousa A Mohandas E Javed A Psychological interventions during COVID-19: challenges for low and middle income countries. Asian J Psychiatr. 2020;51:102128. 10.1016/j.ajp.2020.102128 32380441 PMC7195042

[2] International A. South Asia: As COVID-19 spreads, fears rise for people at higher risk. London: Amnesty International; 2020.

[3] Robiner WN The mental health professions: Workforce supply and demand, issues, and challenges. Clin Psychol Rev. 2006;26:600-25. 10.1016/j.cpr.2006.05.002 16820252

[4] Chen X Zhang SX Jahanshahi AA Alvarez-Risco A Dai H Li J Belief in Conspiracy Theory about COVID-19 Predicts Mental Health and Well-being – A Study of Healthcare Staff in Ecuador. JMIR Public Health Surveill. 2020;6:e20737. 10.2196/20737 32658859 PMC7375774

[5] Mia MA Griffiths M Can South Asian Countries Cope with the Mental Health Crisis Associated with COVID-19? Int J Ment Health Addict. 2021;16:1. 10.1007/s11469-021-00491-5 33613132 PMC7886190

[6] Sameer AS Khan MA Nissar S Banday MZ Assessment of Mental Health and Various Coping Strategies among general population living Under Imposed COVID-Lockdown Across world: A Cross-Sectional Study. Ethics Med Public Health. 2020;15:100571. 10.1016/j.jemep.2020.100571 32838000 PMC7386294

[7] Holmes EA O’Connor RC Perry VH Tracey I Wessely S Arseneault L Multidisciplinary research priorities for the COVID-19 pandemic: a call for action for mental health science. Lancet Psychiatry. 2020;7:547-60. 10.1016/S2215-0366(20)30168-1 32304649 PMC7159850

[8] Lateef T Chen J Tahir M Lateef TA Chen BZ Li J Typhoon Eye Effect versus Ripple Effect: The Role of Family Size on Mental Health during the COVID-19 Pandemic in Pakistan. Global Health. 2021;17:32. 10.1186/s12992-021-00685-5 33781286 PMC8006139

[9] Zhang SX Chen J Scientific evidence on mental health in key regions under the COVID-19 pandemic - Meta-analytical evidence from Africa, Asia, China, Eastern Europe, Latin America, South Asia, Southeast Asia, and Spain. Eur J Psychotraumatol. In press. 10.1080/20008198.2021.2001192 34900123 PMC8654399

[10] Chen J Farah N Dong RK Chen RZ Xu W Yin A The Mental Health Under the COVID-19 Crisis in Africa: A Systematic Review and Meta-Analysis. Int J Environ Res Public Health. In press. 10.1101/2021.04.19.21255755 PMC8536091 34682357

[11] International Monetary Fund. World Economic and Financial Surveys World Economic Outlook Database—WEO Groups and Aggregates Information 2018. Washington DC: IMF; 2018.

[12] Salazar de Pablo G Vaquerizo-Serrano J Catalan A Arango C Moreno C Ferre F Impact of coronavirus syndromes on physical and mental health of health care workers: Systematic review and meta-analysis. J Affect Disord. 2020;275:48-57. 10.1016/j.jad.2020.06.022 32658823 PMC7314697

[13] Usher K Jackson D Durkin J Gyamfi N Bhullar N Pandemic-related behaviours and psychological outcomes; A rapid literature review to explain COVID-19 behaviours. Int J Ment Health Nurs. 2020;29:1018-34. 10.1111/inm.12790 32860475

[14] Hong QN Fàbregues S Bartlett G Boardman F Cargo M Dagenais P The Mixed Methods Appraisal Tool (MMAT) version 2018 for information professionals and researchers. Educ Inf. 2018;34:285-91. 10.3233/EFI-180221

[15] Michael Borenstein LVH, Julian PT. Higgins, Hannah R. Rothstein. Introduction to meta-analysis. Oxford: John Wiley & Sons; 2021.

[16] Borenstein M Higgins JPT Hedges LV Rothstein HR Basics of meta-analysis: I2 is not an absolute measure of heterogeneity. Res Synth Methods. 2017;8:5-18. 10.1002/jrsm.1230 28058794

[17] Pappa S Ntella V Giannakas T Giannakoulis VG Papoutsi E Katsaounou P Prevalence of depression, anxiety, and insomnia among healthcare workers during the COVID-19 pandemic: A systematic review and meta-analysis. Brain Behav Immun. 2020;88:901-7. 10.1016/j.bbi.2020.05.026 32437915 PMC7206431

[18] Pappa S Chen J Barnet J Zhang A Dong RK Xu W A Systematic Review and Meta-Analysis of the Mental Health Impact of the Covid-19 Pandemic in Southeast Asia. Psychiatry Clin Neurosci. In press. 10.1111/pcn.13306 PMC8661667 34704305

[19] Furuya-Kanamori L Barendregt JJ Doi SAR A new improved graphical and quantitative method for detecting bias in meta-analysis. Int J Evid-Based Healthc. 2018;16:195-203. 10.1097/XEB.0000000000000141 29621038

[20] Yitayih Y Mekonen S Zeynudin A Mengistie E Ambelu A Mental health of healthcare professionals during the early stage of the COVID-19 pandemic in Ethiopia. BJPsych Open. 2020;7 e1:e1-6. 33256883 10.1192/bjo.2020.130 PMC7844150

[21] Kounou KB Guédénon KM Foli AAD Gnassounou-Akpa E Mental health of medical professionals during the covid-19 pandemic in Togo. Psychiatry Clin Neurosci. 2020;74:559-60. 10.1111/pcn.13108 32621390 PMC7361459

[22] Chen RZ Zhang SX Xu W Yin A Dong RK Chen BZ A Systematic Review and Meta-Analysis on Mental Illness Symptoms in Spain in the COVID-19 Crisis. Medrxiv. 2021. 10.1101/2021.04.11.21255274

[23] Chen X Chen J Zhang M Chen RZ Dong RK Dong Z One Year of Evidence on Mental Health in China in the COVID-19 Crisis - A Systematic Review and Meta-Analysis. Medrxiv. 2021. 10.1101/2021.02.01.21250929

[24] Hossain MM Rahman M Trisha NF Tasnim S Nuzhath T Hasan NT Prevalence of anxiety and depression in South Asia during COVID-19: A systematic review and meta-analysis. Heliyon. 2021;7:e06677. 10.1016/j.heliyon.2021.e06677 33898819 PMC8056240

[25] Zhang SX Miller SO Xu W Yin A Chen BZ Delios A Meta-Analytic Evidence of Depression and Anxiety in Eastern Europe during the COVID-19 Pandemic. Eur J Psychotraumatol. In press. 10.1080/20008198.2021.2000132 35186214 PMC8856103

[26] Zhang SX Batra K Liu T Dong RK Xu W Yin A Meta-Analytical Evidence on Mental Disorder Symptoms During the COVID-19 Pandemic in Latin America. Epidemiol Psychiatr Sci. In press. 10.2139/ssrn.3858820 PMC9069590 35438066

[27] Singh RK Bajpai R Kaswan P COVID-19 pandemic and psychological wellbeing among health care workers and general population: A systematic-review and meta-analysis of the current evidence from India. Clin Epidemiol Glob Health. 2021;43:100737. 10.1016/j.cegh.2021.100737 33898866 PMC8055546

[28] El-Qushayri AE Dahy A Reda A Mahmoud M Mageed SA Kamel A A closer look to the high burden of the psychiatric disorders among health care workers (HCWs) in Egypt during COVID-19 outbreak: A meta-analysis of 3137 HCWs. Epidemiol Health. 2021;2021:e2021045. 10.4178/epih.e2021045 34265893 PMC8602011

[29] Norhayati MN RC and Azman, MY. Prevalence of Psychological Impacts on Healthcare Providers during COVID-19 Pandemic in Asia. Int J Environ Res Public Health. 2021;18:9157. 10.3390/ijerph18179157 34501747 PMC8431592

[30] Cénat JM Dalexis RD Guerrier M Noorishad P-G Derivois D Bukaka J Frequency and correlates of anxiety symptoms during the COVID-19 pandemic in low-and middle-income countries: A multinational study. J Psychiatr Res. 2021;132:13-7. 10.1016/j.jpsychires.2020.09.031 33035760 PMC7527178

[31] Chew NW Ngiam J Tan B Tham SM Tan CYS Jing M Asian-Pacific perspective on the psychological well-being of healthcare workers during the evolution of the COVID-19 pandemic. BJPsych Open. 2020;6:e116. 10.1192/bjo.2020.98 33028449 PMC7542327

[32] Ni Z Lebowitz ER Zou Z Wang H Liu H Shrestha R Response to the COVID-19 Outbreak in Urban Settings in China. J Urban Health. 2021;98:41-52. 10.21203/rs.3.rs-71833/v1 33258088 PMC7703725

[33] Nisha SN Francis YM Balaji K Raghunath G Kumaresan M A survey on anxiety and depression level among South Indian medical students during the COVID 19 pandemic. International Journal of Research in Pharmaceutical Sciences. 2020;11:779-86. 10.26452/ijrps.v11iSPL1.3082

[34] Wańkowicz P Szylińska A Rotter I Assessment of Mental Health Factors among Health Professionals Depending on Their Contact with COVID-19 Patients. Int J Environ Res Public Health. 2020;17:5849. 10.3390/ijerph17165849 32806699 PMC7459704

[35] Huang L Lei W Xu F Liu H Yu L Emotional responses and coping strategies in nurses and nursing students during Covid-19 outbreak: A comparative study. PLoS One. 2020;15:e0237303. 10.1371/journal.pone.0237303 32764825 PMC7413410

[36] Kamaludin K Chinna K Sundarasen S Khoshaim HB Nurunnabi M Baloch GM Coping with COVID-19 and movement control order (MCO): experiences of university students in Malaysia. Heliyon. 2020;6:e05339. 10.1016/j.heliyon.2020.e05339 33134570 PMC7584419

[37] Margetić B Peraica T Stojanović K Ivanec D Predictors of emotional distress during the COVID-19 pandemic; a Croatian study. Pers Individ Dif. 2021;175:110691. 10.1016/j.paid.2021.110691 33518867 PMC7837615

[38] Souza ASR Souza GFA Souza GA Cordeiro ALN Praciano GAF Alves ACS Factors associated with stress, anxiety, and depression during social distancing in Brazil. Rev Saude Publica. 2021;55:5. 10.11606/s1518-8787.2021055003152 33852675 PMC8011840

[39] Ferreira FO Lopes-Silva JB Siquara GM Manfroi EC de Freitas PM Coping in the Covid-19 pandemia: how different resources and strategies can be risk or protective factors to mental health in the Brazilian nopulation. Health Psychol Behav Med. 2021;9:182-205. 10.1080/21642850.2021.1897595 34104556 PMC8158238

[40] Landaeta-Díaz L González-Medina G Agüero SD Anxiety, anhedonia and food consumption during the COVID-19 quarantine in Chile. Appetite. 2021;164:105259. 10.1016/j.appet.2021.105259 33857546 PMC8050603

[41] Markovic I, Nikolovski S, Milojevic S, Zivkovic D, Knezevic S, Mitrovic A, et al. Public trust and media influence on anxiety and depression levels among skilled workers during the COVID-19 outbreak in Serbia. Vojnosanitetski pregled. 2020;77(11):1201-9.

[42] Wu M Han H Lin T Chen M Wu J Du X Prevalence and risk factors of mental distress in China during the outbreak of COVID-19: A national cross-sectional survey. Brain Behav. 2020;10:e01818. 10.1002/brb3.1818 32869541 PMC7667324

[43] Karpenko OA Syunyakov TS Kulygina MA Pavlichenko AV Chetkina AS Andrushchenko AV Impact of COVID-19 pandemic on anxiety, depression and distress – online survey results amid the pandemic in Russia. Consortium Psychiatricum. 2020;1:8-20. 10.17650/2712-7672-2020-1-1-8-20 PMC11047270 38680383

[44] Das A. MD, Sil A, MBBS, Jaiswal S, DNB, et al. A Study to Evaluate Depression and Perceived Stress Among Frontline Indian Doctors Combating the COVID-19 Pandemic. Cranio. 2020. 10.4088/PCC.20m02716 33031651

[45] Torrente F Yoris A Low D Lopez P Bekinschtein P Vázquez G Emotional symptoms, mental fatigue and behavioral adherence after 72 continuous days of strict lockdown during the COVID-19 pandemic in Argentina. medRxiv. 2021:21255866. 10.1101/2021.04.21.21255866 PMC8668400 34931146

[46] Chakraborty K Psychological impact of COVID-19 pandemic on general population in West Bengal: A cross-sectional study. Indian J Psychiatry. 2020;62:266-72. 32773869 10.4103/psychiatry.IndianJPsychiatry_276_20 PMC7368440

[47] Chew NWS Lee GKH Tan BYQ Jing M Goh Y Ngiam NJH A multinational, multicentre study on the psychological outcomes and associated physical symptoms amongst healthcare workers during COVID- 19 outbreak. Brain Behav Immun. 2020 b;88:559-65. 10.1016/j.bbi.2020.04.049 32330593 PMC7172854

[48] Ahmed O Ahmed MZ Alim SMAHM Khan MDAU Jobe MC COVID-19 outbreak in Bangladesh and associated psychological problems: An online survey. Death Stud. 2022;46:1080-9. 32915701 10.1080/07481187.2020.1818884

[49] Nayak BS Sahu PK Ramsaroop K Maharaj S Mootoo W Khan S Prevalence and factors associated with depression, anxiety and stress among healthcare workers of Trinidad and Tobago during COVID-19 pandemic: A cross-sectional study. BMJ Open. 2021;11:e044397. 10.1136/bmjopen-2020-044397 33849850 PMC8050873

[50] Song L Wang Y Li Z Yang Y Li HJIjoer, health p. Mental health and work attitudes among people resuming work during the Covid-19 pandemic: A cross-sectional study in China. Int J Environ Res Public Health. 2020;17:5059. 10.3390/ijerph17145059 PMC7400483 32674361

[51] Usher K, Bhullar N, Durkin J, Gyamfi N, Jackson D. Family violence and COVID-19: Increased vulnerability and reduced options for support. Wiley Online Library; 2020. 10.1111/inm.12735 PMC7264607 32314526

[52] Zhang H Shi Y Jing P Zhan P Fang Y Wang FJPr. Posttraumatic stress disorder symptoms in healthcare workers after the peak of the COVID-19 outbreak: A survey of a large tertiary care hospital in Wuhan. Psychiatry Res. 2020;294:113541. 10.1016/j.psychres.2020.113541 33128999 PMC7585629

[53] Zhang WR Wang K Yin L Zhao WF Xue Q Peng M Mental Health and Psychosocial Problems of Medical Health Workers during the COVID-19 Epidemic in China. Psychother Psychosom. 2020;89:242-50. 10.1159/000507639 32272480 PMC7206349

[54] Li X Yu H Bian G Hu Z Liu X Zhou Q Prevalence, risk factors, and clinical correlates of insomnia in volunteer and at home medical staff during the COVID-19. Brain Behav Immun. 2020;87:140-1. 10.1016/j.bbi.2020.05.008 32380272 PMC7198418

[55] Fu W Wang C Zou L Guo Y Lu Z Yan S Psychological health, sleep quality, and coping styles to stress facing the COVID-19 in Wuhan, China. Transl Psychiatry. 2020;10:255. 10.1038/s41398-020-00913-3 32647160 PMC7347261

[56] Wang W Song W Xia Z He Y Tang L Hou J Sleep disturbance and psychological profiles of medical staff and non-medical staff during the early outbreak of COVID-19 in Hubei Province, China. Front Psychiatry. 2020;11:733. 32793014 10.3389/fpsyt.2020.00733 PMC7387679

[57] Wang J Gong Y Chen Z Wu J Feng J Yan S Sleep disturbances among Chinese residents during the Coronavirus Disease 2019 outbreak and associated factors. Sleep Med. 2020;74:199-203. 10.1016/j.sleep.2020.08.002 32861011 PMC7411535

[58] Chen B, Li QX, Zhang H, Zhu JY, Yang X, Wu YH, et al. The psychological impact of COVID-19 outbreak on medical staff and the general public. J Curr Psychol. 2020:1-9. 10.1007/s12144-020-01109-0 PMC7539749 33046955

[59] Tan W Hao F McIntyre RS Jiang L Jiang X Zhang L Is returning to work during the COVID-19 pandemic stressful? A study on immediate mental health status and psychoneuroimmunity prevention measures of Chinese workforce. Brain Behav Immun. 2020;87:84-92. 10.1016/j.bbi.2020.04.055 32335200 PMC7179503

[60] Li X Li S Xiang M Fang Y Qian K Xu J The prevalence and risk factors of PTSD symptoms among medical assistance workers during the COVID-19 pandemic. J Psychosom Res. 2020;139:110270. 10.1016/j.jpsychores.2020.110270 33070044 PMC7536549

[61] Zhang C Yang L Liu S Ma S Wang Y Cai Z Survey of Insomnia and Related Social Psychological Factors Among Medical Staff Involved in the 2019 Novel Coronavirus Disease Outbreak. Front Psychiatry. 2020;11:306. 10.3389/fpsyt.2020.00306 32346373 PMC7171048

[62] Du J Dong L Wang T Yuan C Fu R Zhang L Psychological symptoms among frontline healthcare workers during COVID-19 outbreak in Wuhan. Gen Hosp Psychiatry. 2020;67:144-5. 10.1016/j.genhosppsych.2020.03.011 32381270 PMC7194721

[63] Ozamiz-Etxebarria N Dosil-Santamaria M Picaza-Gorrochategui M Idoiaga-Mondragon N Stress, anxiety, and depression levels in the initial stage of the COVID-19 outbreak in a population sample in the northern Spain. Cad Saude Publica. 2020;36:e00054020. 10.1590/0102-311x00054020 32374806

[64] Moghanibashi-Mansourieh A Assessing the anxiety level of Iranian general population during COVID-19 outbreak. Asian J Psychiatr. 2020;51:102076. 10.1016/j.ajp.2020.102076 32334409 PMC7165107

[65] Temsah M-H Al-Sohime F Alamro N Al-Eyadhy A Al-Hasan K Jamal A Somily, A. M. The psychological impact of COVID-19 pandemic on health care workers in a MERS-CoV endemic country. J Infect Public Health. 2020;13:877-82. 10.1016/j.jiph.2020.05.021 32505461 PMC7256548

[66] Guiroy A Gagliardi M Coombes N Landriel F Zanardi C Camino Willhuber G COVID-19 Impact Among Spine Surgeons in Latin America. Global Spine J. 2021;11:859. 32875914 10.1177/2192568220928032 PMC8258821

[67] Wang H Huang D Huang H Zhang J Guo L Liu Y The psychological impact of COVID-19 pandemic on medical staff in Guangdong, China: a cross-sectional study. Psychol Med. 2022;52:884-92. 32624037 10.1017/S0033291720002561 PMC7371926

[68] Moccia L Janiri D Pepe M Dattoli L Molinaro M De Martin V Affective temperament, attachment style, and the psychological impact of the COVID-19 outbreak: an early report on the Italian general population. Brain Behav Immun. 2020;87:75-9. 10.1016/j.bbi.2020.04.048 32325098 PMC7169930

[69] Xiaoming X Ming A Su H Wo W Jianmei C Qi Z The psychological status of 8817 hospital workers during COVID-19 Epidemic: A cross-sectional study in Chongqing. J Affect Disord. 2020;276:555-61. 10.1016/j.jad.2020.07.092 32871686 PMC7369013

[70] Elhai JD, Yang H, McKay D, Asmundson GJJJoAD. COVID-19 anxiety symptoms associated with problematic smartphone use severity in Chinese adults. 2020. 10.1016/j.jad.2020.05.080 PMC7251360 32663990

[71] Cai Z Cui Q Liu Z Li J Gong X Liu J Nurses endured high risks of psychological problems under the epidemic of COVID-19 in a longitudinal study in Wuhan China. J Psychiatr Res. 2020;131:132-7. 10.1016/j.jpsychires.2020.09.007 32971356 PMC7489269

[72] Cao J Wei J Zhu H Duan Y Geng W Hong X A Study of Basic Needs and Psychological Wellbeing of Medical Workers in the Fever Clinic of a Tertiary General Hospital in Beijing during the COVID-19 Outbreak. Psychother Psychosom. 2020;89:252-4. 10.1159/000507453 32224612 PMC7179543

[73] Sun Q Lu N Social Capital and Mental Health among Older Adults Living in Urban China in the Context of COVID-19 Pandemic. Int J Environ Res Public Health. 2020;17:7947. 10.3390/ijerph17217947 33138131 PMC7663485

[74] Mboua CP Keubo FRN Fouaka SGNJLEP Anxiety and Depression Associated with the Management of COVID-19 Among Healthcare Personnel in Cameroon. Evol Psychiatr (Paris). 2021;86:131-9. 10.1016/j.evopsy.2020.11.002 33318714 PMC7724313

[75] Teshome A Glagn M Shegaze M Tekabe B Getie A Assefa G Generalized Anxiety Disorder and Its Associated Factors Among Health Care Workers Fighting COVID-19 in Southern Ethiopia. Psychol Res Behav Manag. 2020;13:907. 10.2147/PRBM.S282822 33177897 PMC7652566

[76] Gong H Zhang SX Nawaser K Jahanshahi AA Xu X Li J The Mental Health of Healthcare Staff Working During the COVID-19 Crisis: Their Working Hours as a Boundary Condition. J Multidiscip Healthc. 2021;14:1073. 10.2147/JMDH.S297503 34007182 PMC8122682

[77] Yan J Kim S Zhang SX Foo MD Alvarez-Risco A Del-Aguila-Arcentales S Hospitality workers’ COVID-19 risk perception and depression: A contingent model based on transactional theory of stress model. Int J Hospit Manag. 2021;95:102935. 10.1016/j.ijhm.2021.102935 PMC9756832 36540684

[78] Zhang SX Huang H Li J Antonelli-Ponti M Paiva SFD da Silva JA Predictors of Depression and Anxiety Symptoms in Brazil during COVID-19. Int J Environ Res Public Health. 2021;18:7026. 10.3390/ijerph18137026 34209311 PMC8297012

[79] Wang S Li LZ van Antwerpen N Suparman S Gayatri M Sari NP Hand Hygiene and Mask-Wearing Practices during COVID-19 among Healthcare Workers: Misinformation as a Predictor. Am J Trop Med Hyg. 2021;105:1483. 10.4269/ajtmh.21-0463 34678760 PMC8641349

[80] Li L Zhang SX Graf-Vlachy L Predicting Managers’ Mental Health across Countries: Using Country-level COVID-19 Statistics. Front Public Health. In press. 10.1101/2021.07.18.21260567 PMC9160832 35664112

[81] Jiyao C Zhang SX Wang Y Jahanshahi AA Dinani MM Madavan AN The relationship between age and mental health among adults in Iran during the COVID-19 pandemic. Int J Ment Health Addict. 2021;1-16. 10.1007/s11469-021-00571-6 34177394 PMC8218786

[82] Zhang SX Huang H Wei F Geographical Distance to the Epicenter of Covid-19 Predicts the Burnout of Working Population: Ripple effect or Typhoon Eye effect? Psychiatry Res. 2020;288:112998. 10.1016/j.psychres.2020.112998 32325386 PMC7194871

