# Supplementary material for: Mental health symptoms during the COVID-19 pandemic in developing countries: A systematic review and meta-analysis
Source: J Glob Health. 2022 May 23;12:05011. doi: 10.7189/jogh.12.05011 (PMC9126304; doi:10.7189/jogh.12.05011)
Supplement: Online Supplementary Document [file jogh-12-05011-s001.pdf]

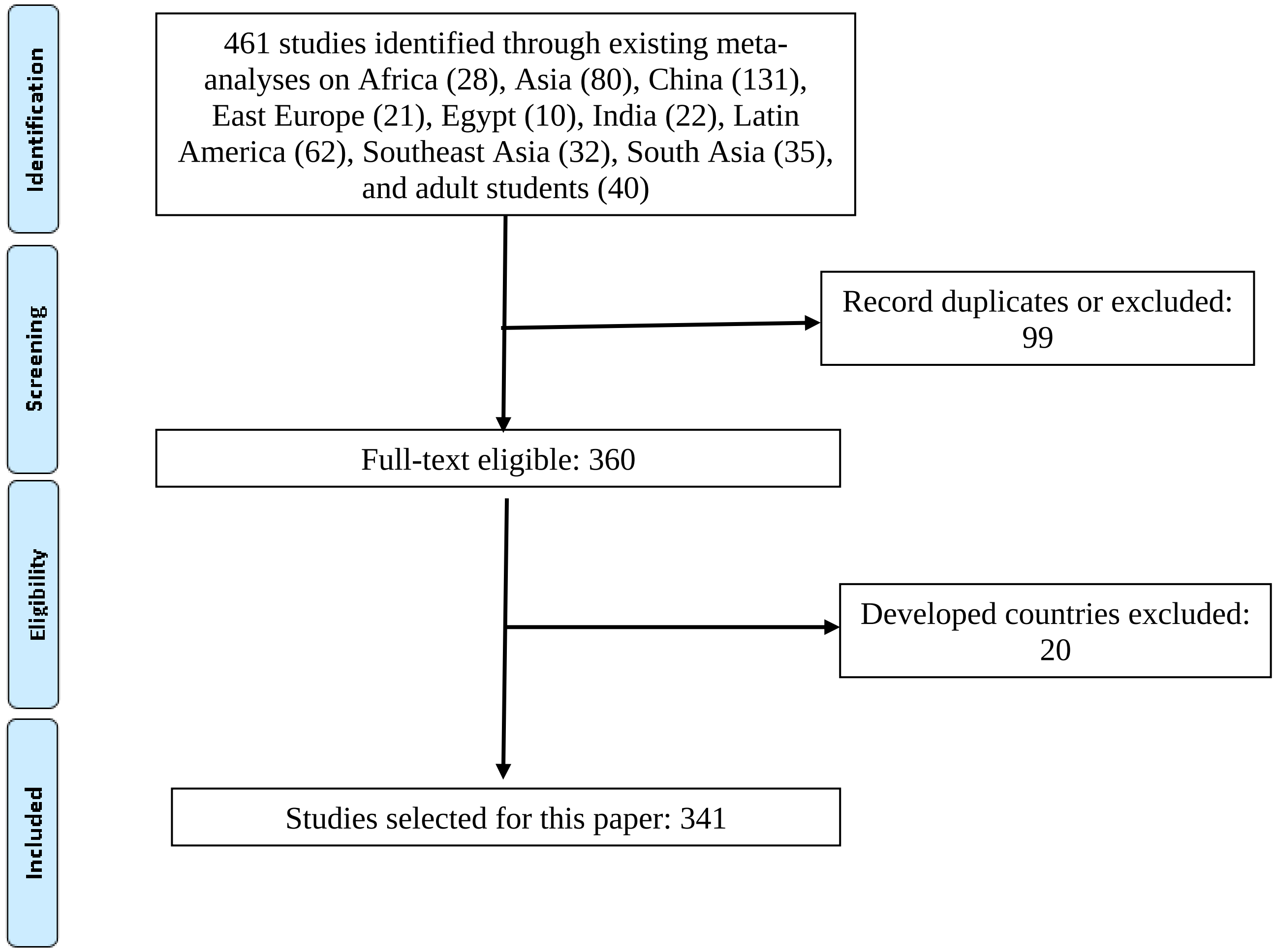

Figure S1. A PRISMA flow diagram

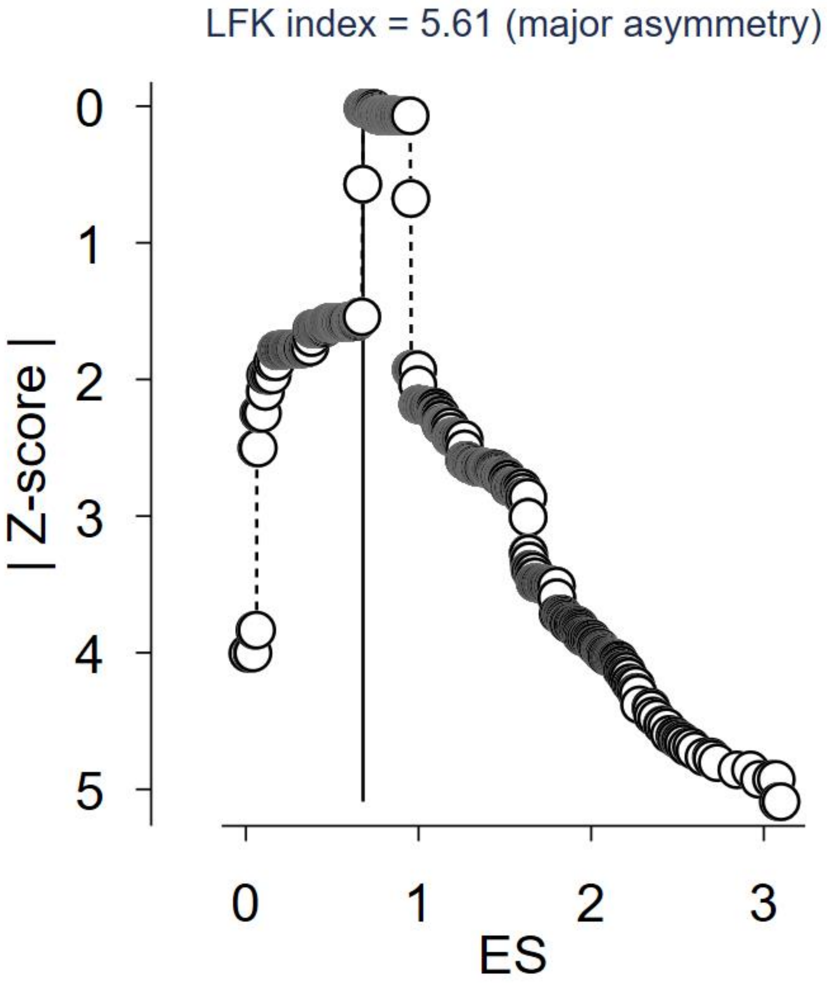

Figure S2. The DOI plot and the Luis Furuya–Kanamori (LFK) index

Table S1. The characteristics of the individual studies included the meta-analysis

Author_year	Country	Sample size	Population	Outcome	Instrument
		382	GHCW	ANX	GAD
Agberotimi et al. 2020 [1]	Nigeria	502	GP	ANX DEP INS	GAD, PHQ, ISI
Ahmed et al. 2020 [2]	Bangladesh	500	GP	ANX DEP	DASS
AlAteeq et al.2020 [3]	Saudi Arabia	502	GHCW	ANX DEP	GAD PHQ
Al-Dwaikat et al. 2020 [4]	Jordan	456	GS	ANX DEP	DASS
Ali et al. 2020 [5]	Pakistan	95	MS	ANX DEP	DASS
Almater et al. 2020 [6]	Saudi Arabia	107	GHCW	ANX DEP INS	GAD PHQ ISI
Alsairafi et al. 2021 [7]	Kuwait	559	GHCW	ANX DEP	GAD PHQ
		298	MS	ANX DEP	GAD PHQ
Alshekaili et al. 2020 [8]	Oman	574	FHCW	ANX DEP INS	DASS ISI
		565	GHCW	ANX DEP INS	DASS ISI
An et al. 2020 [9]	China	1103	GHCW	DEP	PHQ
Andrade et al. 2021 [10]	Brazil	380	GP	ANX DEP	GAI
Andrade et al. 2020 [11]	Egypt	320	FHCW	ANX, DEP	HADS
Antiporta et al. 2021 [12]	Peru	57446	GP	DEP	PHQ GAI GDS
		177	FHCW	ANX DEP	GAD
Antonijevic et al. 2020 [13]	Serbia	507	GHCW	ANX DEP	GAD BDI
Apisarnthanarak et al. 2020 [14]	Thailand	160	GHCW	ANX	GAD
Arafa et al. 2021a [15]	Egypt	1629	GP	ANX DEP	DASS
Arafa et al. 2021b [16]	Egypt & SaudiArabia	426	GHCW	ANX DEP	DASS
Aslan et al. 2020 [17]	Turkey	358	GS	ANX DEP	GAD PHQ
Aylie et al. 2020 [18]	Ethiopia	322	GP	ANX DEP	DASS
Bachilo et al. 2020 [19]	Russia	812	GHCW	ANX DEP	GAD PHQ
Badellino et al. 2021a [20]	Argentina	1985	GP	ANX DEP	GAD PHQ
Badellino et al. 2021b [21]	Argentina	2839	GP	DEP	PHQ
Balay-odao et al. 2021 [22]	Saudi Arabia	281	GHCW	ANX DEP	DASS
Barua et al. 2021 [23]	Bangladesh	370	FHCW	ANX DEP	GAD PHQ
Ben-Ezra et al. 2020 [24]	China	1134	GP	DIS	K6
Biswas & Biswas 2021 [25]	India	209	GS	ANX	GAD
Boluarte-Carbajal et al. 2021 [26]	Peru	204	GP	ANX DEP DIS	GAD PHQ HAMA
Brito-Marques et al. 2021 [27]	Brazil	332	GHCW	ANX DEP INS	GAD PHQ ISI PSS
Cai et al. 2020 [28]	China	348	FHCW	ANX DEP INS PTSD	GAD PHQ ISI IES
		361	GHCW	ANX DEP INS PTSD	GAD PHQ ISI IES
Campos et al. 2020 [29]	Brazil	12196	GP	ANX DEP	DASS
Campos et al. 2021a [30]	Brazil	66	GS	ANX DEP	DASS
Campos et al. 2021b [31]	Brazil	1609	GHCW	ANX DEP	DASS
Cao et al. 2020 [32]	China	37	FHCW	DEP	PHQ
Caycho-Rodriguez et al. 2021 [33]	Peru	274	GP	ANX DEP	GAD PHQ
Cayo-Rojas et al. 2021 [34]	Peru	403	GS	ANX	Zung_SRA
	Togo	242	GP	ANX	HSCL
	Haiti	225	GP	ANX	HSCL
	DRC	626	GP	ANX	HSCL
Cenat et al. 2020 [35]	Rwanda	174	GP	ANX	HSCL

Chakraborty et al. 2020[36]	India	507	GP	DEP	NA
Chatterjee et al. 2020[37]	India	152	GP	ANX DEP	DASS
Chen et al. 2020[38]	Ecuador	252	GHCW	ANX DIS	GAD
Chen et al. 2020a[39]	China	105	GHCW	ANX DEP	SAS K6
Chen et al. 2020b[40]	China	1921	GP	ANX	SAS
Chen et al. 2020c[41]	China	422	GHCW	DEP PTSD	SDS IES
		1071	GP	DEP PTSD	SDS IES
Chen et al. 2020d[42]	China	95	FHCW	ANX DEP INS PTSD	GAD PHQ ISI PCL
		77	GHCW	ANX DEP INS PTSD	GAD PHQ ISI PCL
Chen et al. 2020e[43]	China	902	GHCW	ANX DEP	GAD PHQ
Cheng et al. 2020[44]	China	534	GHCW	ANX INS	SAS
	India	384	GHCW	ANX DEP	DASS PSQI
	Indonesia	250	GHCW	ANX	DASS
	Malaysia	175	GHCW	ANX	DASS
Chew et al. 2020a[45]	Vietnam	60	GHCW	ANX	DASS
Chew et al. 2020b[46]	India	426	FHCW	ANX DEP	DASS
Choi et al. 2020[47]	China	500	GP	ANX DEP	GAD PHQ
Chow et al. 2021[48]	Malaysia	200	FHCW	ANX DEP	HADS
Civantos et al. 2020[49]	Brazil	163	GHCW	ANX DEP	GAD PHQ
Cortés-Álvarez et al. 2020[50]	Mexico	1105	GP	ANX DEP	DASS
Cypryańska & Nezlek 2020[51]	Poland	1028	GP	ANX	NA
da Silva et al. 2021[52]	Brazil	5879	GS	ANX	GAD
Dai et al. 2020[53]	China	4357	GHCW	DIS	GHQ
Dal'Bosco et al. 2020[54]	Brazil	88	FHCW	ANX DEP	HADS
Dangal & Bajracharya 2020[55]	Nepal	76	GS	ANX	GAD
Dantas et al. 2021[56]	Brazil	67	GHCW	ANX	BAI
Das et al. 2020[57]	India	422	FHCW	DEP	PHQ
De Boni et al. 2020[58]	Brazil	2842	GP	ANX DEP	GAD PHQ
Deng et al. 2020[59]	China	1607	GS	ANX DEP	DASS
Dong et al. 2020[60]	China	4618	FHCW	DIS	HEI
Du et al. 2020[61]	China	134	FHCW	ANX DEP	BAI
Duy et al. 2020[62]	Vietnam	61	GHCW	ANX DEP	DASS BDI
Dzhambov et al. 2020[63]	Bulgaria	323	GS	ANX DEP	GAD PHQ
Elezi et al. 2021[64]	Albania	1678	GP	ANX DEP	GAD PHQ
Elhadi et al. 2020a[65]	Libya	745	GHCW	ANX DEP	HADS
Elhadi et al. 2020b[66]	Libya	2430	MS	ANX DEP	GAD PHQ
Elhadi et al. 2020c[67]	Libya	154	FHCW	ANX DEP	HADS
Elhai et al. 2020[68]	China	908	GP	ANX DEP	GAD
Elkholy et al. 2020[69]	Egypt	457	FHCW	ANX DEP INS	GAD PHQ ISI
Espinosa-Guerra et al. 2021 [70]	Panama	512	GP	ANX DEP DIS	GAD PHQ
Essangri et al. 2021 [71]	Morocco	549	MS	ANX DEP INS	GAD PHQ ISI IES
Esteves et al. 2021 [72]	Brazil	208	GS	ANX DEP	DASS
Faize & Husain 2021 [73]	Pakistan	342	GS	ANX	GAD
Fang 2020 [74]	China	191	GP	DEP	PHQ
Fauzi et al. 2020 [75]	Malaysia	1050	GHCW	ANX DEP	DASS
Feng 2020 [76]	China	671	GP	ANX DEP PTSD	GAD PHQ PC

Fernández et al. 2020 [77]	Argentina	4408	GP	ANX DEP DIS	BSI
Fernandez et al. 2021 [78]	Brazil	1050	GS	ANX	GAD GSI
Ferreira et al. 2021 [79]	Brazil	1130	GP	ANX DEP	DASS
Feter et al. 2021 [80]	Brazil	1767	GP	ANX DEP	HADS
Flores-Torres et al. 2021 [81]	Mexico	2016	GP	ANX DEP	GAD CES
Fong et al. 2020 [82]	China	590	GP	DEP	PHQ
Fu et al. 2020 [83]	China	1242	GP	ANX DEP INS	GAD PHQ
Gallopeni et al. 2000 [84]	Kosovo	592	GHCW	ANX DEP	HADS AIS
Gao et al. 2020 [85]	China	4872	GP	ANX DEP	GAD WHO
García-Espinosa et al. 2021 [86]	Mexico	1149	GS	ANX DEP	GAD PHQ
Gaş et al. 2021 [87]	Turkey	699	MS	ANX DEP	DASS
Ge et al. 2020 [88]	China	2009	GS	ANX INS	GAD
Giardino et al. 2020 [89]	Argentina	1059	GHCW	ANX DEP INS	GADS ISI
Goularte et al. 2020 [90]	Brazil	1996	GP	ANX DEP INS	DSM ISI
Guiroy et al. 2020 [91]	NA	204	GHCW	DEP	PHQ PROMIS
Guo et al. 2020a [92]	China	3351	FHCW	ANX DEP	SAS SDS
	China	7767	GHCW	ANX DEP	SAS SDS
Guo et al. 2020b [93]	China	2441	GP	DEP INS	CES SDS PSQI
Gupta et al. 2020a [94]	India	368	GHCW	ANX	GAD
Gupta et al. 2020b [95]	Nepal	150	GHCW	ANX DEP	GAD PHQ
Gupta et al. 2020c [96]	India	749	GHCW	ANX DEP	HADS
Gupta et al. 2021d [97]	India	1124	GHCW	ANX	HADS
Han et al. 2020 [98]	China	21199	FHCW	ANX DEP	SAS
Hasan et al. 2020 [99]	Pakistan	151	GHCW	ANX	GAD
Hikmah et al. 2020 [100]	Indonesia	267	GP	ANX	HAMA
Hong et al. 2020 [101]	China	4692	FHCW	ANX DEP	GAD PHQ
Hossain et al.2020 [102]	Bangladesh	880	GP	ANX	GAD
Hou et al. 2020 [103]	China	3063	GP	ANX DEP	GAD PHQ
Hu et al. 2020a [104]	China	645	FHCW	ANX DEP	GAD PHQ
Hu et al. 2020b [105]	China	2014	FHCW	ANX DEP	SAS
Huang et al. 2020a [106]	China	6261	GP	ANX DEP	SAS SDS
Huang et al. 2020b [107]	China	364	FHCW	ANX	SAS PHQ
Huang et al. 2020c [108]	China	1172	GP	ANX INS	GAD
Idrissi et al. 2020 [109]	Morocco	846	GP	ANX DEP INS	HARS ISI
Imran et al. 2020a [110]	Pakistan	10178	MS	ANX DEP	GAD PHQ BDI
Imran et al. 2020b [111]	Pakistan	337	GHCW	ANX DEP INS	GAD PHQ ISI
Islam et al. 2020 [112]	Bangladesh	476	GS	ANX DEP	GAD PHQ
Jain et al. 2020 [113]	India	512	GHCW	ANX INS	GAD
Jia et al. 2021 [114]	China	740	GS	ANX	SAS
Jin et al. 2020 [115]	China	175	GP	ANX	SAS
Jindal et al. 2020 [116]	India	956	MS	ANX	GAD
Juan et al 2020 [117]	China	416	FHCW	ANX DEP	GAD PHQ
Kafle et al. 2021 [118]	Nepal	254	GHCW	DIS	CPDI
Kalok et al. 2020 [119]	Malaysia	772	GS	ANX DEP	DASS
Kamaludin et al. 2020 [120]	Malaysia	983	GS	ANX	SAS
Karpenko et al. 2020 [121]	Russia	352	GP	ANX DEP	HADS
Kassaw 2020 [122]	Ethiopia	382	GP	ANX DEP	DASS
Keubo et al. 2020 [123]	Cameroon	292	FHCW	ANX DEP	HAD

Khan et al. 2020 [124]	Bangladesh	505	GS	ANX DEP	DASS
Khanal et al. 2020a [125]	Nepal	475	GHCW	ANX DEP INS	HADS
Khanna et al. 2020b [126]	India	2355	GHCW	DEP INS	PHQ ISI
Khatun et al. 2021 [127]	Bangladesh	114	GHCW	ANX DEP	GAD PHQ
Khoshaim et al. 2020 [128]	Saudi Arabia	400	GS	ANX	Zung_SRA
Kiat et al. 2021 [129]	Malaysia	105	FHCW	ANX DEP	DASS
Kibret et al. 2020 [130]	Ethiopia	305	GHCW	ANX	GAD
Kim et al. 2020 [131]	South Africa	211	GP	DEP	CES
Koksal et al. 2020 [132]	Turkey	702	GHCW	ANX DEP	HADS
Kounou et al. 2020 [133]	Togo	62	GHCW	ANX DEP	GAD PHQ
Kumar et al. 2021 [134]	Pakistan	224	FHCW	ANX DEP	DASS
Labrague & Santos 2020 [135]	Philippines	325	FHCW	ANX	Anxiety Scale
Lai et al. 2020b [136]	China	1257	GHCW	ANX DEP INS DIS	GAD PHQ ISI IES
Lam et al. 2020 [137]	China	510	FHCW	DEP	PHQ
Landaeta-DÃaz et al. 2021 [138]	Chile	1725	GP	ANX	BAI
Le et al. 2020 [139]	Vietnam	742	GP	ANX DEP	DASS
Lei et al. 2020 [140]	China	1593	GP	ANX DEP	SAS SDS
Leng et al.2020 [141]	China	90	FHCW	PTSD	PCL
Li et al. 2020 [142]	China	1109	GP	PTSD	IES
Li et al. 2020a[143]	China	219	FHCW	INS	AIS
	China	729	GHCW	INS	AIS
Li et al. 2020b[144]	China	4396	GHCW	ANX DEP	GAD PHQ
Li et al. 2020c [145]	China	3637	GP	ANX DEP INS	GAD PHQ ISI
Li et al. 2020d [146]	China	1676	GS	ANX	HAI
Li et al. 2020e [147]	China	908	GHCW	ANX DEP	SAS SDS
Li et al. 2020f [148]	China	88611	GP	ANX	GAD
Li et al. 2020g [149]	China	176	FHCW	ANX	HAMA
Li et al. 2020h [150]	China	225	FHCW	ANX DEP PTSD	DASS IES
Li et al. 2020i [151]	China	356	FHCW	PTSD	PCL
Li et al. 2020j [152]	China	150	FHCW	ANX DEP	HAMA
Liang et al. 2020 [153]	China	138	FHCW	ANX DEP INS	GAD PHQ ISI
		1074	GP	ANX DEP INS	GAD PHQ ISI
Liang et al. 2020b [154]	China	4164	GS	DEP	PHQ
Lin et al. 2020a [155]	China	2316	GHCW	ANX DEP INS	GAD PHQ ISI
Lin et al. 2020b [156]	China	5461	GP	ANX DEP INS	GAD PHQ ISI
Lin et al. 2020c [157]	China	628	GS	DEP	CES
Lingawi et al. 2020 [158]	Saudi Arabia	258	GS	ANX	GAD
Liu et al. 2020a [159]	China	512	GHCW	ANX	SAS
Liu et al. 2020b [160]	China	14592	GP	ANX DEP	GAD PHQ
Liu et al. 2020c [161]	China	285	GP	PTSD	PCL
Liu et al. 2020d [162]	China	4976	GHCW	ANX DEP DIS	SAS SDS SRQ
Liu et al. 2020e [163]	China	4991	GP	ANX	SAS
Liu et al. 2020f [164]	China	574	GP	DEP	SDS
Liu et al. 2020g [165]	China	317	FHCW	ANX DEP	GAD PHQ
		719	GHCW	ANX DEP	GAD PHQ
Liu et al. 2020h [166]	China	742	FHCW	ANX DEP	DASS
		1289	GHCW	ANX DEP	DASS

Lu et al. 2020a [167]	China	2042	GHCW	ANX DEP	HAMA
		257	GP	ANX DEP	HAMA
Lu et al. 2020b [168]	China	1848	GHCW	DEP	CES
Lu et al. 2020c [169]	China	382	FHCW	ANX DEP PTSD	GAD PHQ PCL
		1035	GP	ANX DEP PTSD	GAD PHQ PCL
Ma et al. 2020 [170]	China	746217	GS	ANX DEP	GAD PHQ
		1216	GHCW	DEP	GHQ
Maciaszek et al. 2020 [171]	Poland	823	GP	DEP	GHQ
Malgor et al. 2020 [172]	Brazil	405	GHCW	ANX	GAD
Mamun et al. 2021 [173]	Bangladesh	10067	GP	DEP	PHQ
Manjareeka & Pathak 2021 [174]	India	101	MS	ANX	STAI
Markovic et al. 2020 [175]	Serbia	110	GP	ANX DEP	BAI SDS
Martinez et al. 2020 [176]	Brazil	1613	GP	ANX DEP	HADS
Mautong et al. 2021 [177]	Ecuador	626	GP	ANX DEP	DASS
Mboua et al. 2020 [178]	Cameroon	332	GHCW	ANX DEP	HADS
Mechili et al. 2020 [179]	Albania	863	GS	DEP	PHQ
Medeiros et al. 2020 [180]	Brazil	113	GS	ANX DEP	HADS
Mekonen et al. 2020 [181]	Ethiopia	302	GHCW	ANX DEP	DASS
Mendonca et al. 2021 [182]	Brazil	3071	GS	ANX DEP	GAD PHQ
Mi et al. 2020 [183]	China	1029	GHCW	ANX DEP	GAD PHQ
Mier-Bolio et al. 2020 [184]	Mexico	31	GHCW	ANX	GAD
Mishra et al. 2020 [185]	India	716	GHCW	DIS	SRQ
Mongkhon et al. 2020 [186]	Thailand	4004	GP	ANX DEP INS	GAD PHQ ISI
Monterrosa-Castro et al. 2020 [187]	Colombia	531	GHCW	ANX	GAD
Mora-Magana et al. 2020 [188]	Mexico	231	GHCW	ANX DEP	GAD PHQ
Mosolova et al. 2021 [189]	Russia	1090	FHCW	ANX	GAD
Mota et al. 2021 [190]	Brazil	710	GHCW	INS	ISI
Mulatu et al. 2020 [191]	Ethiopia	420	GHCW	ANX DEP INS	GAD PHQ ISI
Naidoo et al. 2020 [192]	South Africa	150	GHCW	ANX DEP	GAD PHQ
Nam et al. 2021 [193]	Vietnam	1249	GP	ANX	GAD
		1163	GHCW	ANX DEP	GAD PHQ
Naser et al. 2020 [194]	Jordan	1798	GP	ANX DEP	GAD PHQ
		1165	GS	ANX DEP	GAD PHQ
Nayak et al. 2021 [195]	Trinidad and Tobago	395	GHCW	ANX DEP	DASS
Ng et al. 2020a [196]	Malaysia	22	GHCW	ANX DEP	DASS
Ni et al. 2020a [197]	China	214	GHCW	ANX DEP	GAD PHQ
		1577	GP	ANX DEP	GAD PHQ
Ni et al. 2020b [198]	China	2551	GP	ANX DEP	GAD PHQ
Ning et al. 2020 [199]	China	612	GHCW	ANX DEP	SAS SDS
Nihmath et al. 2020 [200]	India	359	MS	ANX DEP	GAD CES
Noor et al. 2021 [201]	Malaysia	160	FHCW	ANX	HADS
Omar et al. 2021 [202]	Egypt	217	GP	ANX DEP	GAD PHQ
Pan et al. 2020a [203]	China	194	GHCW	ANX DEP	GAD PHQ
Pan et al. 2020b [204]	China	3035	GP	DEP	PHQ
Pan et al. 2020c [205]	China	423	GHCW	ANX	SAS
Pandey et al. 2020a [206]	Nepal	404	FHCW	ANX DEP	DASS

Pandey et al. 2020b [207]	India	82	MS	ANX DEP	GAD PHQ
Passos et al. 2020 [208]	Brazil	289	GP	ANX DEP	GAD PHQ
Paz et al. 2020 [209]	Ecuador	453	GP	ANX DEP	GAD PHQ
Perera et al. 2021 [210]	Sri Lanka	512	GHCW	ANX DEP	GAD CES
Perveen et al. 2020 [211]	Malaysia	716	GHCW	ANX DEP	DASS
Puccinelli et al. 2021 [212]	Brazil	57	GP	ANX DEP	GAD PHQ
Qi et al. 2020 [213]	China	801	FHCW	INS	PSQI
		505	GHCW	INS	PSQI
Qian et al. 2020 [214]	China	510	GP	ANX	GAD
Qiu et al. 2020 [215]	China	52730	GP	DIS	CPDI
Que et al 2020 [216]	China	2285	GHCW	ANX DEP INS	GAD PHQ ISI
Rakhmanov et al. 2020 [217]	Nigeria	69	GP	ANX DEP	SRQ
Reddy et al. 2020 [218]	India	891	GP	ANX DEP	DASS
Ren et al. 2020 [219]	China	6130	GP	ANX DEP	GAD PHQ
Ribeiro et al. 2021 [220]	Brazil	494	GP	ANX DEP	GAD CES
		1389	FHCW	ANX DEP INS	PHQ DSM ICD
Robles et al. 2020 [221]	Mexico	4420	GHCW	ANX DEP INS	PHQ DSM ICD
Rogowska et al. 2020a [222]	Poland	914	GS	ANX	GAD
Rogowska et al. 2020b [223]	Ukraine	1512	GS	ANX DEP	GAD PHQ
Rashad et al. 2020 [224]	Pakistan	431	GHCW	ANX	GAD
Saeed et al. 2021 [225]	Pakistan	278	GHCW	DEP	SRQ
Safa et al. 2021 [226]	Bangladesh	425	MS	ANX DEP	HADS
Sagaon-Teyssier et al. 2020 [227]	Mali	135	GHCW	ANX DEP INS	GAD PHQ ISI
Șahin et al. 2020 [228]	Turkey	939	GHCW	ANX DEP INS	GAD PHQ ISI
Samaniego et al. 2020 [229]	Paraguay	126	GHCW	ANX DEP DIS INS	GAD PHQ ISI
Sandesh et al. 2020 [230]	Pakistan	112	FHCW	ANX DEP	DASS
Schmitt et al. 2021 [231]	Brazil	3274	GP	DEP	PHQ
Schuch et al. 2020 [232]	Brazil	937	GP	DEP	BDI
Scotta & Miranda 2020 [233]	Argentina	584	GS	INS	ISI
Secosan et al. 020 [234]	Romania	126	FHCW	INS	ISI
Sediri et al. 2020 [235]	Tunisia	751	GP	ANX DEP	DASS
Serafim et al. 2020 [236]	Brazil	3000	GP	ANX DEP	DASS
Shi et al. 2020 [237]	China	9725	FHCW	ANX DEP INS	GAD PHQ ISI
		46954	GP	ANX DEP INS	GAD PHQ ISI
Shrestha et al. 2020[238]	Nepal	101	GHCW	ANX	GAD
Shuwiekh et al. 2020[239]	Egypt	255	GP	ANX DEP	GAD PHQ
Si et al. 2020[240]	China	863	GHCW	ANX DEP PTSD	DASS IES
Sil et al. 2020 [241]	India	41	FHCW	DEP	PHQ
Šljivo et al. 2020 [242]	Bosnia and Herzegovina	1201	GP	DEP	PHQ
Sögüt & Cangöl 2021 [243]	Turkey	972	MS	ANX	BAI
Song et al. 2020a [244]	China	14825	GHCW	DEP PTSD	CES PCL
Song et al. 2020b [245]	China	709	GP	ANX DEP INS	GAD CES ISI
Souilhi et al. 2020 [246]	Tunisia	48	GHCW	ANX DEP INS	GAD PHQ ISI
Souza et al. 2021 [247]	Brazil	3200	GP	ANX DEP	DASS
		118	FHCW	ANX DEP	GAD SDS
Stojanov et al. 2020 [248]	Serbia	83	GHCW	ANX DEP	GAD SDS

Su et al. 2020 [249]	China	403	GP	ANX	GAD
Sun et al. 2020a [250]	China	2091	GP	PTSD	PCL
Sun et al. 2020b [251]	China	170	FHCW	ANX DEP	DASS
Sun et al. 2020c [252]	China	472	GP	DEP	CES
Sun et al. 2020d [253]	China	536	GHCW	ANX DEP	GAD PHQ
Sun et al. 2020e [254]	China	474	MS	ANX	SAS
Sundarasen et al. 2020 [255]	Malaysia	983	GS	ANX	SAS
		291	FHCW	ANX DEP	ZAS CESDR
Sunjaya et al. 2021 [256]	Indonesia	253	GHCW	ANX DEP	ZAS CESDR
Suryavanshi et al. 2020 [257]	India	197	GHCW	ANX DEP	GAD PHQ
Tan et al. 2020 [258]	China	673	GP	ANX DEP PTSD INS	DASS IES ISI
Tasnim et al. 2020 [259]	Bangladesh	3331	GS	ANX DEP	DASS
Temsah et al. 2020 [260]	Saudi Arabia	582	GHCW	ANX	GAD
Teng et al. 2020 [261]	China	398	GHCW	ANX DEP	SAS PHQ
Teshome et al. 2020 [262]	Ethiopia	798	GHCW	ANX	GAD
Torrente et al. 2020 [263]	Argentina	10053	GP	ANX DEP	GAD PHQ
Torrente et al. 2021 [264]	Argentina	3617	GP	ANX DEP	GAD PHQ
Tran et al. 2020 [265]	Vietnam	7124	GP	ANX DEP	GAD PHQ
Tu et al. 2020 [266]	China	100	GHCW	ANX DEP INS	GAD PHQ ISI
Vala et al. 2020 [267]	India	250	MS	ANX DEP	DASS
Venugopal et al. 2020 [268]	India	453	GP	DIS	GHQ
Verma et al .2020 [269]	India	354	GP	ANX DEP	DASS
Villela et al. 2021 [270]	Brazil	295	GHCW	ANX DEP	HADS
Vitorino et al. 2021 [271]	Brazil	1156	GP	ANX DEP	GAD PHQ
Vujcic et al. 2020 [272]	Serbia	1057	GP	ANX DEP	DASS
Wang & Zhang 2020 [273]	China	1172	GS	ANX	SAS
Wang & Zhao 2020 [274]	China	3611	GS	ANX	SAS
Wang et al. 2020a [275]	China	600	GP	ANX DEP	SAS SDS
Wang et al. 2020b [276]	China	123	GHCW	ANX DEP INS	SAS SDS PSQI
Wang et al. 2020c [277]	China	1045	GHCW	ANX DEP INS	HADS ISI
Wang et al. 2020d [278]	China	6437	GP	INS	PSQI
Wang et al. 2020e [279]	China	274	FHCW	INS	PSQI
Wang et al. 2020f [280]	China	179	FHCW	ANX DEP INS	GAD PHQ ISI
		16066	GP	ANX DEP INS	GAD PHQ ISI
Wang et al. 2020g [281]	China	661	FHCW	ANX DEP INS	HADS PSQI
		853	GHCW	ANX DEP INS	HADS PSQI
		487	GP	ANX DEP INS	HADS PSQI
Wang et al. 2020h [282]	China	202	FHCW	PTSD	PCL
Wang et al. 2020i [283]	China	2540	GP	ANX DEP	GAD PHQ
Wang et al. 2020j [284]	China	742	FHCW	ANX DEP PTSD	GAD PHQ IES
		1155	GHCW	ANX DEP PTSD	GAD PHQ IES
Wang et al. 2020k [285]	China	1599	GP	DIS	K6
Wang et al. 2020l [286]	China	3092	GS	ANX INS	GAD SRSS
		206	FHCW	ANX DEP INS	GAD PHQ ISI
Wankowicz et al. 2020 [287]	Poland	235	GHCW	ANX DEP INS	GAD PHQ ISI
Werneck et al. 2020 [288]	Brazil	43995	GP	ANX	NA

Wilson et al. 2020 [289]	India	350	FHCW	ANX DEP	GAD PHQ
Wong & Alias 2020 [290]	Malaysia	581	GP	ANX	STAI
Wu et al. 2020a [291]	China	24789	GP	ANX DEP	HADS
Wu et al. 2020b [292]	China	304	GHCW	DIS	K6
Wu et al. 2020c [293]	China	330	GP	PTSD	PCL
Xiang et al. 2020 [294]	China	1421	GS	ANX	SAS
Xiao et al. 2020a [295]	China	958	GHCW	ANX DEP	HADS
Xiao et al. 2020b [296]	China	933	MS	ANX DEP	GAD PHQ
Xing et al. 2020 [297]	China	309	FHCW	ANX DEP	SAS SDS
Xiong et al. 2020 [298]	China	231	GHCW	ANX DEP	GAD PHQ
Xu et al. 2020 [299]	China	8817	GHCW	ANX DEP	GAD PHQ
Yanez et al. 2020 [300]	Peru	303	GHCW	ANX DIS	GAD K6
Yang et al. 2020a [301]	China	449	GHCW	ANX	SAS
Yang et al. 2020b [302]	China	2410	GP	INS	PSQI
Yıldırım et al. 2021 [303]	Turkey	245	FHCW	ANX DEP	DASS
Yilmaz et al. 2020 [304]	Turkey	240	GHCW	ANX	BAI
Yin et al. 2020a [305]	China	8151	GP	ANX DEP	GAD PHQ
Yin et al. 2020b [306]	China	371	GHCW	PTSD	PCL
Ying et al. 2020 [307]	China	845	GP	ANX DEP	GAD PHQ
Yitayih et al. 2020 [308]	Ethiopia	249	GHCW	INS	ISI
Youssef et al. 2020 [309]	Egypt	540	GP	ANX DEP INS	DASS ISI
Yu et al. 2020a [310]	China	1588	GP	DIS	K6
Yu et al. 2020b [311]	China	1138	GP	INS	ISI
Yu et al. 2020c [312]	China	290	FHCW	ANX	GAD
Yu et al. 2021d [313]	China	1681	GS	DEP	CES
Zhan et al. 2020a [314]	China	2667	FHCW	ANX DEP	GAD PHQ
Zhan et al. 2020b [315]	China	1794	FHCW	INS	AIS
Zhang et al. 2020a [316]	China	1563	GHCW	ANX DEP INS PTSD	GAD PHQ ISI IES
Zhang et al. 2020b [317]	China	98	GP	ANX DEP	GAD PHQ
Zhang et al. 2020c [318]	China	369	GP	DIS	K6
Zhang et al. 2020d [319]	China	927	GHCW	ANX DEP INS	GAD PHQ ISI
		1255	GP	ANX DEP INS	GAD PHQ ISI
Zhang and Ma 2020 [320]	China	263	GP	PTSD	CIES
Zhang et al. 2020f [321]	China	966	FHCW	ANX DEP	GAD PHQ
Zhang et al. 2020g [322]	China	421	FHCW	PTSD	PCL
		642	GHCW	ANX DEP INS PTSD	HADS ISI
Zhang et al. 2020h [323]	China	1342	GP	DEP	PHQ
Zhang et al. 2020i [324]	China	123768	GP	ANX DEP	SAS SDS
Zhang et al. 2020j [325]	China	2640	GP	ANX DEP	SAS SDS
Zhang et al. 2020k [326]	China	66	GS	ANX DEP INS	DASS PSQI
Zhang et al. 2020l [327]	Peru	671	GP	DIS	CPDI
Zhang et al. 2020m [328]	Brazil	482	GP	ANX DEP	GAD PHQ
Zhang et al. 2021o [329]	Brazil	638	GP	DIS	CPDI
Zhang et al. 2021p [330]	Bolivia	240	GHCW	ANX DIS	GAD K6
Zhao et al. 2020a [331]	China	972	FHCW	ANX DEP INS	GAD PHQ ISI
Zhao et al. 2020b [332]	China	1501	GP	ANX DEP	GAD PHQ
Zhao et al. 2020c [333]	China	1630	GP	ANX INS	SAS PSQI

Zhao et al. 2020d [334]	China	515	GS	ANX DEP	SAS SDS
Zhou et al. 2020a [335]	China	1931	FHCW	INS	PSQI
Zhou et al. 2020b [336]	China	606	FHCW	ANX DEP INS	GAD PHQ ISI
		1099	GP	ANX DEP INS	GAD PHQ ISI
Zhu et al. 2020a [337]	China	5281	GP	ANX DEP	GAD PHQ
Zhu et al. 2020b [338]	China	453	GHCW	ANX	SAS
Zhu et al. 2020c [339]	China	165	FHCW	ANX DEP	SAS SDS
Zhu et al. 2020d [340]	China	320	FHCW	ANX DEP	GAD PHQ
		538	GHCW	ANX DEP	GAD PHQ
Zubayer et al. 2020 [341]	Bangladesh	1146	GP	ANX DEP	DASS

Note: ANX= Anxiety, DEP= DEP, INS= INS, DIS=Distress, PTSD= FHCW=Frontline Healthcare Worker, GHCW = General Healthcare Worker, GP = GP

Table S2 Instrument of mental health symptoms used in the individual studies in the meta-analysis

**Outcome**	**Instrument used**	**Full name of instrument**
**Anxiety**	Anxiety Scale	The covid-19 Anxiety Scale
	BAI	Beck Anxiety Inventory
	BSI-18/53	Brief Symptom Inventory – 18/53 items
	DASS-21	DEP Anxiety Stress Scale -21 items
	DSM-5	The Diagnostic and Statistical Manual of Mental Disorders, Fifth Edition
	GAD-2/7	Generalized Anxiety Disorder- 2/7item
	GADS	Goldberg DEP and anxiety scale
	GAI	The Geriatric Anxiety Inventory
	HADS	Hospital Anxiety and DEP Scale
	HADS-A	Hospital Anxiety and DEP Scale - Anxiety
	HAI	Health Anxiety Inventory
	HAMA	Hamilton Anxiety Scale
	HARS	The Hamilton Anxiety Rating Scale
	HSCL	The Hopkins Symptom Checklist
	ICD-11 PHC	International Classification of Diseases - 11 Primary Health Care
	MINI	The Mini-International Neuropsychiatric Interview
	SAS (Zung's SRA/ZAS)	Zung's self-rating anxiety scale
	SATI	State - trait anxiety inventory
	SATI-6	State - trait anxiety inventory – 6 items
	SRQ-20	Self-Reporting Questionnaire – 20 items
**DEP**	BDI	Beck DEP Index
	BDI-II	Beck DEP Inventory—II
	BSI-18	Brief Symptom Inventory – 18 items
	BSI-53	Brief Symptom Inventory – 53 items
	CES-D	The Center for Epidemiological Studies-DEP
	CES-D-9/10/20	The Center for Epidemiological Studies-DEP – 9 items/10 items/20 items
	CESDR-10	The Center for Epidemiologic Studies Short DEP Scale – 10 items
	CES-R-10	The Center for Epidemiologic Studies DEP Scale Revised – 10 items
	DASS-21	DEP Anxiety Stress Scale – 21 items
	DSM-5	The Diagnostic and Statistical Manual of Mental Disorders, Fifth Edition
	GADS	Goldberg DEP and anxiety scale
	GDS	Geriatric DEP Scale
	GHQ-28	The General Health Questionnaire – 28 *items*

	HADS	Hospital Anxiety and DEP Scale
	HADS-D	Hospital Anxiety and DEP Scale - DEP
	HAMD	The Hamilton Rating Scale for ***DEP***
	MINI	The Mini-International Neuropsychiatric Interview
	NSS	The Nursing Stress Scale
	PHQ-2/8/9	Patient Health Questionnaire – 2/8/9items
	PROMIS DEP	Patient-Reported Outcomes Measurement Information System - DEP
	SDS	Self-rating DEP scale
	SRQ-20	Self-Reporting Questionnaire – 20 items
	SRQ-21	Self-Reporting Questionnaire – 21 items
	WHO-5	The World Health Organisation- Five Well-Being Index
**Distress**	CPDI	COVID-19 Peritraumatic Distress Index
	GHQ-12	General Health Questionnaire – 12 items
	GHQ-28	General Health Questionnaire – 28 items
	GSI	Overall Psychological Distress
	HEI	Huaxi emotional-distress index
	IES-R	IMPACT OF EVENTS SCALE-Revised
	K6	K-6 distress scale
	PSS-10	The Perceived Stress Scale – 10 items
	SRQ-20	Self-Reporting Questionnaire – 20 items
**INS**	AIS	Athens INS Scale
	DSM	Diagnostic and Statistical Manual of Mental Disorders
	DSM-5	Diagnostic and Statistical Manual of Mental Disorders, Fifth Edition
	GHQ-12	General Health Questionnaire – *12 items*
	ISI/ISI-7	The INS Severity Index / *-7items*
	PSQI	The Pittsburgh Sleep Quality Index
	SRSS	Self-rating sleeping scaling
**PTSD**	CIES	COVID Isolation Eating Scale
	IES-6	Impact of Event Scale – 6 items
	IES-R	Impact of Event Scale- Revised
	PCL-5	PTSD Checklist for DSM-5
	PCL-C	PTSD Checklist – Civilian Version
	PC-PTSD	Primary Care PTSD Screen for DSM-5

References

1. Agberotimi SF, Akinsola OS, Oguntayo R, et al. Interactions Between Socioeconomic Status and Mental Health Outcomes in the Nigerian Context Amid COVID-19 Pandemic: A Comparative Study. *Frontiers in Psychology* 2020 doi: https://doi.org/10.3389/fpsyg.2020.559819
2. Ahmed O, Ahmed MZ, Alim SMAHM, et al. COVID-19 outbreak in Bangladesh and associated psychological problems: An online survey. *Death Studies* 2020;43: 1 - 2. doi: 10.1080/07481187.2020.1818884
3. AlAteeg DA, Aljhani S, Althiyabi I, et al. Mental health among healthcare providers during coronavirus disease (COVID-19) outbreak in Saudi Arabia. *Journal of Infection and Public Health* 2020;13(10):1432 - 37. doi: 10.1016/j.jiph.2020.08.013
4. Al-Dwaikat TN, Aldalaykeh M, Taan Wa, et al. The relationship between social networking sites usage and psychological distress among undergraduate students during COVID-19 lockdown. *Heliyon* 2020;6(12) doi: 10.1016/j.heliyon.2020.e05695
5. Wong SYS, Zhang D, Sit RWS, et al. COVID-19 Online Teaching and its Impact on Psychological Health in Higher Education: A Cross Sectional Study on Medical Students of 1st 2nd and 3rd Year MBBS. *Journal of Research in Medical and Dental Science* 2020;70(700) doi: 10.3399/bjgp20X713021
6. Abouammoh M, Almater A, Tobaigy M, et al. Effect of 2019 Coronavirus Pandemic on Ophthalmologists Practicing in Saudi Arabia: A Psychological Health Assessment. *Middle East African Journal of Ophthalmology* 2020;27(2):79. doi: 10.4103/meajo.MEAJO_220_20
7. Alsairafi Z, Naser AY, Alsaleh FM, et al. Mental Health Status of Healthcare Professionals and Students of Health Sciences Faculties in Kuwait during the COVID-19 Pandemic. *International Journal of Environmental Research and Public Health* 2021;18(4):2203. doi: 10.3390/ijerph18042203
8. Alshekaili M, Hassan W, Said NA, et al. Factors associated with mental health outcomes across healthcare settings in Oman during COVID-19: frontline versus non-frontline healthcare workers. *BMJ Open* 2020;10(10) doi: 10.1136/bmjopen-2020-042030
9. An Y, Yang Y, Wang A, et al. Prevalence of depression and its impact on quality of life among frontline nurses in emergency departments during the COVID-19 outbreak. *Journal of Affective Disorders* 2020;276:312-15. doi: 10.1016/j.jad.2020.06.047 [published Online First: 2020/09/03]
10. de Oliveira Andrade N, Correia Silva Azambuja H, Carvalho Reis Martins T, et al. Factors associated with depressive and anxiety symptoms in older adults during the COVID-19 pandemic: a Brazilian study. *Aging & mental health* 2021;2021:1-8. doi: 10.1080/13607863.2021.1942431
11. Andrade ND, Azambuja HCS, Martins TCR, et al. Factors associated with depressive and anxiety symptoms in older adults during the COVID-19 pandemic: a Brazilian study. *Aging & Mental Health*:8. doi: 10.1080/13607863.2021.1942431
12. Antiporta DA, Cutipe YL, Mendoza M, et al. Depressive symptoms among Peruvian adult residents amidst a National Lockdown during the COVID-19 pandemic. *BMC Psychiatry* 2021;21(1):111-22. doi: 10.1186/s12888-021-03107-3

13. Antonijevic J, Binic I, Zikic O, et al. Mental health of medical personnel during the COVID-19 pandemic. *Brain and Behavior* 2020;10(12) doi: 10.1002/brb3.1881
14. Apisarnthanarak A, Apisarnthanarak P, Siripraparat C, et al. Impact of anxiety and fear for COVID-19 toward infection control practices among Thai healthcare workers. *Infection Control & Hospital Epidemiology* 2020;41(9):1093-94.
15. Ahmed Arafa AM, Lamiaa Saleh & Shaimaa Senosy Psychological Impacts of the COVID-19 Pandemic on the Public in Egypt. *Health Promotion International* 2021;36(4):1116-25. doi: https://doi.org/10.1093/heapro/daaa147
16. Arafa A, Mohammed Z, Mahmoud O, et al. Depressed, anxious, and stressed: What have healthcare workers on the frontlines in Egypt and Saudi Arabia experienced during the COVID-19 pandemic? *Journal of Affective Disorders* 2021b;278:365 - 71. doi: 10.1016/j.jad.2020.09.080
17. Aslan I, Ochnik D, Çınar O. Exploring perceived stress among students in Turkey during the covid-19 pandemic. *Front Public Health* 2020;17(23):8961. doi: 10.3390/ijerph17238961
18. Nigusie Shifera Aylie MAM, and Rahel Matiyas Mekuria. The Psychological Impacts of COVID-19 Pandemic Among University Students in Bench-Sheko Zone, South-west Ethiopia: A Community-based Cross-sectional Study. *Psychology Research and Behavior Management* 2020;13:813-21. doi: 10.2147/PRBM.S275593
19. Bachilo EV, Barylnik JB, Shuldyakov AA, et al. Mental Health of Medical Workers During the COVID-19 Pandemic in Russia: Results of a Cross-Sectional Study: Psychiatry and Clinical Psychology, 2020.
20. Badellino H, Gobbo ME, Torres E, et al. Early indicators and risk factors associated with mental health problems during COVID-19 quarantine: Is there a relationship with the number of confirmed cases and deaths? *The International Journal of Social Psychiatry* 2020;67:567-75. doi: 10.1177/0020764020966020
21. Badellino H, Gobbo ME, Torres E, et al. 'It's the economy, stupid': Lessons of a longitudinal study of depression in Argentina. *The International journal of social psychiatry* in press:1-8. doi: 10.1177/0020764021999687
22. Balay-odao EM, Alquwez N, Inocian EP, et al. Hospital Preparedness, Resilience, and Psychological Burden Among Clinical Nurses in Addressing the COVID-19 Crisis in Riyadh, Saudi Arabia. *Front Public Health* 2021;8 doi: 10.3389/fpubh.2020.573932
23. Barua L, Zaman MS, Omi FR, et al. Psychological burden of the COVID-19 pandemic and its associated factors among frontline doctors of Bangladesh: a cross-sectional study. *Journal of Public Health-Heidelberg* 2021;9:1304. doi: 10.12688/f1000research.27189.2
24. Ben-Ezra M, Sun S, Hou WK, et al. The association of being in quarantine and related COVID-19 recommended and non-recommended behaviors with psychological distress in Chinese population. 2020;275:66-68. doi: 10.1016/j.jad.2020.06.026
25. Biswas S, Biswas A. Anxiety level among students of different college and universities in India during lock down in connection to the COVID-19 pandemic. *Frontiers in Psychology* 2021;11 doi: 10.1007/s10389-020-01431-8
26. Boluarte-Carbajal A, Navarro-Flores A, Villarreal-Zegarra D. Explanatory Model of Perceived Stress in the General Population: A Cross-Sectional Study in Peru

During the COVID-19 Context. *Frontiers in Psychology* 2021;12:673945. doi: 10.3389/fpsyg.2021.673945

27. Brito-Marques J, Franco CMR, de Brito-Marques PR, et al. Impact of COVID-19 pandemic on the sleep quality of medical professionals in Brazil. *Arquivos De Neuro-Psiquiatria* 2021;79(2):149-55. doi: 10.1590/0004-282X-anp-2020-0449
28. Cai Z, Cui Q, Liu Z, et al. Nurses endured high risks of psychological problems under the epidemic of COVID-19 in a longitudinal study in Wuhan China. *Journal of Psychiatric Research*;131:132-37. doi: https://doi.org/10.1016/j.jpsychires.2020.09.007
29. Campos JADB, Martins BG, Campos LA, et al. Early Psychological Impact of the COVID-19 Pandemic in Brazil: A National Survey. *Journal of Clinical Medicine* 2020;9(9):2976. doi: 10.3390/jcm9092976
30. Campos J, Campos LA, Bueno JL, et al. Emotions and mood swings of pharmacy students in the context of the coronavirus disease of 2019 pandemic. *Currents in pharmacy teaching & learning* 2021;13(6):635-42. doi: 10.1016/j.cptl.2021.01.034 [published Online First: 2021/04/20]
31. Campos J, Martins BG, Campos LA, et al. Symptoms related to mental disorder in healthcare workers during the COVID-19 pandemic in Brazil. *International archives of occupational and environmental health* 2021;94(5):1023-32. doi: 10.1007/s00420-021-01656-4 [published Online First: 2021/02/10]
32. Cao J, Wei J, Zhu H, et al. A Study of Basic Needs and Psychological Wellbeing of Medical Workers in the Fever Clinic of a Tertiary General Hospital in Beijing during the COVID-19 Outbreak. *Psychother Psychosom* 2020;89(4):252-54. doi: 10.1159/000507453 [published Online First: 2020/04/01]
33. Caycho-Rodriguez T, Tomas JM, Vilca LW, et al. Predictors of mental health during the COVID-19 pandemic in older adults: the role of socio-demographic variables and COVID-19 anxiety. *Psychology Health & Medicine* in press doi: 10.1080/13548506.2021.1944655 [published Online First: 22 Jun 2021]
34. Cayo-Rojas CF, Castro-Mena MJ, Agramonte-Rosell RC, et al. Impact of COVID-19 Mandatory Social Isolation on the Development of Anxiety in Peruvian Dentistry Students: A Logistic Regression Analysis. *Journal of International Society of Preventive & Community Dentistry* 2021;11(2):222-29. doi: 10.4103/jispcd.JISPCD_52_21 [published Online First: 2021/05/27]
35. Cénat JM, Dalexis RD, Guerrier M, et al. Frequency and correlates of anxiety symptoms during the COVID-19 pandemic in low-and middle-income countries: A multinational study. *Journal of Psychiatric Research* 2021;132:13-17. doi: 10.1016/j.jpsychires.2020.09.031
36. Chatterjee KCaM. Psychological impact of COVID-19 pandemic on general population in West Bengal: A cross-sectional study. *BJPsych Open* 2020;62(3):266 - 72. doi: 10.4103/psychiatry.IndianJPsychiatry_276_20
37. Chatterjee SS, Bhattacharyya R, Bhattacharyya S, et al. Attitude, practice, behavior, and mental health impact of COVID 19 on doctors. *Brain// Behavior// and Immunity* 2020;62(3):257. doi: 10.4103/psychiatry.IndianJPsychiatry_333_20
38. Chen X, Zhang SX, Jahanshahi AA, et al. Belief in Conspiracy Theory about COVID-19 Predicts Mental Health and Well-being – A Study of Healthcare Staff in Ecuador. *JMIR Public Health and Surveillance* 2020;6(3):e20737. doi: 10.2196/20737

39. Chen Y, Zhou H, Zhou Y, et al. Prevalence of self-reported depression and anxiety among pediatric medical staff members during the COVID-19 outbreak in Guiyang, China. *Psychiatry Res* 2020;288:113005. doi: 10.1016/j.psychres.2020.113005 [published Online First: 2020/04/22]
40. Chen H, Zhao X, Zeng M, et al. Collective self-esteem and perceived stress among the non-infected general public in China during the 2019 coronavirus pandemic: A multiple mediation model. *ScienceDirect* 2021;168:110308. doi: https://doi.org/10.1016/j.paid.2020.110308
41. Chen B, Li QX, Zhang H, et al. The psychological impact of COVID-19 outbreak on medical staff and the general public. *Current Psychology* 2020:1-9. doi: doi.org/10.1007/s12144-020-01109-0
42. Chen H, Wang B, Cheng Y, et al. Prevalence of posttraumatic stress symptoms in health care workers after exposure to patients with COVID-19. *Neurobiology of Stress* 2020;13:100261. doi: doi.org/10.1016/j.ynstr.2020.100261
43. Chen J, Liu X, Wang D, et al. Risk factors for depression and anxiety in healthcare workers deployed during the COVID-19 outbreak in China. *Social Pscyhiatry and Psychiatric Epidemiology* 2020;10:10.
44. Cheng FF, Zhan SH, Xie AW, et al. Anxiety in Chinese pediatric medical staff during the outbreak of Coronavirus Disease 2019: a cross-sectional study. *Translational Pediatrics* 2020;9(3):231-36. doi: 10.21037/tp.2020.04.02
45. Chew NWS, Ngiam JN, Tan BY-Q, et al. Asian-Pacific perspective on the psychological well-being of healthcare workers during the evolution of the COVID-19 pandemic. *Kathmandu University Medical Journal* 2020a;6(6) doi: 10.1192/bjo.2020.98
46. Chew NWS, Lee GKH, Tan BYQ, et al. A multinational, multicentre study on the psychological outcomes and associated physical symptoms amongst healthcare workers during COVID- 19 outbreak. *Psychiatrist* 2020b;88: 559 - 65. doi: 10.1016/j.bbi.2020.04.049
47. Choi EPH, Hui BPH, Wan EYF. Depression and Anxiety in Hong Kong during COVID-19. *IJERPH* 2020;17(10):25. doi: https://doi.org/10.3390/ijerph17103740
48. Soon Ken Chow BF, Yit Han Ng, Najmi Naim, Hooi Chin Beh, Mohammad Aizuddin Azizah Ariffin, Mohd Hafyzuddin Md Yusuf, Jia Wen Lee and Ahmad Hatim Sulaiman. Religious Coping, Depression and Anxiety among Healthcare Workers during the COVID-19 Pandemic: A Malaysian Perspective. *Healthcare* 2020;9:79. doi: doi.org/10.3390/healthcare9010079
49. Civantos AM, Bertelli A, Gonçalves A, et al. Mental health among head and neck surgeons in Brazil during the COVID-19 pandemic: A national study. *American Journal of Otolaryngology* 2020;41(6):102694. doi: 10.1016/j.amjoto.2020.102694
50. Cortés-Álvarez NY, Pineiro-Lamas R, Vuelvas-Olmos CR. Psychological effects and associated factors of COVID-19 in a Mexican sample. *Journal Disaster medicine public health preparedness* 2020;14(3):413-24. doi: 10.1017/dmp.2020.215
51. Cypryańska M, Nezlek JB. Anxiety as a mediator of relationships between perceptions of the threat of COVID-19 and coping behaviors during the onset of the pandemic in Poland. *PLOS ONE* 2020;15(10):e0241464. doi: 10.1371/journal.pone.0241464

52. da Silva Júnior AE, de Lima Macena M, de Oliveira ADS, et al. Racial Differences in Generalized Anxiety Disorder During the COVID-19 Pandemic among Brazilian University Students: a National Survey. *Journal of racial and ethnic health disparities* 2021 doi: 10.1007/s40615-021-01107-3 [published Online First: 21 July 2021]
53. Dai Y, Hu, G., Xiong, H., Qiu, H., & Yuan, X. Psychological impact of the coronavirus disease 2019 (COVID-19) outbreak on healthcare workers in China. 2020 doi: 10.1101/2020.03.03.20030874
54. Dal'Bosco EB, Floriano LSM, Skupien SV, et al. Mental health of nursing in coping with COVID-19 at a regional university hospital. *Journal Revista brasileira de enfermagem* 2020;73:e20200434. doi: 10.1590/0034-7167-2020-0434
55. Dangal MR, Bajracharya LS. Students anxiety experiences during COVID-19 in Nepal. *Journal of Mental Health Training Education and Practice* 2020;18(2):53 - 57. doi: 10.3126/kumj.v18i2.32957
56. Dantas ESO, Araújo Filho JD, Silva G, et al. Factors associated with anxiety in multiprofessional health care residents during the COVID-19 pandemic. *Revista brasileira de enfermagem* 2021;74S(Suppl 1):e20200961. doi: 10.1590/0034-7167-2020-0961 [published Online First: 2021/04/23]
57. Das A, MD, Sil A, et al. A Study to Evaluate Depression and Perceived Stress Among Frontline Indian Doctors Combating the COVID-19 Pandemic. *Cranio* 2020 doi: doi.org/10.4088/PCC.20m02716
58. De Boni RB, Balanzá-Martínez V, Mota JC, et al. Depression, Anxiety, and Lifestyle Among Essential Workers: A Web Survey From Brazil and Spain During the COVID-19 Pandemic. *Journal of medical Internet research* 2020;22(10):e22835. doi: 10.2196/22835
59. Cheng-Hu Deng J-QW, Li-Ming Zhu, He-Wang Liu, Yu Guo, Xue-Hua Peng, Jian-Bo Shao, Wei Xia. Association of Web-Based Physical Education With Mental Health of College Students in Wuhan During the COVID-19 Outbreak: Cross-Sectional Survey Study. *Journal of Medical Internet Research* 2020;22(10)
60. Dong Z-Q, Ma J, Hao Y-N, et al. The social psychological impact of the COVID-19 pandemic on medical staff in China: A cross-sectional study. *European Psychiatry* 2020;63(1)
61. Du J, Dong L, Wang T, et al. Psychological symptoms among frontline healthcare workers during COVID-19 outbreak in Wuhan. *Gen Hosp Psychiatry* 2020 doi: 10.1016/j.genhosppsych.2020.03.011 [published Online First: 2020/05/10]
62. Do Duy C, Nong VM, Van AN, et al. COVID-19 related stigma and its association with mental health of health-care workers after quarantined in Vietnam. *Psychiatry and Clinical Neurosciences* 2020
63. Dzhambov AM, Lercher P, Browning MHEM, et al. Does greenery experienced indoors and outdoors provide an escape and support mental health during the COVID-19 quarantine? *Environmental Research* 2020:110420. doi: 10.1016/j.envres.2020.110420
64. Fatime Elezi GT, Eugjen Sotiri, Herta Agaj, and Kristi Kola. Assessment of anxiety and depression symptoms in the Albanian general population during the outbreak of COVID-19 pandemic. *Indian Journal Psychiatry* 2020;62:470-75. doi: 10.4103/psychiatry.IndianJPsychiatry_842_20
65. Muhammed Elhadi AM, Moutaz Elgzairi, Ayiman Alhashimi, Ahmad Bouhuwaish, Marwa Biala, Seraj Abuelmed. Psychological status of healthcare workers

during the civil war and COVID-19 pandemic: A cross-sectional study. *Journal of Psychosomatic Research* 2020;137 doi: doi.org/10.1016/j.jpsychores.2020.110221

66. Muhammed Elhadi AB, Ahmad Bouhuwaish, Ala Khaled, Abdulmueti Alhadi, Ahmed Msherghi. Psychological Impact of the Civil War and COVID-19 on Libyan Medical Students: A Cross-Sectional Study. *Frontiers in Psychology* 2020 doi: doi.org/10.3389/fpsyg.2020.570435
67. Muhammed Elhadi AM, Moutaz Elgzairi, Ayiman Alhashimi, Ahmad Bouhuwaish, Marwa Biala. The Mental Well-Being of Frontline Physicians Working in Civil Wars Under Coronavirus Disease 2019 Pandemic Conditions. *Frontiers in Psychology* 2020;11 doi: 10.3389/fpsyt.2020.598720
68. Elhai JD, Yang H, McKay D, et al. COVID-19 anxiety symptoms associated with problematic smartphone use severity in Chinese adults. *Journal of Affective Disorders* 2020;274:576-82. doi: doi.org/10.1016/j.jad.2020.05.080
69. Hussein Elkholy FT, Islam Ibrahim, Waleed Salah El-din, Mohamed Sabry, Suzan Mohammed, Mohamed Hamza,. Mental health of frontline healthcare workers exposed to COVID-19 in Egypt: A call for action. *International Journal of Social Psychiatry* 2020 doi: doi.org/10.1177/0020764020960192
70. Espinosa-Guerra EA, Rodríguez-Barría ER, Donnelly CA, et al. Prevalence and associated factors with mental health outcomes among interns and residents physicians during COVID-19 epidemic in Panama: a cross-sectional study. *medRxiv* 2021:21254435. doi: 10.1101/2021.03.26.21254435 [published Online First: 2021.03.26]
71. Predictive factors for impaired mental health among medical students during the early stage of the COVID-19 pandemic in Morocco. *The American Journal of Tropical Medicine and Hygiene* 2021;104(1):95-102. doi: 10.4269/ajtmh.20-1302
72. Esteves CS, de Oliveira CR, Argimon IID. Social Distancing: Prevalence of Depressive, Anxiety, and Stress Symptoms Among Brazilian Students During the COVID-19 Pandemic. *Frontiers in Public Health* 2021;8:5. doi: 10.3389/fpubh.2020.589966
73. Faize FA, Husain W. Students with severe anxiety during COVID-19 lockdown - exploring the impact and its management. *JMIR PUBLIC HEALTH AND SURVEILLANCE* 2021;16(2):153 - 63. doi: 10.1108/jmhtep-09-2020-0062
74. Fang X, Zhang J, Teng C, et al. Depressive symptoms in the front-line non-medical workers during the COVID-19 outbreak in Wuhan. *Journal of Affective Disorders* 2020;276:441-45. doi: doi.org/10.1016/j.jad.2020.06.078
75. Mohd Fauzi MF, Mohd Yusoff H, Muhamad Robat R, et al. Doctors' Mental Health in the Midst of COVID-19 Pandemic: The Roles of Work Demands and Recovery Experiences. *International Journal of Environmental Research and Public Health* 2020;17(19):7340.
76. Feng Z, Xu L, Cheng P, et al. The psychological impact of COVID-19 on the families of first-line rescuers. *Indian Journal of Pscyhiatry* 2020;62(Suppl 3):S438. doi: 10.4103/psychiatry.IndianJPsychiatry_1057_20
77. Fernández RS, Crivelli L, Guimet NM, et al. Psychological distress associated with COVID-19 quarantine: Latent profile analysis, outcome prediction and mediation analysis. *Journal of affective disorders* 2020;277:75-84. doi: https://doi.org/10.1016/j.jad.2020.07.134

78. Fernandez S, Vieira IS, Silva N, et al. Anxiety symptoms and alcohol abuse during the COVID-19 pandemic: A cross-sectional study with Brazilian dental undergraduate students. *Journal of dental education* 2021;2021:1-10. doi: 10.1002/jdd.12742 [published Online First: 2021/07/17]
79. Ferreira FD, Lopes-Silva JB, Siquara GM, et al. Coping in the Covid-19 pandemia: how different resources and strategies can be risk or protective factors to mental health in the Brazilian nopulation. *Health Psychology and Behavioral Medicine* 2021;9(1):182-205. doi: 10.1080/21642850.2021.1897595
80. Feter N, Caputo E, Doring I, et al. Sharp increase in depression and anxiety among Brazilian adults during the COVID-19 pandemic: findings from the PAMPA cohort. *Public health* 2021;190:101-07. doi: 10.1016/j.puhe.2020.11.013
81. Flores-Torres MH, Murchland AR, Espinosa-Tamez P, et al. Prevalence and Correlates of Mental Health Outcomes During the SARS-Cov-2 Epidemic in Mexico City and Their Association With Non-adherence to Stay-At-Home Directives, June 2020. *International Journal of Public Health* 2021;66:1-10. doi: 10.3389/ijph.2021.620825
82. Fong BY, Wong M, Law VT, et al. Relationships between Physical and Social Behavioural Changes and the Mental Status of Homebound Residents in Hong Kong during the COVID-19 Pandemic. *International Journal of Environmental Research and Public Health* 2020;17(18):6653. doi: doi.org/10.3390/ijerph17186653
83. Fu W, Wang C, Zou L, et al. Psychological health, sleep quality, and coping styles to stress facing the COVID-19 in Wuhan, China. *Translational Psychiatry* 2020;10(1):1-9. doi: doi.org/10.1038/s41398-020-00913-3
84. Gallopeni F, Bajraktari I, Selmani E, et al. Anxiety and depressive symptoms among healthcare professionals during the Covid-19 pandemic in Kosovo: A cross sectional study. *Journal of Psychosomatic Research* 2020;137:110212. doi: 10.1016/j.jpsychores.2020.110212
85. Gao J, Zheng P, Jia Y, et al. Mental health problems and social media exposure during COVID-19 outbreak. *PLoS ONE* 2020;15(4):e0231924. doi: https://doi.org/10.1371/journal.pone.0231924
86. García-Espinosa P, Ortiz-Jiménez X, Botello-Hernández E, et al. Psychosocial impact on health-related and non-health related university students during the COVID-19 pandemic. Results of an electronic survey. *Revista Colombiana de Psiquiatria* 2021;50(3):214-24. doi: 10.1016/j.rcp.2021.04.008
87. Gaş S, Özsoy HE, Aydın KC. The association between sleep quality, depression, anxiety and stress levels, and temporomandibular joint disorders among Turkish dental students during the COVID-19 pandemic. *Asian Journal of Psychiatry* 2021:6. doi: 10.1080/08869634.2021.1883364
88. Fenfen Ge DZ, Lianhai Wu, Hongwei Mu. Predicting Psychological State Among Chinese Undergraduate Students in the COVID-19 Epidemic: A Longitudinal Study Using a Machine Learning. *Neuropsychiatric Disease and Treatment* 2020;16:2111-18. doi: 10.2147/NDT.S262004
89. Giardino DL, Huck-Iriart C, Riddick M, et al. The endless quarantine: The impact of the COVID-19 outbreak on healthcare workers after three months of mandatory social isolation in Argentina. *Sleep medicine* 2020;76:16-25. doi: 10.1016/j.sleep.2020.09.022

90. Goularte JF, Serafim SD, Colombo R, et al. COVID-19 and mental health in Brazil: Psychiatric symptoms in the general population. *Journal of psychiatric research* 2021;132:32-37. doi: 10.1016/j.jpsychires.2020.09.021
91. Guiroy A, Gagliardi Mı, Coombes N, et al. COVID-19 Impact Among Spine Surgeons in Latin America. *Global Spine Journal* 2021;11(6):859-65. doi: doi: 10.1177/2192568220928032
92. Guo J, Liao, L., Wang, B., Li, X., Guo, L., Tong, Z., ... Gu, Y. Psychological effects of COVID-19 on hospital staff: a national cross-sectional survey of China mainland. *The Lancet* 2020;4(1):6-11. doi: 10.4103/VIT-2
93. Guo J, Feng XL, Wang XH, et al. Coping with COVID-19: Exposure to COVID-19 and Negative Impact on Livelihood Predict Elevated Mental Health Problems in Chinese Adults. *International Journal of Environmental Research and Public Health* 2020;17(11):3857. doi: doi.org/10.3390/ijerph17113857
94. Gupta B, Sharma V, Kumar N, et al. Anxiety and Sleep Disturbances Among Health Care Workers During the COVID-19 Pandemic in India: Cross-Sectional Online Survey. *Indian J Psychol Med* 2020a;6(4) doi: 10.2196/24206
95. Gupta AK, Mehra A, Niraula A, et al. Prevalence of anxiety and depression among the healthcare workers in Nepal during the COVID-19 pandemic. *medical journal armed for cesindia* 2020b;54 doi: 10.1016/j.ajp.2020.102260
96. Gupta S, Kohli K, Padmakumari P, et al. Psychological Health among armed Forces Doctors During COVID-19 Pandemic in India. *Canadian Psychology/Psychologie canadienne* 2020c;42(4):374 - 78. doi: 10.1177/0253717620934037
97. Gupta S, Prasad AS, Dixit PK, et al. Survey of prevalence of anxiety and depressive symptoms among 1124 healthcare workers during the coronavirus disease 2019 pandemic across India. *Pakistan Journal of Medical Sciences* 2021d;77:S404 - S12. doi: 10.1016/j.mjafi.2020.07.006
98. Han L, Wong FKY, She DLM, et al. Anxiety and Depression of Nurses in a North West Province in China During the Period of Novel Coronavirus Pneumonia Outbreak. *J Nurs Scholarsh* 2020;Journal of Nursing Scholarship doi: 10.1111/jnu.12590 [published Online First: 2020/07/12]
99. Hasan SR, Hamid Z, Jawaid MT, et al. Anxiety among Doctors during COVID-19 Pandemic in Secondary and Tertiary Care Hospitals. *Postgrad Med J* 2020;36(6) doi: 10.12669/pjms.36.6.3113
100. Kholisotul Hikmah LP, Gea Melinda, M. Ibraar Ayatullah. An Online Survey: Assessing Anxiety Level among General Population during the Coronavirus Disease-19 Pandemic in Indonesia. *Macedonian Journal of Medical Sciences* 2020;8 doi: doi.org/10.3889/oamjms.2020.5386
101. Hong S, Ai M, Xu X, et al. Immediate psychological impact on nurses working at 42 government-designated hospitals during COVID-19 outbreak in China: A cross-sectional study. *Nursing Outlook* 2020;69(1):6-12. doi: doi.org/10.1016/j.outlook.2020.07.007
102. Hossain MT, Ahammed B, Chanda SK, et al. Social and electronic media exposure and generalized anxiety disorder among people during COVID-19 outbreak in Bangladesh: A preliminary observation. *Annals of King Edward Medical University* 2020;15(9) doi: 10.1371/journal.pone.0238974
103. Hou F, Bi F, Jiao R, et al. Gender differences of depression and anxiety among social media users during the COVID-19 outbreak in China: a cross-sectional

study. *BMC Public Health* 2020;20(1):1-11. doi: doi.org/10.1186/s12889-020-09738-7

104. Hu N, Li Y, He S-S, et al. Impact of the Family Environment on the Emotional State of Medical Staff During the COVID-19 Outbreak: The Mediating Effect of Self-Efficacy. *Frontiers in Psychology* 2020;11 doi: 10.3389/fpsyg.2020.576515
105. Hu D, Kong Y, Li W, et al. Frontline Nurses' Burnout, Anxiety, Depression, and Fear Statuses and Their Associated Factors During the COVID-19 Outbreak in Wuhan, China: A Big-Scale Cross-Sectional Study. *The Lancet* 2020;24 doi: doi.org/10.1016/j.eclinm.2020.100424
106. Public behavior change, perceptions, depression, and anxiety in relation to the COVID-19 outbreak. Open Forum Infectious Diseases; 2020. Oxford University Press US.
107. Huang L, Wang Y, Liu J, et al. Factors influencing anxiety of health care workers in the radiology department with high exposure risk to COVID-19. *Medical Science Monitor* 2020;26:e926008-1. doi: 10.12659/MSM.926008
108. Huang Y, Wang Y, Zeng L, et al. Prevalence and Correlation of Anxiety, Insomnia and Somatic Symptoms in a Chinese Population During the COVID-19 Epidemic. *Frontiers in Psychology* 2020;11:894. doi: doi.org/10.3389/fpsyt.2020.568329
109. Abdelkrim Janati Idrissi AL, Abdelilah Benouajjit, Manar Ben El Bouaazzaoui, Farah El Houari, Mohammed Alami. Sleep quality and mental health in the context of COVID-19 pandemic and lockdown in Morocco *Sleep Medicine* 2020;74:248-53. doi: doi.org/10.1016/j.sleep.2020.07.045
110. Imran N, Masood HMU, Ayub M, et al. Psychological impact of COVID-19 pandemic on postgraduate trainees: a cross-sectional survey. *Plos One* 2020a;97(1152) doi: 10.1136/postgradmedj-2020-138364
111. Imran N, Hashmi AM, Haider II, et al. The Toll it Takes: Mental Health Burden and Associated Factors During COVID-19 Outbreak among Healthcare Workers in Lahore, Pakistan. *Indian Journal of Anaesthesia* 2020b;26(2)
112. Islam MA, Barna SD, Raihan H, et al. Depression and anxiety among university students during the COVID-19 pandemic in Bangladesh: A web-based cross-sectional survey. *Advances in respiratory medicine* 2020;15(8) doi: 10.1371/journal.pone.0238162
113. Jain A, Singariya G, Kamal M, et al. COVID-19 pandemic: Psychological impact on anaesthesiologists. *Annals of Vascular Surgery* 2020;64(9):774. doi: 10.4103/ija.IJA_697_20
114. Yuehui Jia YQ, Li Bai, Yunfeng Han, Zhiping Xie, Jie Ge. Knowledge-attitude-practice and psychological status of college students during the early stage of COVID-19 outbreak in China: a cross-sectional study. *BMJ Open* 2021;11(2) doi: doi.org/10.1136/bmjopen-2020-045034
115. Jin Z, Zhao K-b, Xia Y-y, et al. Relationship Between Psychological Responses and the Appraisal of Risk Communication During the Early Phase of the COVID-19 Pandemic: A Two-Wave Study of Community Residents in China. *Frontiers in Psychology* 2020;8 doi: doi.org/10.3389/fpubh.2020.550220
116. Jindal V, Mittal S, Kaur T, et al. Knowledge, anxiety and the use of hydroxychloroquine prophylaxis among health care students and professionals regarding COVID-19 pandemic. *Globalization and Health* 2020;88(6):520 - 30. doi: 10.5603/ARM.a2020.0163

117. Juan Y, Yuanyuan C, Qiuxiang Y, et al. Psychological distress surveillance and related impact analysis of hospital staff during the COVID-19 epidemic in Chongqing, China. *Comprehensive Psychiatry* 2020;103:152198. doi: doi.org/10.1016/j.comppsych.2020.152198
118. Kafle K, Shrestha DB, Baniya A, et al. Psychological distress among health service providers during COVID-19 pandemic in Nepal. *Frontiers in Psychiatry* 2021;16(2) doi: 10.1371/journal.pone.0246784
119. Aida Kalok SS, Abdul Muzhill Abdul Hafizz, Zulkifli Zainuddin, Mohamad Nasir Shafiee. The psychological impact of movement restriction during the COVID-19 outbreak on clinical undergraduates: A cross-sectional study. *International Journal of Environmental Research and Public Health* 2020;17(22) doi: doi.org/10.3390/ijerph17228522
120. Kamaludin K, Chinna K, Sundarasen S, et al. Coping with COVID-19 and movement control order (MCO): experiences of university students in Malaysia. *Heliyon* 2020;6(11):e05339.
121. Karpenko OA, Syunyakov TS, Kulygina MA, et al. Impact of COVID-19 pandemic on anxiety, depression and distress – online survey results amid the pandemic in Russia. *Consortium Psychiatricum* 2020;1(1):8-20. doi: 10.17650/2712-7672-2020-1-1-8-20
122. Kassaw C. The Magnitude of Psychological Problem and Associated Factor in Response to COVID-19 Pandemic Among Communities Living in Addis Ababa, Ethiopia, March 2020: A Cross-Sectional Study Design. *Psychology Research and Behavior Management* 2020;13:631-40. doi: 10.2147/PRBM.S256551
123. François Roger Nguépy Keubo PCM, Thomas Djifack Tadongfack, Eugène Fokouong Tchoffo, Cyrille Tasson Tatang, Julienne Ide Zeuna. Psychological distress among health care professionals of the three COVID-19 most affected Regions in Cameroon: Prevalence and associated factors. *Annales Médico-psychologiques revue psychiatrique* 2021;179(2):141-46. doi: doi.org/10.1016/j.amp.2020.08.012
124. Khan AH, Sultana MS, Hossain S, et al. The impact of COVID-19 pandemic on mental health & wellbeing among home-quarantined Bangladeshi students: A cross-sectional pilot study. *Journal of PeriAnesthesia Nursing* 2020;277:121 - 28. doi: 10.1016/j.jad.2020.07.135
125. Khanal P, Devkota N, Dahal M, et al. Mental health impacts among health workers during COVID-19 in a low resource setting: a cross-sectional survey from Nepal. *JOEM* 2020a;16(1) doi: 10.1186/s12992-020-00621-z
126. Khanna R, Honavar S, Metla A, et al. Psychological impact of COVID-19 on ophthalmologists-in-training and practising ophthalmologists in India. *The Open Dentistry Journal* 2020b;68(6):994. doi: 10.4103/psychiatry.IndianJPsychiatry_276_20
127. Khatun MF, Parvin MF, Rashid MM-u, et al. Mental Health of Physicians During COVID-19 Outbreak in Bangladesh: A Web-Based Cross-Sectional Survey. *Journal of Indian Association for Child and Adolescent Mental Health* 2021;9 doi: 10.3389/fpubh.2021.592058
128. Khoshaim HB, Al-Sukayt A, Chinna K, et al. Anxiety Level of University Students During COVID-19 in Saudi Arabia. *Indian Journal of Ophthalmology* 2020;11 doi: 10.3389/fpsyt.2020.579750

129. Sim Sze Kiat LBL, Siti Rosnaini Zaila Ramli, Sijil Matrikulasi, Nur Hazira Abdul Muti, Sijil Asasi Sains Hayat Unimas. Psychological symptoms among healthcare workers handling COVID-19 patients. 2021
130. Simegnew Kibret DT, Efrem Fenta, Metages Hunie, Tadese Tamire. Prevalence of anxiety towards COVID-19 and its associated factors among healthcare workers in a Hospital of Ethiopia. *PLOS ONE* 2020 doi: doi.org/10.1371/journal.pone.0243022
131. Andrew Wooyoung Kim TN, Emily Mendenhall. Evaluating the Mental Health Impacts of the COVID-19 Pandemic in Urban South Africa: Perceived Risk of COVID-19 Infection and Childhood Trauma Predict Adult Depressive Symptoms. *Psychology Medicine* 2020:1-13. doi: doi.org/10.1017/S0033291720003414
132. Koksal E, Dost B, Terzi Ö, et al. Evaluation of Depression and Anxiety Levels and Related Factors Among Operating Theater Workers During the Novel Coronavirus (COVID-19) Pandemic. *Brain and Behavior* 2020;35(5):472 - 77. doi: 10.1016/j.jopan.2020.06.017
133. Kounou KB, Guédénon KM, Foli AAD, et al. Mental health of medical professionals during the covid-19 pandemic in Togo. *Psychiatry and Clinical Neurosciences* 2020;74(10):559-60. doi: 10.1111/pcn.13108
134. Kumar D, Saghir T, Ali G, et al. Psychosocial Impact of COVID-19 on Healthcare Workers at a Tertiary Care Cardiac Center of Karachi Pakistan. *International Journal of Research in Pharmaceutical Science* 2021;63(2) doi: 10.1097/JOM.0000000000002094
135. Leodoro J. Labrague JAADlSR. COVID-19 anxiety among front-line nurses: Predictive role of organisational support, personal resilience and social support. *Journal of Nusing Management* 2020;28(7):1653-61. doi: doi.org/10.1111/jonm.13121
136. Lai J, Ma S, Wang Y, et al. Factors Associated With Mental Health Outcomes Among Health Care Workers Exposed to Coronavirus Disease 2019. *JAMA Netw Open* 2020;3(3) doi: 10.1001/jamanetworkopen.2020.3976
137. Lam SC, Arora T, Grey I, et al. Perceived risk and protection from infection and depressive symptoms among healthcare workers in mainland China and Hong Kong during COVID-19. *Frontiers in Psychology* 2020;11:686. doi: doi.org/10.3389/fpsyt.2020.00686
138. Landaeta-Díaz L, González-Medina G, Agüero SD. Anxiety, anhedonia and food consumption during the COVID-19 quarantine in Chile. *Appetite* 2021;164:105259. doi: 10.1016/j.appet.2021.105259 [published Online First: 1 September 2021]
139. Huong Thi Le AJXL, Jiaqian Sun, Men Thi Hoang, Linh Gia Vu, Hai Quang Pham. Anxiety and Depression Among People Under the Nationwide Partial Lockdown in Vietnam. *Frontiers in Psychology* 2020 doi: doi.org/10.3389/fpubh.2020.589359
140. Lei L, Huang X, Zhang S, et al. Comparison of Prevalence and Associated Factors of Anxiety and Depression Among People Affected by versus People Unaffected by Quarantine During the COVID-19 Epidemic in Southwestern China. *Med Sci Monit* 2020;26:e924609. doi: 10.12659/MSM.924609 [published Online First: 2020/04/27]

141. Leng M, Wei L, Shi X, et al. Mental distress and influencing factors in nurses caring for patients with COVID-19. *Nursing in Critical Care* 2020;26(2):94-101. doi: doi.org/10.1111/nicc.12528
142. Li QJQAIJoM. Psychosocial and coping responses toward 2019 coronavirus diseases (COVID-19): a cross-sectional study within the Chinese general population. *An International Jounral of Medicine* 2020;113(10):731-38. doi: doi.org/10.1093/qjmed/hcaa226
143. Li X, Yu, H., Bian, G., Hu, Z., Liu, X., Zhou, Q., . . . Zhou, D. Prevalence, risk factors, and clinical correlates of insomnia in volunteer and at home medical staff during the COVID-19. *Brain, Behavior, and Immunity* 2020;87:140-41. doi: 10.1016/j.bbi.2020.05.008
144. Li G, Miao, J, Wang, H, Xu, S, Sun, W, Fan, Y, et al. Psychological impact on women health workers involved in COVID-19 outbreak in Wuhan: a cross-sectional study. *BMJ Jounrals*;91(8) doi: dx.doi.org/10.1136/jnnp-2020-323134
145. Li Y, Qin Q, Sun Q, et al. Insomnia and psychological reactions during the COVID-19 outbreak in China. *J Clin Sleep Med* 2020;16(8):1417-18. doi: 10.5664/jcsm.8524 [published Online First: 2020/05/01]
146. Mengyao Li LL, Yilong Yang, Yang Wang, Xiaoshi Yang, Hui Wu. Psychological Impact of Health Risk Communication and Social Media on College Students. *Journal of Medical Internet Research* 2020;22(11) doi: preprints.jmir.org/preprint/20656
147. Li Q, Chen J, Xu G, et al. The Psychological Health Status of Healthcare Workers During the COVID-19 Outbreak: A Cross-Sectional Survey Study in Guangdong, China. *Frontiers in Psychology* 2020;8:572. doi: doi.org/10.3389/fpubh.2020.562885
148. Li Q, Miao Y, Zeng X, et al. Prevalence and factors for anxiety during the coronavirus disease 2019 (COVID-19) epidemic among the teachers in China. *Journal of Affective Disorders* 2020;277:153-58. doi: doi.org/10.1016/j.jad.2020.08.017
149. Li R, Chen Y, Lv J, et al. Anxiety and related factors in frontline clinical nurses fighting COVID-19 in Wuhan. *Medicine* 2020;99(30) doi: 10.1097/MD.0000000000021413
150. Li X, Li S, Xiang M, et al. The prevalence and risk factors of PTSD symptoms among medical assistance workers during the COVID-19 pandemic. *Journal of Psychosomatic Research* 2020;139:110270. doi: doi.org/10.1016/j.jpsychores.2020.110270
151. Xiuchuan Li YZ, Xiaoyue Xu. Factors associated with the psychological well-being among front-line nurses exposed to COVID-2019 in China: A predictive study. *Journal of Nusing Management* 2020;29(2):240-49. doi: doi.org/10.1111/jonm.13146
152. Li L, Sun N, Fei S, et al. Current status of and factors influencing anxiety and depression in front-line medical staff supporting Wuhan in containing the novel coronavirus pneumonia epidemic. *Japan Journal of Nursing Science* 2020;18(2):e12398. doi: doi.org/10.1111/jjns.12398
153. Liang Y, Wu K, Zhou Y, et al. Mental health in frontline medical workers during the 2019 novel coronavirus disease epidemic in China: a comparison with the general population. *IJERPH* 2020;17(18):6550. doi: doi.org/10.3390/ijerph17186550

154. Shun-Wei Liang R-NC, Li-Li Liu, Xue-Guo Li, Jian-Bin Chen, Si-Yao Tang. The Psychological Impact of the COVID-19 Epidemic on Guangdong College Students: The Difference Between Seeking and Not Seeking Psychological Help. *Frontiers in Psychology* 2020 doi: doi.org/10.3389/fpsyg.2020.02231
155. Lin K, Yang BX, Luo D, et al. The mental health effects of COVID-19 on health care providers in China. *The American Journal of Psychiatry* 2020;177(7):635-36. doi: doi.org/10.1176/appi.ajp.2020.20040374
156. Lin L-y, Wang J, Ou-yang X-y, et al. The immediate impact of the 2019 novel coronavirus (COVID-19) outbreak on subjective sleep status. *Sleep Medicine* 2020;77:348-54. doi: doi.org/10.1016/j.sleep.2020.05.018
157. Depression is Associated with Moderate-Intensity Physical Activity Among College Students During the COVID-19 Pandemic: Differs by Activity Level, Gender and Gender Role. *Psychology Research and Behavior Management* 2020;13:1123-34. doi: 10.2147/PRBM.S277435
158. Lingawi HS, Afifi IK. COVID-19 associated stress among dental students. *Journal of Obstetrics and Gynecology of India* 2020;14(1):554 - 62. doi: 10.2174/1874210602014010554
159. Liu C-Y, Yang, Y.-z., Zhang, X.-M., Xu, X., Dou, Q.-L., Zhang, W.-W., & Cheng, A. S. K. The prevalence and influencing factors in anxiety in medical workers fighting COVID-19 in China: a cross-sectional survey. *Epidemiology and Infection* 2020;148 doi: 10.1017/s0950268820001107
160. Liu D, Ren Y, Yan F, et al. Psychological Impact and Predisposing Factors of the Coronavirus Disease 2019 (COVID-19) Pandemic on General Public in China. *Lancet Psychiatry* in press doi: http://dx.doi.org/10.2139/ssrn.3551415
161. Liu N, Zhang F, Wei C, et al. Prevalence and predictors of PTSS during COVID-19 outbreak in China hardest-hit areas: Gender differences matter. *Psychiatry Res* 2020;287:112921. doi: 10.1016/j.psychres.2020.112921 [published Online First: 2020/04/03]
162. Liu Z, Han B, Jiang RM, et al. Mental health status of doctors and nurses during COVID-19 epidemic in China. *Preprint with The Lancet, SSRN* 2020
163. Liu M, Zhang H, Huang HJBPH. Media exposure to COVID-19 information, risk perception, social and geographical proximity, and self-rated anxiety in China. *BMC Public Health* 2020;20(1):1-8.
164. Liu X, Luo W-T, Li Y, et al. Psychological status and behavior changes of the public during the COVID-19 epidemic in China. *Infectious Diseases of Poverty* 2020;9(1):1-11. doi: doi.org/10.
1186/s40249-020-00678-3
165. Liu Y, Chen H, Zhang N, et al. Anxiety and depression symptoms of medical staff under COVID-19 epidemic in China. *Journal of Affective Disorders* 2020;278:144-48. doi: doi.org/10.1016/j.jad.2020.09.004
166. Liu Y, Wang L, Chen L, et al. Mental health status of paediatric medical workers in China during the COVID-19 outbreak. *Frontiers in Psychology* 2020;11 doi: 10.3389/fpsyt.2020.00702
167. Lu W, Wang H, Lin Y, et al. Psychological status of medical workforce during the COVID-19 pandemic: A cross-sectional study. *Psychiatry Research* 2020;288 doi: 10.1016/j.psychres.2020.112936
168. Lu H, Nie P, Qian LJARiQoL. Do quarantine experiences and attitudes towards COVID-19 affect the distribution of mental health in China? A quantile

regression analysis. *Applied Research in Quality of Life* 2021;16:1925-42. doi: doi.org/10.1007/s11482-020-09851-0
169. Lu P, Li X, Lu L, et al. The psychological states of people after Wuhan eased the lockdown. *PLOS ONE* 2020;15(11):e0241173. doi: doi.org/10.1371/journal.pone.0241173
170. Z. Ma JZ, Y. Li, D. Chen, T. Wang, Z. Zhang. Mental health problems and correlates among 746 217 college students during the coronavirus disease 2019 outbreak in China. *Epidemiology and Psychiatric Sciences* 2020;29:e181. doi: doi.org/10.1017/S2045796020000931
171. Maciaszek J, Ciulkowicz M, Misiak B, et al. Mental Health of Medical and Non-Medical Professionals during the Peak of the COVID-19 Pandemic: A Cross-Sectional Nationwide Study. *Journal of Clinical Medicine* 2020;9(8):2527. doi: 10.3390/jcm9082527
172. Malgor RD, Sobreira ML, Mouawad NJ, et al. Brazilian vascular surgeons experience during the coronavirus (COVID-19) pandemic. *Vascular* 2020;29:451-60. doi: 10.1177/1708538120954961
173. Mamun MA, Sakib N, Gozal D, et al. The COVID-19 pandemic and serious psychological consequences in Bangladesh: A population-based nationwide study. *The Journal of Obstetrics and Gynecology of India* 2021;279:462 - 72. doi: 10.1016/j.jad.2020.10.036
174. Manjareeka M, Pathak M. COVID-19 Lockdown Anxieties: Is student a vulnerable group? *BMC Psychol* 2021;17(1):72 - 80.
175. Markovic I, Nikolovski S, Milojevic S, et al. Public trust and media influence on anxiety and depression levels among skilled workers during the COVID-19 outbreak in Serbia. *Vojnosanitetski pregled* 2020;77(11):1201-09. doi: 10.2298/VSP200713108M
176. Martinez EZ, Silva FM, Morigi TZ, et al. Physical activity in periods of social distancing due to COVID-19: a cross-sectional survey. *Ciência Saúde Coletiva* 2020;25:4157-68. doi: 10.1590/1413-812320202510.2.27242020
177. Mautong H, Gallardo-Rumbea JA, Alvarado-Villa GE, et al. Assessment of depression, anxiety and stress levels in the Ecuadorian general population during social isolation due to the COVID-19 outbreak: A cross-sectional study. *BMC Psychiatry* 2021;21(212):1-15. doi: 10.1186/s12888-021-03214-1
178. Mboua CP KF, Fouaka SGN. Anxiety and Depression Associated with the Management of COVID-19 Among Healthcare workers in Cameroon. *L' Evolution Psychiatrique* 2020;86(1):131-39. doi: 10.1016/j.evopsy.2020.11.002
179. Mechili EA, Saliaj A, Kamberi F, et al. Is the mental health of young students and their family members affected during the quarantine period? Evidence from the COVID-19 pandemic in Albania. *Journal of Psychiatric and Mental Health Nursing* 2020 doi: 10.1111/jpm.12672
180. Medeiros RAD, Vieira DL, Silva EVFD, et al. Prevalence of symptoms of temporomandibular disorders, oral behaviors, anxiety, and depression in Dentistry students during the period of social isolation due to COVID-19. *Journal of Applied Oral Science* 2020;28(e20200445):1-8. doi: 10.1590/1678-7757-2020-0445
181. Mekonen E, Shetie B, Muluneh N. The Psychological Impact of COVID-19 Outbreak on Nurses Working in the Northwest of Amhara Regional State

Referral Hospitals, Northwest Ethiopia. *Psychological Research Behavior Management* 2020;13:1353–64. doi: 10.2147/PRBM.S291446
182. Mendonca VS, Steil A, Gois AFT. Mental health and the COVID-19 pandemic: a study of medical residency training over the years. *Clinics* 2021;76(e2907):1-6. doi: 10.6061/clinics/2021/e2907
183. Mi T, Yang X, Sun S, et al. Mental Health Problems of HIV Healthcare Providers During the COVID-19 Pandemic: The Interactive Effects of Stressors and Coping. 2020:1-10.
184. Mier-Bolio JR, Arroyo-González JM, Baques-Guillén E, et al. COVID-19 y ansiedad en oftalmólogos. *Revista Mexicana de Oftalmologia* 2020;94(6):223-27. doi: 10.24875/RMOE.M20000132
185. Nair A, Mishra D, Gandhi R, et al. The impact of COVID-19 related lockdown on ophthalmology training programs in India ¨C Outcomes of a survey. *Int J Ment Health Addict* 2020;68(6):999. doi: 10.4103/ijo.IJO_1067_20
186. Mongkhon P RC, Awiphan R, Thavorn K, Hutton B, Wongpakaran N. Exposure to Coronavirus Disease-Related Information and Mental Health Problems: A Nationwide Cross-sectional Survey in Thailand. *Europe PMC* 2021;23(2) doi: 10.2196/25363
187. Monterrosa-Castro A, Redondo-Mendoza V, Mercado-Lara M. Psychosocial factors associated with symptoms of generalized anxiety disorder in general practitioners during the COVID-19 pandemic. *Journal of Investigative Medicine* 2020;68(7):1228-34. doi: 10.1136/jim-2020-001456
188. Mora-Magaña I, Lee SA, Maldonado-Castellanos I, et al. Coronaphobia among healthcare professionals in Mexico: A psychometric analysis. *Death Studies* 2020;1808762:1-10. doi: 10.1080/07481187.2020.1808762
189. Mosolova E, Chung S, Sosin D, et al. Stress and Anxiety among Healthcare Workers Associated with COVID-19 Pandemic in Russia. *Psychiatria Danubina* 2020;32(3-4):549-56. doi: 10.24869/psyd.2020.549
190. Mota IA, Oliveira Sobrinho GD, Morais IPS, et al. Impact of COVID-19 on eating habits, physical activity and sleep in Brazilian healthcare professionals. *Arq Neuropsiquiatr* 2021;79(5):429-36. doi: 10.1590/0004-282x-anp-2020-0482 [published Online First: 2021/05/27]
191. Hailu Abera Mulatu MT, Esubalew Woldeyes, Tola Bayisa, Henok Fisseha, Rodas Asrat Kassu. The prevalence of common mental disorders among health care professionals during the COVID-19 pandemic at a tertiary Hospital in East Africa. *Journal of Affective Disorders Reports* 2021;6 doi: doi.org/10.1016/j.jadr.2021.100246
192. Thejini Naidoo AT, Saeeda Paruk. Burnout, anxiety and depression risk in medical doctors working in KwaZulu-Natal Province, South Africa: Evidence from a multi-site study of resource-constrained government hospitals in a generalised HIV epidemic setting. *PLOS ONE* 2020;15(10) doi: doi.org/10.1371/journal.pone.0239753
193. Pham Tien Nam NHD, Nguyen Khac Liem, Nguyen Tuan Hung, Dang Kim Khanh Ly, Hoang Van Minh. Anxiety among the Vietnamese Population during the COVID-19 Pandemic: Implications for Social Work Practice. *Social Work in Public Health* 2021;36(2) doi: doi.org/10.1080/19371918.2020.1871461
194. Naser AY, Dahmash EZ, Al-Rousan R, et al. Mental health status of the general population, healthcare professionals, and university students during 2019

coronavirus disease outbreak in Jordan: A cross-sectional study. *J Psychiatry Psychiatric Disord* 2020;10(8) doi: 10.1002/brb3.1730
195. Nayak BS, Sahu PK, Ramsaroop K, et al. Prevalence and factors associated with depression, anxiety and stress among healthcare workers of Trinidad and Tobago during COVID-19 pandemic: A cross-sectional study. *BMJ Open* 2021;11(4):e044397. doi: 10.1136/bmjopen-2020-044397
196. Ng BH, Nuratiqah N, Faisal A, et al. A descriptive study of the psychological experience of health care workers in close contact with a person with COVID-19. *The Medical Journal of Malaysia* 2020;75(5):485-89.
197. Ni MY, Yang L, Leung CMC, et al. Mental health, risk factors, and social media use during the COVID-19 epidemic and cordon sanitaire among the community and health professionals in Wuhan, China: Cross-sectional survey. *JMIR Ment Health* 2020;7(5):e19009. doi: doi:10.2196/19009
198. Ni Z, Lebowitz ER, Zou Z, et al. Response to the COVID-19 Outbreak in Urban Settings in China. *Journal of Urban Health* 2021;98:41-52. doi: doi.org/10.1007/s11524-020-00498-8
199. Ning X, Yu F, Huang Q, et al. The mental health of neurological doctors and nurses in Hunan Province, China during the initial stages of the COVID-19 outbreak. *BMC Psychiatry* 2020;20(436):1-9. doi: doi.org/10.1186/s12888-020-02838-z
200. S NN, Francis YM, K B, et al. A survey on anxiety and depression level among South Indian medical students during the COVID 19 pandemic. *Journal of the College of S106 Physicians and Surgeons Pakistan* 2020;11(SPL1):779 - 86. doi: 10.26452/ijrps.v11iSPL1.3082
201. Norhayati Mohd Noor RCY, Mohd Azman Yacob. Anxiety in Frontline and Non-Frontline Healthcare Providers in Kelantan, Malaysia. *IJERPH* 2021;18(3) doi: doi.org/10.3390/ijerph18030861
202. Salma SamirOmar WD, Noha Eid, Dalia Eldeeb, Amr Munir, Waleed Arafat. Psychological and Sexual Health During the COVID-19 Pandemic in Egypt: Are Women Suffering More? *Sexual Medicine* 2021;9 doi: doi.org/10.1016/j.esxm.2020.100295
203. Pan X, Xiao Y, Ren D, et al. Prevalence of mental health problems and associated risk factors among military healthcare workers in specialized COVID-19 hospitals in Wuhan, China: A cross-sectional survey. *Asia-Pacific Psychiatry* 2020:e12427. doi: doi.org/10.1111/appy.12427
204. Pan Y, Fang Y, Xin M, et al. Self-reported compliance with personal preventive measures among Chinese factory workers at the beginning of work resumption following the COVID-19 outbreak: Cross-sectional survey study. *Journal of Medical Internet Research* 2020;22(9):e22457. doi: 10.2196/22457
205. Wei Pan JH, Liangying Yi. Mental state of central sterile supply department staff during COVID-19 epidemic and CART analysis. *BMC Health Services Research* 2020;20 doi: doi.org/10.1186/s12913-020-05864-5
206. Pandey A, Sharma C, Chapagain RH, et al. Stress, Anxiety, Depression and Their Associated Factors among Health Care Workers During COVID-19 Pandemic in Nepal. *Journal of Traumatic Stress* 2020a;18(49):655 - 60. doi: 10.33314/jnhrc.v18i4.3190
207. Uma Pandey GC, Suruchi Mohan, Shuja Reagu, Shubham Kumar, Thomas Farrell. Anxiety, Depression and Behavioural Changes in Junior Doctors and Medical

Students Associated with the Coronavirus Pandemic: A Cross-Sectional Surve. *The Journal of Obstetrics and Gynecology of India* 2021;71:33-37. doi: doi.org/10.1007/s13224-020-01366-w

208. Passos L, Prazeres F, Teixeira A, et al. Impact on mental health due to covid-19 pandemic: Cross-sectional study in portugal and brazil. *International Journal of Environmental Research and Public Health* 2020;17(18):1-13. doi: 10.3390/ijerph17186794
209. Paz C, Mascialino G, Adana-Díaz L, et al. Anxiety and depression in patients with confirmed and suspected COVID-19 in Ecuador. *Psychiatr Clinical Neurosciences* 2020;74(10):554-55. doi: 10.1111/pcn.13106
210. Perera B, Wickramarachchi B, Samanmalie C, et al. Psychological experiences of healthcare professionals in Sri Lanka during COVID-19. *Asia Pacific Journal of Public Health* 2021;9(1) doi: 10.1186/s40359-021-00526-5
211. Perveen AH, Hazalizah, Othamn, Azizah, Ramlee, Fatanah. Prevalence of Anxiety, Stress, Depression among Malaysian Adults during COVID-19 Pandemic Movement Control Order. *Indian Journal of Community Health* 2020;32(3):579-81.
212. Puccinelli PJ, Costa TS, Seffrin A, et al. Physical Activity Levels and Mental Health during the COVID-19 Pandemic: Preliminary Results of a Comparative Study between Convenience Samples from Brazil and Switzerland. *Medicina* 2021;57(1):48. doi: 10.3390/medicina57010048
213. Qi J, Xu, J., Li, B.-Z., Huang, J.-S., Yang, Y., Zhang, Z.-T., . . . Zhang, X. The evaluation of sleep disturbances for Chinese frontline medical workers under the outbreak of COVID-19. *Sleep Medicine* 2020;72:1-4. doi: 10.1016/j.sleep.2020.05.023
214. Qian M, Wu Q, Wu P, et al. Anxiety levels, precautionary behaviours and public perceptions during the early phase of the COVID-19 outbreak in China: a population-based cross-sectional survey. *BMJ Open* 2020;10(10):e040910. doi: dx.doi.org/10.1136/bmjopen-2020-040910
215. Qiu J, Shen B, Zhao M, et al. A nationwide survey of psychological distress among Chinese people in the COVID-19 epidemic: Implications and policy recommendations. *General Psychiatry* 2020;33(2):e100213. doi: doi: 10.1136/gpsych-2020-100213
216. Que J, Shi L, Deng J, et al. Psychological impact of the COVID-19 pandemic on healthcare workers: a cross-sectional study in China. *Gen Psychiatr* 2020;33(3):e100259. doi: 10.1136/gpsych-2020-100259 [published Online First: 2020/07/01]
217. Ochilbek Rakhmanov AD, Senol Dane. A Brief Communication: Anxiety and Depression Levels in the Staff of a Nigerian Private University during COVID 19 Pandemic Outbreak. *Journal of Research in Medical and Dental Science* 2020;8(3):118-22.
218. V VR, Karri SR, Jezreel T, et al. Psychosocial Impact of COVID-19 Lockdown on Mental Wellbeing among 11 States of India: A Markov Modeling Approach. *Journal of Community Health* 2020;4(4) doi: 10.26502/jppd.2572-519X0103
219. Ren Z, Zhou Y, Liu YJBph. The psychological burden experienced by Chinese citizens during the COVID-19 outbreak: prevalence and determinants. *BMC Public Health* 2020;20(1):1-10. doi: doi.org/10.1186/s12889-020-09723-0

220. Ribeiro FS, Lessa JPA, Delmolin G, et al. Music Listening in Times of COVID-19 Outbreak: A Brazilian Study. *Frontiers in Psychology* 2021;12:10. doi: 10.3389/fpsyg.2021.647473
221. Robles R, Rodríguez E-R, Hamid, Álvarez-Icaza D, et al. Mental health problems among healthcare workers involved with the COVID-19 outbreak. *Brazilian Journal of Psychiatry* in press doi: 10.1590/1516-4446-2020-1346
222. Rogowska AM, Kuśnierz C, Bokszczanin A. Examining Anxiety, Life Satisfaction, General Health, Stress and Coping Styles During COVID-19 Pandemic in Polish Sample of University Students. *Psychology Research and Behavior Management* 2020;Volume 13:797-811. doi: 10.2147/PRBM.S266511
223. Rogowska AM, Pavlova I, Kuśnierz C, et al. Does Physical Activity Matter for the Mental Health of University Students during the COVID-19 Pandemic? *Journal of Clinical Medicine* 2020;9(11) doi: 10.3390/jcm9113494
224. Arshad AR, Islam F. COVID-19 and Anxiety amongst Doctors: A Pakistani Perspective. *Cureus* 2020:4. doi: 10.29271/jcpsp.2020.Supp3.S106
225. Saeed R, Amin F, Talha M, et al. COVID-19 Pandemic Prevalence and Risk Factors for Depression Among Health Care Workers in South Asia. *Perspect Psychiatr Care* 2021 doi: 10.1177/10105395211002324
226. Safa F, Anjum A, Hossain S, et al. Immediate psychological responses during the initial period of the COVID-19 pandemic among Bangladeshi medical students. *Children and Youth Services Review* 2021;122 doi: 10.1016/j.childyouth.2020.105912
227. Luis Sagaon-Teyssier AK, Adam Yattassaye, Fodié Diallo, Daniela Rojas Castroa, Rosemary Delabre. Assessment of mental health outcomes and associated factors among workers in community-based HIV care centers in the early stage of the COVID-19 outbreak in Mali. *Health Policy OPEN* 2020;1 doi: doi.org/10.1016/j.hpopen.2020.100017
228. Şahin MK, Aker S, Şahin G, et al. Prevalence of Depression, Anxiety, Distress and Insomnia and Related Factors in Healthcare Workers During COVID-19 Pandemic in Turkey. *National Journal of Physiology, Pharmacy and Pharmacology* 2020;45(6):1168 - 77. doi: 10.1007/s10900-020-00921-w
229. Samaniego A, Urzúa A, Buenahora M, et al. Sintomatología asociada a trastornos de salud mental en trabajadores sanitarios en Paraguay: efecto COVID-19. *Revista Interamericana de Psicología/Interamerican Journal of Psycholog* 2020;54(1):e1298-e98. doi: 10.30849/ripijp.v54i1.1298
230. Sandesh R, Shahid W, Dev K, et al. Impact of COVID-19 on the Mental Health of Healthcare Professionals in Pakistan. *Wiley* 2020;12(7) doi: 10.7759/cureus.8974
231. Schmitt Jr AA, Brenner AM, Primo de Carvalho Alves L, et al. Potential predictors of depressive symptoms during the initial stage of the COVID-19 outbreak among Brazilian adults. *Journal of Affective Disorders* 2021;282:1090-95. doi: 10.1016/j.jad.2020.12.203
232. Schuch FB, Bulzing RA, Meyer J, et al. Associations of moderate to vigorous physical activity and sedentary behavior with depressive and anxiety symptoms in self-isolating people during the COVID-19 pandemic: A cross-sectional survey in Brazil. *Psychiatry research* 2020;292:113339. doi: https://www.ncbi.nlm.nih.gov/pubmed/32745795

233. Scotta AV, Cortez MV, Miranda AR. Insomnia is associated with worry, cognitive avoidance and low academic engagement in Argentinian university students during the COVID-19 social isolation. *Psychology Health Medicine* 2021;2021:1-16. doi: 10.1080/13548506.2020.1869796
234. Secosan I, Virga D, Crainiceanu ZP, et al. The Mediating Role of Insomnia and Exhaustion in the Relationship between Secondary Traumatic Stress and Mental Health Complaints among Frontline Medical Staff during the COVID-19 Pandemic. *Behavioral Sciences* 2020;10(11):164. doi: 10.3390/bs10110164
235. Sabrine Sediri YZ, Sami Ouanes, Uta Ouali, Soumaya Bourgou, Rabaa Jomli, Fethi Nacef. Women's mental health: acute impact of COVID-19 pandemic on domestic violence. *Archives of Women's Mental Health* 2020;23:749-56. doi: doi.org/10.1007/s00737-020-01082-4
236. Serafim AP, Durães RS, Rocca CC, et al. Exploratory study on the psychological impact of COVID-19 on the general Brazilian population. *Plos one* 2021;16(2):e0245868. doi: 10.1371/journal.pone.0245868
237. Shi L, Lu Z-A, Que J-Y, et al. Prevalence of and risk factors associated with mental health symptoms among the general population in China during the coronavirus disease 2019 pandemic. *JAMA Network Open* 2020;3(7):e2014053-e53. doi: 10.1001/jamanetworkopen.2020.14053
238. Shrestha SL. Prevalence of Psychological Effect of COVID-19 on Medical Professionals in a Tertiary Care Center. *International Journal of Social Psychiatry* 2020;58(228) doi: 10.31729/jnma.5087
239. Hanaa A.M. Shuwiekh IAK, Mariam Sous Fahmy Sous, Jeffrey S. Ashby, Amthal Alhuwailah, Shadia Bint Ali Baali. The differential mental health impact of COVID-19 in Arab countries. *Current Psychology* 2020 doi: doi.org/10.1007/s12144-020-01148-7
240. Si M, Su X, Jiang Y, et al. The psychological impact of COVID-19 on medical care workers in China. *Infectious Diseases of Poverty* 2020;9(113) doi: doi.org/10.1186/s40249-020-00724-0
241. Sil A, Das A, Jaiswal S, et al. Mental health assessment of frontline COVID-19 dermatologists: A Pan-Indian multicentric cross-sectional study. *International Journal of Mental Health and Addiction* 2020;33(6) doi: 10.1111/dth.13884
242. Medical student FoM, University of Sarajevo, Sarajevo, Bosnia and Hercegovina, Sljivo A, Kacamakovic M, et al. FEAR AND DEPRESSION AMONG RESIDENTS OF BOSNIA AND HERZEGOVINA DURING COVID-19 OUTBREAK - INTERNET SURVEY. *Psychiatria Danubina* 2020;32(2):266-72. doi: 10.24869/psyd.2020.266
243. Sögüt S, Dolu İ, Cangöl E. The relationship between COVID-19 knowledge levels and anxiety states of midwifery students during the outbreak: A cross-sectional web-based survey. *Turk Neurosurg* 2021;57(1):246 - 52. doi: 10.1111/ppc.12555
244. Song X, Fu, W., Liu, X., Luo, Z., Wang, R., Zhou, N., . . . Lv, C. . Mental health status of medical staff in emergency departments during the Coronavirus disease 2019 epidemic in China. *Brain Behav Immun* 2020;88:60-65. doi: 10.1016/j.bbi.2020.06.002 [published Online First: 2020/06/09]
245. Song L, Wang Y, Li Z, et al. Mental health and work attitudes among people resuming work during the Covid-19 pandemic: A cross-sectional study in China. *IJERPH* 2020;17(14):5059. doi: doi.org/10.3390/ijerph17145059

246. A. Souilhi AGF, Letaief-Ksontini, N. Chraiet, Y. Yahiaoui, F. Mghirbi. Psychological impact of the COVID-19 pandemic on health care workers in oncology in Tunisia. *Annals of Oncology* 2020;31(4) doi: doi.org/10.1016/j.annonc.2020.08.2055
247. Souza ASR, Souza GFA, Souza GA, et al. Factors associated with stress, anxiety, and depression during social distancing in Brazil. *Revista de saude publica* 2021;55:5. doi: 10.11606/s1518-8787.2021055003152
248. Ganapathy Sankar U MR. Impact of covid 19 on the mental health and qulaity of sleep among health care professionals. *International Journal of Pharmaceutical Research* 2020;12(3):2577-80.
249. Su J, Chen X, Yang N, et al. Proximity to people with COVID-19 and anxiety among community residents during the epidemic in Guangzhou, China. *BJPsych Open* 2020;6(4) doi: doi.org/10.1192/bjo.2020.59
250. Sun L, Sun, Z., Wu, L., Zhu, Z., Zhang, F., Shang, Z., . . . Liu, W. Prevalence and Risk Factors of Acute Posttraumatic Stress Symptoms during the COVID-19 Outbreak in Wuhan, China. *Journal of Affective Disorders* 2020;283:123-29. doi: 10.1101/2020.03.06.20032425
251. Sun H, Wang S, Wang W, et al. Correlation between emotional intelligence and negative emotions of front-line nurses during the COVID-19 epidemic: a cross-sectional study. *Journal of Clinical Nursing* 2020;30(3-4):385-96. doi: doi.org/10.1111/jocn.15548
252. Sun Q, Lu NJIjoer, health p. Social Capital and Mental Health among Older Adults Living in Urban China in the Context of COVID-19 Pandemic. *IJERPH* 2020;17(21):7947. doi: doi.org/10.3390/ijerph17217947
253. Sun Y, Song H, Liu H, et al. Occupational stress, mental health, and self-efficacy among community mental health workers: A cross-sectional study during COVID-19 pandemic. *International Journal of Social Psychiatry* 2020:0020764020972131. doi: doi.org/10.1177/0020764020972131
254. Sun Yuyan WD, Han Ziting, Gao Jie, Zhu Shanshan, Zhang Huimin. Disease Prevention Knowledge, Anxiety, and Professional Identity during COVID-19 Pandemic in Nursing Students in Zhengzhou, China. *Journal of Korean Academy of Nursing* 2020;50(4):533-40. doi: doi.org/10.4040/jkan.20125
255. Sundarasen S, Chinna K, Kamaludin K, et al. Psychological impact of COVID-19 and lockdown among university students in Malaysia: Implications and policy recommendations. *International journal of environmental research and public health* 2020;17(17):6206.
256. Deni Kurniadi Sunjaya DMDH, Adiatma Y. M. Siregar. Depressive, anxiety, and burnout symptoms on health care personnel at a month after COVID-19 outbreak in Indonesia. *BMC Public Health* 2021;21(227) doi: doi.org/10.1186/s12889-021-10299-6
257. Suryavanshi N, Kadam A, Dhumal G, et al. Mental health and quality of life among healthcare professionals during the COVID-19 pandemic in India. *Brain and Behavior* 2020;10(11) doi: 10.1002/brb3.1837
258. Tan W, Hao F, McIntyre RS, et al. Is returning to work during the COVID-19 pandemic stressful? A study on immediate mental health status and psychoneuroimmunity prevention measures of Chinese workforce. *Brain Behav Immun* 2020;87:84-92. doi: 10.1016/j.bbi.2020.04.055 [published Online First: 2020/04/27]

259. Tasnim R, Islam MS, Sujan MSH, et al. Suicidal ideation among Bangladeshi university students early during the COVID-19 pandemic: Prevalence estimates and correlates. *Children and Youth Services Review* 2020;119 doi: 10.1016/j.childyouth.2020.105703
260. Temsah M-H, Al-Sohime F, Alamro N, et al. The psychological impact of COVID-19 pandemic on health care workers in a MERS-CoV endemic country. *Journal of Infection and Public Health* 2020;13(6):877 - 82. doi: 10.1016/j.jiph.2020.05.021
261. Teng Z, Wei Z, Qiu Y, et al. Psychological status and fatigue of frontline staff two months after the COVID-19 pandemic outbreak in China: A cross-sectional study. *Journal of Affective Disorders* 2020;275:247-52. doi: 10.1016/j.jad.2020.06.032
262. Abinet Teshome MG, Mulugeta Shegaze, Beemnet Tekabe, Asmare Getie, Genet Assefa, Dinkalem Getahun, Tesfaye Kanko, Tamiru Getachew, Nuhamin Yenesew, Zebene Temtmie, and Kabtamu Tolosie. Generalized Anxiety Disorder and Its Associated Factors Among Health Care Workers Fighting COVID-19 in Southern Ethiopia. *Psychology Research and Behavior Management* 2020;13:907-17. doi: 10.2147/PRBM.S282822
263. Torrente F, Yoris A, Low D, et al. Sooner than you think: A very early affective reaction to the COVID-19 pandemic and quarantine in Argentina. *Journal of affective disorders* 2021;282:495-503. doi: 10.1016/j.jad.2020.12.124
264. Torrente F, Yoris A, Low D, et al. Emotional symptoms, mental fatigue and behavioral adherence after 72 continuous days of strict lockdown during the COVID-19 pandemic in Argentina. *medRxiv* 2021:21255866. doi: 10.1101/2021.04.21.21255866 [published Online First: 2021.04.21]
265. Tien V Tran HCN, Linh V Pham, Minh H Nguyen, Huu Cong Nguyen, Tung H Ha. Impacts and interactions of COVID-19 response involvement, health-related behaviours, health literacy on anxiety, depression and health-related quality of life among healthcare workers: a cross-sectional study. *BMJ Open* 2020;10(12) doi: dx.doi.org/10.1136/bmjopen-2020-041394
266. Tu ZH, He JW, Zhou N. Sleep quality and mood symptoms in conscripted frontline nurse in Wuhan, China during COVID-19 outbreak: A cross-sectional study. *Medicine (Baltimore)* 2020;99(26):e20769. doi: 10.1097/MD.0000000000020769 [published Online First: 2020/06/27]
267. Vala N, Vachhani M, Sorani A. Study of anxiety, stress, and depression level among medical students during covid-19 pandemic phase in jamnagar city. *National Journal of Physiology, Pharmacy and Pharmacology* 2020 doi: 10.5455/njppp.2020.10.07205202031072020
268. Venugopal VC, Mohan A, Chennabasappa LK. Status of mental health and its associated factors among the general populace of India during COVID-19 pandemic. *ASIA-PACIFIC PSYCHIATRY* 2020 doi: 10.1111/appy.12412
269. Verma S, Mishra A. Depression, anxiety, and stress and sociodemographic correlates among general Indian public during COVID-19. *SAGE journals* 2020;66(8):756 - 62. doi: 10.1177/0020764020934508
270. Villela EFD, da Cunha IR, Fodjo JNS, et al. Impact of COVID-19 on Healthcare Workers in Brazil between August and November 2020: A Cross-Sectional Survey. *International Journal of Environmental Research and Public Health* 2021;18(12): 6511-22. doi: 10.3390/ijerph18126511

271. Vitorino LM, Yoshinari GH, Gonzaga G, et al. Factors associated with mental health and quality of life during the COVID-19 pandemic in Brazil. *Bjpsych Open* 2021;7(e103):1-8. doi: 10.1192/bjo.2021.62
272. Vujčić I, Safiye T, Milikić B, et al. Coronavirus Disease 2019 (COVID-19) Epidemic and Mental Health Status in the General Adult Population of Serbia: A Cross-Sectional Study. *International Journal of Environmental Research and Public Health* 2021;18(4):1957. doi: 10.3390/ijerph18041957
273. Chongying Wang HZ, Haoran Zhang. Chinese College Students Have Higher Anxiety in New Semester of Online Learning During COVID-19: A Machine Learning Approach. *Frontiers in Psychology* 2020 doi: doi.org/10.3389/fpsyg.2020.587413
274. Chongying Wang HZ. The Impact of COVID-19 on Anxiety in Chinese University Students. *Frontiers in Psychology* 2020 doi: doi.org/10.3389/fpsyg.2020.01168
275. Wang Y, Di Y, Ye J, et al. Study on the public psychological states and its related factors during the outbreak of coronavirus disease 2019 (COVID-19) in some regions of China. *Psychol Health Med* 2020:1-10. doi: 10.1080/13548506.2020.1746817 [published Online First: 2020/04/01]
276. Wang S, Xie L, Xu Y, et al. Sleep disturbances among medical workers during the outbreak of COVID-2019. *Occup Med (Lond)* 2020;70(5):364-69. doi: 10.1093/occmed/kqaa074 [published Online First: 2020/05/07]
277. Wang H, Huang D, Huang H, et al. The psychological impact of COVID-19 pandemic on medical staff in Guangdong, China: a cross-sectional study. *Psychol Med* 2020:1-9. doi: 10.1017/S0033291720002561 [published Online First: 2020/07/07]
278. Wang J, Gong Y, Chen Z, et al. Sleep disturbances among Chinese residents during the Coronavirus Disease 2019 outbreak and associated factors. 2020;74:199-203.
279. Wang L-Q, Zhang M, Liu G-M, et al. Psychological impact of coronavirus disease (2019)(COVID-19) epidemic on medical staff in different posts in China: A multicenter study. *Journal of Psychiatric Research* 2020;129:198-205. doi: doi.org/10.1016/j.jpsychires.2020.07.008
280. Wang S, Zhang Y, Ding W, et al. Psychological distress and sleep problems when people are under interpersonal isolation during an epidemic: a nationwide multicenter cross-sectional study. *European Psychiatry* 2020;63(1) doi: doi.org/10.1192/j.eurpsy.2020.78
281. Wang W, Song W, Xia Z, et al. Sleep disturbance and psychological profiles of medical staff and non-medical staff during the early outbreak of COVID-19 in Hubei Province, China. *Frontiers in Psychology* 2020;11:733. doi: doi.org/10.3389/fpsyt.2020.00733
282. Wang Y-X, Guo H-T, Du X-W, et al. Factors associated with post-traumatic stress disorder of nurses exposed to corona virus disease 2019 in China. *Medicine* 2020;99(26) doi: 10.1097/MD.0000000000020965
283. Wang Y, Hu Z, Feng Y, et al. Changes in network centrality of psychopathology symptoms between the COVID-19 outbreak and after peak. *Molecular Psychiatry* 2020;25(12):3140-49. doi: doi.org/10.1038/s41380-020-00881-6
284. Wang Y, Ma S, Yang C, et al. Acute psychological effects of Coronavirus Disease 2019 outbreak among healthcare workers in China: a cross-sectional study.

Translational Psychiatry 2020;10(1):1-10. doi: doi.org/10.1038/s41398-020-01031-w

285. Wang H, Xia Q, Xiong Z, et al. The psychological distress and coping styles in the early stages of the 2019 coronavirus disease (COVID-19) epidemic in the general mainland Chinese population: A web-based survey. *PLOS ONE* 2020;15(4):e0233410. doi: https://doi.org/10.1371/journal.pone.0233410
286. Ying Wang SM, Can Yang, Zhongxiang Cai, Shaohua Hu, Bin Zhang. Anxiety and Sleep Problems of College Students During the Outbreak of COVID-19. *Translational Psychiatry* 2020;10(348) doi: doi.org/10.1038/s41398-020-01031-w
287. Wańkowicz P, Szylińska A, Rotter I. Assessment of Mental Health Factors among Health Professionals Depending on Their Contact with COVID-19 Patients. *International Journal of Environmental Research and Public Health* 2020;17(16):5849. doi: 10.3390/ijerph17165849
288. Werneck AO, Silva DR, Malta DC, et al. Physical inactivity and elevated TV-viewing reported changes during the COVID-19 pandemic are associated with mental health: A survey with 43,995 Brazilian adults. *Journal of Psychosomatic Research* 2021;140:110292. doi: 10.1016/j.jpsychores.2020.110292
289. Wilson W, Raj JP, Rao S, et al. Prevalence and Predictors of Stress, anxiety, and Depression among Healthcare Workers Managing COVID-19 Pandemic in India: a Nationwide Observational Study. *SAGE journals* 2020;42(4):353 - 58. doi: 10.1177/0253717620933992
290. Wong LP, Alias H. Temporal changes in psychobehavioural responses during the early phase of the COVID-19 pandemic in Malaysia. *Journal of behavioral medicine* 2021;44(1):18-28.
291. Wu M, Han H, Lin T, et al. Prevalence and risk factors of mental distress in China during the outbreak of COVID-19: A national cross-sectional survey. *Brain and Behavior* 2020;10(11):e01818. doi: doi.org/10.1002/brb3.1818
292. Wu S, Li Z, Li Z, et al. The mental state and risk factors of Chinese medical staff and medical students in early stages of the COVID-19 epidemic. *Comprehensive Psychiatry* 2020;102:152202. doi: doi.org/10.1016/j.comppsych.2020.152202
293. Wu L, Guo X, Shang Z, et al. China experience from COVID-19: Mental health in mandatory quarantine zones urgently requires intervention. *Psychological Trauma: Theory, Research, Practice, and Policy* 2020;12(1):3-5. doi: doi.org/10.1037/tra0000609
294. Ming-Qiang Xiang X-MT, Jian Sun, Hai-Yan Yang, Xue-Ping Zhao, Lei Liu, Xiao-Hui Hou. Relationship of Physical Activity With Anxiety and Depression Symptoms in Chinese College Students During the COVID-19 Outbreak. *Frontiers in Psychology* 2020 doi: 10.3389/fpsyg.2020.582436
295. Xiao X, Zhu X, Fu S, et al. Psychological impact of healthcare workers in China during COVID-19 pneumonia epidemic: a multi-center cross-sectional survey investigation. *Journal of Affective Disorders* 2020;274:405-10. doi: doi.org/10.1016/j.jad.2020.05.081
296. Huidi Xiao WS, Menglong Li, Ziang Li, Fangbiao Tao, Xiaoyan Wu. Social Distancing among Medical Students during the 2019 Coronavirus Disease Pandemic in China: Disease Awareness, Anxiety Disorder, Depression, and Behavioral Activities. *IJERPH* 2020;17(14) doi: doi.org/10.3390/ijerph17145047

297. Xing L-q, Xu M-l, Sun J, et al. Anxiety and depression in frontline health care workers during the outbreak of Covid-19. *International Journal of Social Psychiatry* 2020:0020764020968119. doi: doi.org/10.1177/0020764020968119
298. Xiong H, Yi S, Lin YJITJoHCO, Provision,, et al. The psychological status and self-efficacy of nurses during COVID-19 outbreak: a cross-sectional survey. *The Journal of Health Care Organization, Provision, and Financing* 2020;57:0046958020957114. doi: doi.org/10.1177/0046958020957114
299. Xiaoming X, Ming A, Su H, et al. The psychological status of 8817 hospital workers during COVID-19 Epidemic: A cross-sectional study in Chongqing. *Journal of Affective Disorders* 2020;276:555-61. doi: doi.org/10.1016/j.jad.2020.07.092
300. Yáñez JA, Jahanshahi AA, Alvarez-Risco A, et al. Anxiety, Distress, and Turnover Intention of Healthcare Workers in Peru by Their Distance to the Epicenter during the COVID-19 Crisis. *The American Journal of Tropical Medicine and Hygiene* 2020;103(4):1614 - 20. doi: https://doi.org/10.4269/ajtmh.20-0800
301. Yang X, Zhang Y, Li S, et al. Risk factors for anxiety of otolaryngology healthcare workers in Hubei province fighting coronavirus disease 2019 (COVID-19). *Social Psychiatry and Psychiatric Epidemiology* 2020;56(56):39-45. doi: doi.org/10.1007/s00127-020-01928-3
302. Yang Y, Zhu J-f, Yang S-y, et al. Prevalence and associated factors of poor sleep quality among Chinese returning workers during the COVID-19 pandemic. *Sleep Medicine* 2020;73:47-52. doi: doi.org/10.1016/j.sleep.2020.06.034
303. Yıldırım M, Özaslan A. Worry, Severity, Controllability, and Preventive Behaviours of COVID-19 and Their Associations with Mental Health of Turkish Healthcare Workers Working at a Pandemic Hospital. *International Journal of Mental Health and Addiction* 2021 doi: 10.1007/s11469-021-00515-0
304. Yilmaz A, Karakoyun DO, Isik HS, et al. The Effect of the COVID-19 Pandemic on Functioning of Neurosurgery Clinics and the Anxiety Levels of Neurosurgeons in Turkey. *Turk Neurosurg* 2020;30(6):944 - 51. doi: 10.5137/1019-5149.JTN.31940-20.2
305. Yin X, Wang J, Feng J, et al. The Impact of the Corona Virus Disease 2019 Outbreak on Chinese Residents' Mental Health. *The Lancet* 2020 doi: dx.doi.org/10.2139/ssrn.3556680
306. Yin Q, Sun Z, Liu T, et al. Posttraumatic Stress Symptoms of Health Care Workers during the Corona Virus Disease 2019 (COVID-19). *Clinical Psychology & Psychology* 2020;27(3):384-95. doi: doi.org/10.1002/cpp.2477
307. Ying Y, Ruan L, Kong F, et al. Mental health status among family members of health care workers in Ningbo, China, during the coronavirus disease 2019 (COVID-19) outbreak: a cross-sectional study. *BMC Psychiatry* 2020;20(1):1-10. doi: doi.org/10.1186/s12888-020-02784-w
308. Yitayih Y, Mekonen S, Zeynudin A, et al. Mental health of healthcare professionals during the early stage of the COVID-19 pandemic in Ethiopia. *The British Journal of Psychiatry* 2020;7(e1):1-6. doi: 10.1192/bjo.2020.130
309. Youssef Naglaa MA, Ezzat Reem, Yosef Mostafa, El Kassas Mohamed. Mental health status of health-care professionals working in quarantine and non-quarantine Egyptian hospitals during the COVID-19 pandemic. *East Mediterr Health J* 2020;26(10):1155-64.

310. Yu H, Li M, Li Z, et al. Coping style, social support and psychological distress in the general Chinese population in the early stages of the COVID-2019 epidemic. *SSRN* 2020 doi: http://dx.doi.org/10.2139/ssrn.3556633
311. Yu BY-M, Yeung W-F, Lam JC-S, et al. Prevalence of sleep disturbances during COVID-19 outbreak in an urban Chinese population: a cross-sectional study. *Sleep Medicine* 2020;74:18-24. doi: https://doi.org/10.1016/j.sleep.2020.07.009
312. Xu H, Li Y, Zeng M, et al. Psychological Behavior of Frontline Medical Staff in the Use of Preventive Medication for COVID-19: A Cross-Sectional Study. *Frontiers in Psychology* 2020;11:2104. doi: 10.3389/fpsyg.2020.02104
313. Mingli Yu FT, Qi Cui, Hui Wu. Prevalence and its associated factors of depressive symptoms among Chinese college students during the COVID-19 pandemic. *BMC Psychiatry* 2021;21(66) doi: doi.org/10.1186/s12888-021-03066-9
314. Zhan Y-x, Zhao S-y, Yuan J, et al. Prevalence and Influencing Factors on Fatigue of First-line Nurses Combating with COVID-19 in China: A Descriptive Cross-Sectional Study. *Current Medical Science* 2020;40(4):625-35. doi: doi.org/10.1007/s11596-020-2226-9
315. Zhan Y, Liu Y, Liu H, et al. Factors associated with insomnia among Chinese front-line nurses fighting against COVID-19 in Wuhan: A cross-sectional survey. *Journal of Nursing Management* 2020;28(7):1525-35. doi: doi.org/10.1111/jonm.13094
316. Zhang C, Yang L, Liu S, et al. Survey of Insomnia and Related Social Psychological Factors Among Medical Staff Involved in the 2019 Novel Coronavirus Disease Outbreak. *Front Psychiatry* 2020;11:306. doi: 10.3389/fpsyt.2020.00306 [published Online First: 2020/04/30]
317. Zhang J, Lu H, Zeng H, et al. The differential psychological distress of populations affected by the COVID-19 pandemic. *Brain, Behavior, and Immunity* 2020;87:49–50. doi: doi: 10.1016/j.bbi.2020.04.031
318. Zhang SX, Wang Y, Rauch A, et al. Unprecedented disruption of lives and work: Health, distress and life satisfaction of working adults in China one month into the COVID-19 outbreak. *Psychiatry research* 2020;288:112958.
319. Zhang WR, Wang K, Yin L, et al. Mental Health and Psychosocial Problems of Medical Health Workers during the COVID-19 Epidemic in China. *Psychother Psychosom* 2020;89(4):242-50. doi: 10.1159/000507639 [published Online First: 2020/04/10]
320. Zhang Y, Ma ZF. Impact of the COVID-19 Pandemic on Mental Health and Quality of Life among Local Residents in Liaoning Province, China: A Cross-Sectional Study. *International Journal of Environmental Research and Public Health* 2020;17(7):2381. doi: doi: 10.3390/ijerph17072381
321. Zhang C, Peng D, Lv L, et al. Individual Perceived Stress Mediates Psychological Distress in Medical Workers During COVID-19 Epidemic Outbreak in Wuhan. *Neuropsychiatric Disease and Treatment* 2020;16:2529. doi: 10.2147/NDT.S266151
322. Zhang H, Shi Y, Jing P, et al. Posttraumatic stress disorder symptoms in healthcare workers after the peak of the COVID-19 outbreak: A survey of a large tertiary care hospital in Wuhan. *Psychiatry Research* 2020;294:113541. doi: doi.org/10.1016/j.psychres.2020.113541
323. Zhang W, Yang X, Zhao J, et al. Depression and Psychological-Behavioral Responses Among the General Public in China During the Early Stages of the

COVID-19 Pandemic: Survey Study. *Journal of Medical Internet Research* 2020;22(9):e22227. doi: preprints.jmir.org/preprint/22227

324. Zhang X-R, Huang Q-M, Wang X-M, et al. Prevalence of anxiety and depression symptoms, and association with epidemic-related factors during the epidemic period of COVID-19 among 123,768 workers in China: A large cross-sectional study. *Journal of Affective Disorders* 2020;277:495-502. doi: doi.org/10.1016/j.jad.2020.08.041
325. Zhang Y, Wang S, Ding W, et al. Status and influential factors of anxiety depression and insomnia symptoms in the work resumption period of COVID-19 epidemic: A multicenter cross-sectional study. *Journal of Psychosomatic Research* 2020;138:110253. doi: doi.org/10.1016/j.jpsychores.2020.110253
326. Yao Zhang HZ, Xindong Ma, Qian Di. Mental Health Problems during the COVID-19 Pandemics and the Mitigation Effects of Exercise: A Longitudinal Study of College Students in China. *IJERPH* 2020;17(10) doi: doi.org/10.3390/ijerph17103722
327. Zhang SX, Wang Y, Jahanshahi AA, et al. A Brief Report On The Mental Distress Of Peruvian Adults During The Onset Stage of the COVID-19 Pandemic. *medRxiv* 2020 doi: https://doi.org/10.1101/2020.04.18.20070896
328. Zhang SX, Huang H, Li J, et al. Predictors of Depression and Anxiety Symptoms in Brazil during COVID-19. *International Journal of Environmental Research and Public Health* 2021;18(3):7026.
329. Zhang SX, Wang Y, Jahanshahi AA, et al. Early evidence and predictors of mental distress of adults one month in the COVID-19 epidemic in Brazil. *Journal of Psychosomatic Research* 2021;142:110366. doi: https://doi.org/10.1016/j.jpsychores.2021.110366
330. Zhang SX, Chen J, Jahanshahi AA, et al. Succumbing to the COVID-19 pandemic—Healthcare workers not satisfied and intend to leave their jobs. *International Journal of Environmental Research and Public Health* 2021;2021:1-10. doi: https://doi.org/10.1007/s11469-020-00418-6
331. Zhao K, Zhang G, Feng R, et al. Anxiety, depression and insomnia: A cross-sectional study of frontline staff fighting against COVID-19 in Wenzhou, China. *Psychiatry Research* 2020;292:113304. doi: 10.1016/j.psychres.2020.113304
332. Zhao SZ, Wong JYH, Luk TT, et al. Mental health crisis under COVID-19 pandemic in Hong Kong, China. *International Journal of Infectious Diseases* 2020;100:431-33. doi: doi.org/10.1016/j.ijid.2020.09.030
333. Zhao X, Lan M, Li H, et al. Perceived Stress and Sleep Quality Among the Non-diseased General Public in China During the 2019 Coronavirus Disease: A Moderated Mediation Model. *Sleep Medicine* 2020;77:339-45. doi: doi.org/10.1016/j.sleep.2020.05.021
334. Yuqing Zhao YA, Xing Tan, Xiaohui Li. Mental Health and Its Influencing Factors among Self-Isolating Ordinary Citizens during the Beginning Epidemic of COVID-19. *Journal of Loss and Trauma* 2020;25(6-7):580-93. doi: doi.org/10.1080/15325024.2020.1761592
335. Zhou Y, Yang Y, Shi T, et al. Prevalence and Demographic Correlates of Poor Sleep Quality Among Frontline Health Professionals in Liaoning Province, China During the COVID-19 Outbreak. *Front Psychiatry* 2020;11:520. doi: 10.3389/fpsyt.2020.00520 [published Online First: 2020/07/01]

336. Zhou Y, Wang W, Sun Y, et al. The prevalence and risk factors of psychological disturbances of frontline medical staff in china under the COVID-19 epidemic: Workload should be concerned. *Journal of Affective Disorders* 2020;277:510-14. doi: doi.org/10.1016/j.jad.2020.08.059
337. Zhu Z, Xu, S., Wang, H., Liu, Z., Wu, J., Li, G., . . . Wang, W. COVID-19 in Wuhan: Immediate Psychological Impact on 5062 Health Workers. *MedRxiv* 2020 doi: 10.1101/2020.02.20.20025338
338. Zhu W, Wei Y, Meng X, et al. The mediation effects of coping style on the relationship between social support and anxiety in Chinese medical staff during COVID-19. *BMC Health Services Research* 2020;20(1):1-7. doi: doi.org/10.1186/s12913-020-05871-6
339. Zhu J, Sun L, Zhang L, et al. Prevalence and Influencing Factors of Anxiety and Depression Symptoms in the First-Line Medical Staff Fighting Against COVID-19 in Gansu. *Front Psychiatry* 2020;11:386. doi: 10.3389/fpsyt.2020.00386 [published Online First: 2020/05/16]
340. Zhu S, Wu Y, Zhu CY, et al. The immediate mental health impacts of the COVID-19 pandemic among people with or without quarantine managements. *Brain Behav Immun* 2020;87:56-58. doi: 10.1016/j.bbi.2020.04.045 [published Online First: 2020/04/22]
341. Zubayer AA, Rahman ME, Islam MB, et al. Psychological states of Bangladeshi people four months after the COVID-19 pandemic: An online survey. *Heliyon* 2020;6(9) doi: 10.1016/j.heliyon.2020.e05057
